# Climate change impacts and costs to U.S. electricity transmission and distribution infrastructure

**DOI:** 10.1016/j.energy.2020.116899

**Published:** 2020-03-15

**Authors:** Charles Fant, Brent Boehlert, Kenneth Strzepek, Peter Larsen, Alisa White, Sahil Gulati, Yue Li, Jeremy Martinich

**Affiliations:** a Industrial Economics, Inc., 2067 Massachusetts Ave, Cambridge, MA, 02140, USA; b Massachusetts Institute of Technology, 77 Massachusetts Ave, Cambridge, MA, 02139, USA; c Carroll College, 1601 N Benton Ave., Helena, MT, 59625, USA; d Case Western Reserve University, Nord 500, Cleveland, OH, 44106, USA; e U.S. Environmental Protection Agency, 1200 Pennsylvania Ave, NW MC6207A, Washington, DC, USA

**Keywords:** Climate change, Infrastructure, Transmission, Distribution

## Abstract

This study presents a screening-level analysis of the impacts of climate change on electricity transmission and distribution infrastructure of the U.S. In particular, the model identifies changes in performance and longevity of physical infrastructure such as power poles and transformers, and quantifies these impacts in economic terms. This analysis was evaluated for the contiguous U.S, using five general circulation models (GCMs) under two greenhouse gas emission scenarios, to analyze changes in damage and cost from the baseline period to the end of the century with three different adaptation strategies. Total infrastructure costs were found to rise considerably, with annual climate change expenditures increasing by as much as 25%. The results demonstrate that climate impacts will likely be substantial, though this analysis only captures a portion of the total potential impacts. A proactive adaptation strategy resulted in the expected costs of climate change being reduced by as much as 50% by 2090, compared to a scenario without adaptation. Impacts vary across the contiguous U.S. with the highest impacts in parts of the Southeast and Northwest. Improvements and extensions to this analysis would help better inform climate resiliency policies and utility-level planning for the future.

## 1. Introduction

Weather-related events, such as ice, high winds, flooding, and lightning strikes, cause about 78% of major power interruptions in the U.S. power distribution system [1] (based on 1333 events from 1992 to 2010). For many people, such power interruptions serve as the first tangible sign of the inter-related nature of weather and existing electric distribution and transmission systems. Weather-related power interruptions are exceptionally costly, and a large fraction of interruptions result from extreme, low probability events [2]. While power interruptions usually lead to short-term impacts, weather and climate are also linked to longer-term impacts on the power grid, such as reductions in infrastructure lifespan or power line ampacity [3].

The distribution system in the U.S. is worth trillions of dollars, so the capital stock at risk of weather-related impacts is substantial. Infrastructure is typically built based on design standards that use observed historic weather data to determine the size and/or type of structure needed to withstand probable future weather events (e.g., in distribution pole design, see Refs. [4,5]). As climate change increasingly alters climate conditions and patterns away from observed history, this traditional approach to infrastructure design may no longer be adequate, leading to power interruptions and other physical impacts to the electric grid to change in frequency and/or duration, and imposing unexpected costs.

A major consideration in assessing the impacts of climate change on U.S. electricity transmission and distribution infrastructure is the rate of climate change compared to the lifecycle of power system infrastructure replacement under “normal” conditions. The lifespan of certain elements of power system infrastructure can be up to several decades [6]. For example, the typical lifespan of a large transformer is about 40 years and a new transmission line can take a decade to plan and permit [7]. Thus, infrastructure design decisions made today will ultimately have to cope with weather conditions experienced several decades from now.

The IPCC Working Group III identifies the need for physical impacts of climate change on the energy sector [8]. While work has been done that looks at the impacts of extreme weather stressors on power systems [9–14], the literature on electric transmission and distribution infrastructure is scarce [15]. Certain studies have analyzed the impacts of high temperatures in the U.S. (CONUS: [3]; California: Saythaye et al., 2011, Northeast: [16]). Larsen et al. [17] focus on impacts on electricity grid reliability, while other studies examine impacts on physical electricity infrastructure [3,18,19]. The study presented here quantifies climate change impacts to the electricity transmission and distribution system by combining the focus on costing the impacts of climate change used by Larsen et al. [17] with the emphasis on physical impacts to electricity grid infrastructure, similar to the Sathaye et al. [19] evaluation of impacts for California.

In this study, we develop a screening-level model to estimate the infrastructure-related impacts (positive or negative) of climate change, covering many of the direct costs to infrastructure that are likely to cause the highest impact by the end of the century. We utilize atmosphere-ocean general circulation models (AOGCMs or GCMs) under two greenhouse gas (GHG) emission scenarios to analyze changes in weather from the baseline period to 2099, and analyze the implications of different adaptation strategies. Since this is a screening-level analysis completed on a vast spatial scale, the results of this study are not intended for project-level or utility-level planning. Rather, the objective of the study is to analyze and characterize climate change costs across multiple dimensions—by stressor, region, effects of GHG mitigation, and for different adaptation strategies—for the sake of informing policy analysis and to inspire further, more detailed local analysis. While the impacts of climate change on electricity generation or the fuel sector may be significant—such as solar, wind, hydropower, thermal cooling facilities, or pipelines—the focus of this study is specifically on impacts to electricity transmission and distribution infrastructure.

## 2. Methodological approach

This study quantifies climate change impacts to electricity transmission and distribution infrastructure across the CONUS through a screening-level modelling analysis. Physical and economic impacts from damages to these infrastructure systems are estimated using stressor-response functions that relate different climate stressors to the response of the various relevant transmission and distribution infrastructure components. The analysis is evaluated for each of the 3109 counties in the CONUS.

**Fig. 1.**
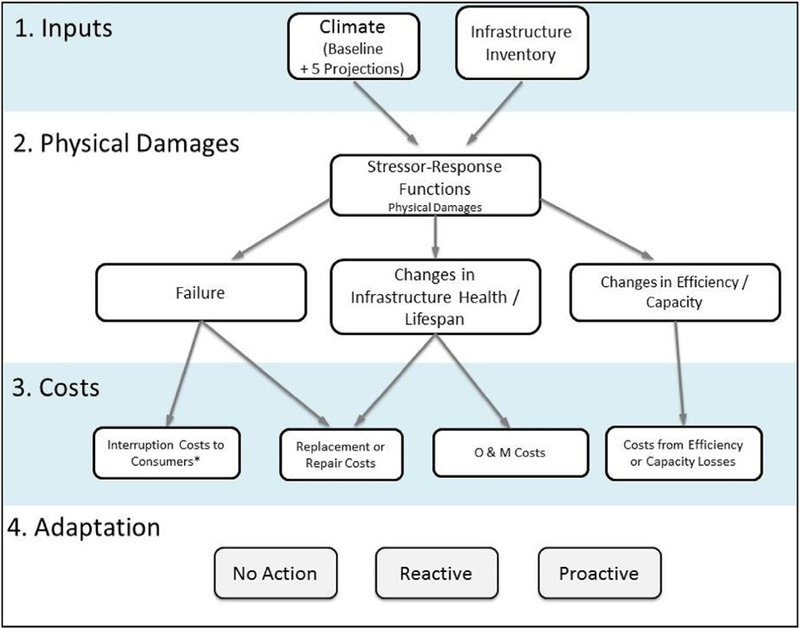
Model Flow Diagram (*Note: Interruption cost approximations are presented in the discussion).

The overall steps of the screening-level analysis are shown in Fig. 1: first, climate inputs and an infrastructure inventory are supplied to our model. Second, physical damages to the infrastructure inventory are estimated using climate-driven stressor-response functions. Third, economic impacts of these physical damages are quantified. Finally, the performance of different adaptation options is evaluated.

Model inputs (Step 1) are in the form of climate information from various GCMs and details of the energy infrastructure inventory in the U.S. Section 2.1 discusses these inputs in more detail.

Estimation of physical damages to the inventory (Step 2), as represented by stressor-response functions, forms the heart of this analysis. We use process-based, engineering-type approaches to construct these stressor-response relationships (see Section 2.2). The stressor-response functions help determine the infrastructure’s physical “response” to climate stressors. These functions capture three different types of physical damage: (1) infrastructure can fail due to abrupt extreme events, resulting in power interruptions and/or necessitating repair or replacement; (2) infrastructure can deteriorate due to changes in wear and tear on the infrastructure caused by weather (i.e. lifespan reduction); and/or (3) climate can necessitate changes in transmission and distribution capacity (e.g., high ambient temperatures reduce the allowable current in power lines).

We do not include all possible climate change stressors, with many excluded because of insignificant associated costs (e.g., the impact of air temperature on transmission towers). Conversely, some relationships that are important for the assessment of physical damages are necessarily excluded because the climate stressor is uncertain or the infrastructure damage estimation is too uncertain given the information available at the national-level. Notably, floods, high winds (including hurricanes), and ice storms are excluded from this analysis, all of which are the most easily observable impacts to the electric grid, particularly the distribution system. These stressors are not included because even current state-of-the-art climate models represent these phenomena poorly due primarily to the coarse spatial scale of GCMs, as well as the complexity of representing extreme events such as hurricanes (where high winds and flooding cause major damage and power interruptions), convective storms (high winds), and ice storms. More detail on why these are excluded and potential changes in these stressors across the CONUS are provided in the Supplementary Materials (S.1, S.2 and S.3). Obviously this is a sizable limitation of the analysis and excluding these types of damages likely changes the total climate change costs in a substantial way. However, without a clear direction as to how these phenomena change in the future, it is impossible to include these costs. These are identified as important areas for future research.

Table 1 shows the different stressor-response relationships considered in this study: the green cells indicate those relationships that are included in the analysis, as well as the physical impact assessed (i.e. repair/replacement (R), lifespan reduction (L) and/or capacity change (C)), while the white and grey cells denote those excluded from the analysis either because the costs are insignificant (i) or too uncertain to estimate (u).

For ease of referring between this table and the results presented in Section 3, each of the stressor-response relationships included in this analysis (i.e. each green cell in Table 1) is shown in Table 2, where the first column corresponds to the number in Table 1. For ease of reference, the “Section” column lists the section where these are described in this paper and the “Supplement” column lists the section in the Supplemental Material with additional detail.

These ten different physical infrastructure impacts are then translated to economic impacts by estimating the costs associated with identified damages (Step 3). Costs can be associated with the occurrence of interruptions, repair, replacement, operation and maintenance or loss of capacity. Section 2.3 discusses the process of cost estimation in more detail.

Utilities and policy-makers will respond to these physical and economic impacts in a variety of ways, which may dampen or exacerbate the impacts. Here, we do not predict or estimate their response. Instead, we examine three basic responses or adaptation scenarios to compare impacts given varying adaptation responses (Step 4). We use the term “adaptation” to mean any measure taken to lower the risks posed by changes in climate – as opposed to “mitigation,” which is typically used in climate change science to refer to measures to reduce the amount and speed of future climate change by reducing emissions of GHGs.

The adaptation options considered are:

1. No Adaptation: Utilities and policy-makers assume a stationary climate and continue to design to current observed climate. These impacts represent an upper bound of possible future impacts for the stressors included in this analysis.
2. Reactive Adaptation: Utilities and policy-makers undertake adaptation once the damages or costs have been fully realized – and therefore react by upgrading design criteria.
3. Proactive Adaptation: Utilities and policy-makers proactively consider the risks of climate change and are willing to pay the upfront costs of adaptation before any damages occur.

These adaptation scenarios are not meant to be probable scenarios i.e., it is unlikely that utilities and policy-makers will continue in business-*as*-usual through the end of the century as is depicted in the No Adaptation scenario. However, when replacing either damaged or retired infrastructure, utilities often replace “like with like” to avoid the need to redesign or re-permit. One of the key recommendations from the American Society of Civil Engineers’ Infrastructure Report Card is to streamline permitting, in part for this reason [20]. Section 2.4 describes further details of these adaptation scenarios.

### 2.1. Inputs

This section describes the inputs needed for the analysis, including climate information and an infrastructure inventory.

#### 2.1.1. Climate-driven stressors

Climate-driven stressor information is input into our model in the form of one historical climate dataset, five future projections and two GHG emissions scenarios. These scenarios are consistent with those used in the second modelling phase of the Climate Change Impacts and Risk Analysis (CIRA2.0) project [21]. The future climate projections are a subset of those generated for the Intergovernmental Panel on Climate Change’s Fifth Assessment Report (AR5), namely CanESM2, CCSM4, GISS-E2-R, HadGEM2-ES and MIROC5. For climate forcing, two Representative Concentration Pathways (RCPs) are used: RCP8.5 and RCP4.5. RCP8.5 represents a future with substantial warming caused by higher GHG emissions, resulting in a total change in radiative forcing of 8.5 W/m^2^ by 2100 (compared to 1750). RCP4.5 represents a future with significant global reductions in GHG emissions, achieving a total radiative forcing of 4.5 W/m^2^ by 2100. These projections were downscaled using a statistically-based process that employs a multi-scale spatial matching scheme [22] at a spatial resolution of 1/16°. Further details of the projections, scenarios and the downscaling method are described in Section S.4.

The climate-driven stressors used in this study are temperature, precipitation, lightning, wildfires and vegetation growth. These stressors are either obtained directly from the downscaled climate parameters from the climate projections (i.e. temperature and precipitation) or derived from these climate parameters using the methods described below (i.e. for lightning, wildfires, vegetation growth and sea level rise and storm surge).

Lightning strike rates are estimated based on methods developed by Romps et al. [23]; using the product of convective available potential energy (CAPE) and precipitation (P) as a local proxy for strike rate. The constant of proportionality relating the lightning strike rate to CAPE x P was found by comparing each model’s average CAPE x P over the continental U.S. to the observed lightning strike rates during a specified historical period.

In order to analyze the impact of wildfires, we utilize area burned projections for the GCMs and RCPs at a half degree resolution across the Western U.S. This dataset of area burned was generated as part of forthcoming research on the impacts of wildfires on air pollution-related health outcomes in the Western U.S. The total area burned for each half degree grid cell was generated by adapting the process outlined in Yue et al. [24] to Localized Constructed Analogs (LOCA) temperature and precipitation estimates.

Vegetation, especially trees, poses significant threats to both transmission and distribution systems. Falling trees and limbs during extreme climatic events (hurricanes, wind storms, high moisture content snowfall, and ice storms) are generally more of a concern for the distribution system than primary failure of the structures themselves [2,25]. Aside from extreme events, vegetation that comes in contact with or too close to bare overhead cables can result in the connection of two lines or electric arcs which may result in fires [26]. For these reasons, utilities are required to regularly trim vegetation to a safe distance and have budget set aside to do so.

Climate change is likely to change vegetation growth due to changes in the growing season, precipitation, and CO_2_ fertilization. Although both longer growing seasons and increased CO_2_ fertilization will likely increase vegetation growth, reduced precipitation in certain regions of the CONUS could decrease growth. To assess these changes in vegetation, we use output from the MC2 dynamic global vegetation model developed and run by the U.S. Forest Service’s Pacific Northwest Research Station [27]. The model simulates growth and shifts in vegetation as influenced by climate (precipitation, temperature, humidity) as well as burned areas from wildfires (see Ref. [21] for additional methodological details).

For regional rates of relative sea level change, RCP-specific projections are applied using the approach described in NOAA [28]. To incorporate the effects of extreme water levels, this study uses storm surge probabilities derived from tide gauge data [28].

#### 2.1.2. Infrastructure inventory

While individual utilities keep records of their infrastructure inventory, detailed information on electricity infrastructure in the U.S. is not publicly available. As a result, we construct a CONUS-wide infrastructure inventory based on available information for population, electricity demand, and other variables and assumptions, combined with detailed information from specific utilities. To compile the baseline infrastructure inventory for 2015, we utilized a variety of data sources from government databases, companies in the electric infrastructure market, and other reports on the electricity grid. This information includes replacement costs for each type of infrastructure and varies by attributes (e.g., line voltage). Section S.5 provides further details.

As the economy grows and populations shift across the CONUS over time, the electricity grid will change accordingly. It is important to capture key features of a changing grid in the analysis in order to be able to adequately estimate future climate change impacts. Note that it is possible that in some places the transmission and distribution grid may contract in size due to the deployment of distributed energy resources (e.g., rooftop solar and batteries) but this is not captured in this study.

**Fig. 2.**
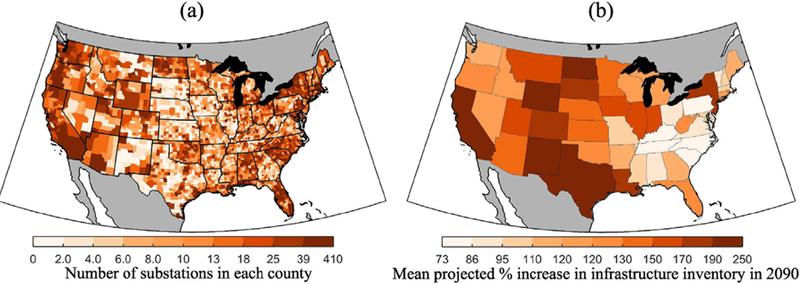
(a) Number of substations at the county-level and (b) average increase in infrastructure inventory, (in % increase) in 2090 as compared to 2015 for RCP 8.5, mean of all five GCMs.

In order to project the infrastructure inventory from 2015 through 2099, we assume a one-to-one relationship between state-level electricity demand growth and electric infrastructure growth. We utilize state-level electricity demand projections from the U.S. version of the Global Change Assessment Model (GCAM) for each GCM and RCP combination. These projections account for changes in demand in response to changes in temperature, i.e., changes in heating and air-conditioning electricity needs. The demand results of this model are described further in EPA [21]; while the methods used can be found in McFarland et al. [29]. Under a constant average temperature scenario (baseline climate), demand is projected to increase 85% nationally by 2099. Since demand responds to changes in temperature, demand projections are different across GCMs and RCPs. These temperature effects further increase national demand to 89% on average (from 87 to 91%) for RCP 4.5 and 96% on average (from 94% to 100%) for RCP 8.5 by 2099. We utilized population data by county for 2010 from the EPA’s Integrated Climate and Land-Use Scenarios (ICLUSv2) to allocate any distribution system infrastructure only available at the state-level to the counties within the state [21]. For illustration, Fig. 2 shows the number of substations in each county, obtained from HIFLD [30], and the mean state-level inventory projection, as % increase compared to 2015, for RCP 8.5.

### 2.2. Stressor-response relationships

This section describes the components of the stressor-response functions, for the different infrastructure components of the U.S. electricity transmission and distribution system.

#### 2.2.1. Transmission and distribution power lines

Rising ambient air temperature increases the resistance of conductors and thereby decreases the carrying capacity of cables. In combination with increasing demands from additional air conditioning usage, these decreases in capacity may create a bottleneck in the grid if extremely hot days become more common. We estimate decreases in line capacity applying the method used in Bartos et al. [3].

Direct lightning strikes on distribution lines are rare due to surrounding trees, buildings, or other structures, which are generally taller than distribution poles and lines. However, distribution lines are often impacted by indirect strikes which cause failure due to overvoltage. While transmission lines are well-protected against strikes, direct strikes do remain a concern. Repeated strikes may necessitate replacement of lightning protection infrastructure or other responses, which can be costly, although repair costs for both direct and indirect strikes on lines are often negligible. For this reason, repair costs are excluded from the analysis, but indirect impacts are captured in the form of power interruption costs to consumers.

Wildfires can also have impacts on power lines. Larger transmission lines held in place by steel lattice towers are not usually significantly impacted by wildfires, but lower voltage lines held in place by wooden poles can be. However, these lines are often near buildings or residential areas and may be protected by fire-fighting efforts. For this reason, we do not model wildfire impacts on distribution lines. Transmission lines are impacted by heat caused by wildfires, leakage currents due to ionized air in smoke, and deposition of soot on insulators. Redundancy in the transmission network minimizes interruption-related impacts of wildfires and efficiency losses due to ionized air in smoke are typically not significant due to the infrequent occurrence of wildfires. This study therefore only considers the destruction of low-lying transmission lines by wildfires, and uses an average repair cost of transmission lines per mile [31] to quantify the impact of wildfires on transmission infrastructure.

#### 2.2.2. Structures

Transmission towers are typically large steel lattice structures that are expensive and are essential to the grid since they carry conductors that transfer large amounts of power. For this reason, they are designed to withstand extreme weather with high safety factors and built in parallel or networked systems. While there are cases where transmission towers have fallen due to ice or wind, these are uncommon - these lattice structures are rarely the cause of interruptions [32]. Hence, we do not consider interruptions or repair costs from transmission tower structural failure in this study. Wooden transmission structures are also used. However, we could not find information that distinguishes lattice towers from wooden structures on a national scale, so this distinction was not considered.

Wood structures, especially timber poles, are numerous in the distribution system and structural failures driven by extreme wind speeds, ice storms, falling trees or heavy limbs regularly cause interruptions. As previously shown in Table 1, the primary stressors impacting wood poles in this study are air temperature, precipitation and tree limbs. The primary mechanism by which air temperature and precipitation impact wood poles is through degradation from fungal attack at the base. We estimate timber pole degradation using the method of Wang et al. [33]; used in Wang and Wang [34] and Bjarnadottir et al. [35]. The general form of the relationship between climate and pole decay rate (caused by fungal attack) is as follows.

$$r=k_{w}f{\left(R\right)}^{0.3}g{\left(T\right)}^{0.2}$$

where r is decay rate, kw is a wood-specific parameter, f(R) is a function that includes rainfall, R, and g(T) is a function that includes temperature, T.

Decay reduces the diameter of the pole, which then reduces the pole strength. The strength of the pole is estimated as shown in Wang et al. [33].

#### 2.2.3. Substations and distribution transformers

Substations are complex structures that convert voltage. They involve many parts, and any impacts to these parts will impact the substation function itself. We consider two types of transformers: large power transformers within substations (herein referred to as “substation transformers”); and standalone transformers, either on the ground in a covered metal box or fixed on power poles (herein referred to as “distribution transformers”).

Within this study, transformers are modelled as being primarily impacted by changing ambient air temperature (impacting transformer lifespan and capacity) and sea level rise/storm surge. Complete transformer failure due to high temperatures or lightning strikes is rare and poorly characterized historically, and are thus excluded from this analysis (see Section S.10 and S.11 respectively for details).

Air temperature impacts transformer peak load capacity such that higher temperatures decrease capacity. A number of studies have quantified the general relationship between air temperature and transformer capacity (e.g., Refs. [36,37]). One method for assessing these impacts is to assume a linear relationship between ambient air temperature and load capacity, as done in Sathaye et al. [19]. We apply this method to relate temperature to the capacity of substation transformers.

Ambient air temperature also impacts transformer lifespan. Transformers are typically cooled with oil-based convective heat sinks. These cooling systems sometimes develop “hot spots,” which can damage the insulating paper that prevent short circuits. Warmer operating temperatures reduce the expected life of transformers as this insulating paper ages faster. The methods described by Lundgaard et al. [38] and Stahlhut et al. [39] are used to estimate the impact of increasing temperature on the expected lifespan of substation transformers and distribution transformers, respectively. Both methods follow a similar form. For example for distribution transformers, the loss of life (LOL) is estimated as

$$LOL=100t\left(10^{-\left[A+\frac{B}{T}\right]}\right)$$

where t is the time-step at constant load which is calculated at the hourly level; A and B are parameters specific to the transformer and temperature, T, is estimated using a simple thermal model.

$$T=273+\theta_{a}+\theta_{o}+\theta_{g}$$

where θa is the ambient temperature, θo is the is the top-oil rise over ambient temperature, and θg is the hottest spot conductor rise over top-oil temperature [39].

Although sea level rise and storm surge are likely to have impacts to poles, lines, and distribution transformers, our analysis is limited to the impacts to substations because we can estimate substation elevations and locations along the coast. These impacts are based on results from the National Coastal Property Model (NCPM; [40]. The model was modified to include substations using the flood damage curves developed for the Federal Emergency Management Agency (FEMA)’s Hazus model to estimate storm surge damage.

#### 2.2.4. Vegetation management

The impact of increased vegetation on the U.S. electricity transmission and distribution system is quantified simply. We assume that with increased vegetation, vegetation management costs will also increase and that this relationship is linear. However, we recognize that in areas of the country with more tree cover, vegetation management costs are higher than in regions that are drier (e.g., Arizona). Using the vegetation management budget for multiple utilities (PG&E, Jersey Central Power and Light, and Northeast Utilities), we find that costs per line mile per tree density (measured as a deviation from the national mean) are relatively similar across utilities [41–43].

### 2.3. Economic impacts

The costs of physical damages are estimated using an engineering cost analysis. This involves calculating the net present value of repair or replacement costs to infrastructure, changes to operation and maintenance costs and interruption costs. All costs are translated into 2017 US dollars.

Replacement costs (after failure or at the end of the infrastructure’s lifespan) are estimated based on the cost of design and construction of new infrastructure. This does assume that system design is not changed when a piece of infrastructure is replaced-estimating the cost of changes in system design is complicated and beyond the scope of this study. Making the assumption that infrastructure is replaced as needed, we apply the cost estimation method used in Larsen et al. [18].

We recognize that climate change is likely to increase power interruptions, causing significant costs to consumers [17]. Although this is not the focus of this study, we completed back-of-the-envelope estimates of these interruption costs using the Interruption Cost Estimator (ICE) model described in Refs. [44,45]—a tool commonly used by both utilities and regulators (see Campbell [2] for instance). Section S.14 provides further details.

### 2.4. Adaptation measures

The response of utilities and decision makers to the various climate impacts is represented in the analysis using a straightforward approach.

For the **No Adaptation strategy**, utilities make no adjustments to infrastructure design, treating climate as if it has remained stationary.

For the **Reactive and Proactive Adaptation strategies**, utilities respond by “designing” infrastructure to either the past (reactive) or projected future (proactive) climate. This is done by upgrading infrastructure until it meets the baseline performance or service level. For example, for wood pole lifespan reductions, upgrades are made until the historic baseline lifespan is once again reached. Reactive and Proactive Adaptation are distinguished by designing to different climates, either reactive to the past climate or proactive to a projected future climate.

The Reactive Adaptation strategy sees utilities respond only to those climate impacts that have been fully realized. For example, for changes to wood pole lifespan, utilities will design taking into account wood pole retirements that have occurred earlier than expected. The subsequent response by utilities is to design based on the climate over the lifespan of these same retired power poles by upgrading a portion of the replaced poles until the average lifespan is equal to the pre-climate change lifespan in that county.

Proactive Adaptation is similar, in that all new infrastructure built in the current year is designed to the climate over the infrastructure’s lifespan; however, Proactive Adaptation designs to the future lifespan using a projected climate. We do not use perfect foresight here, which assumes the future is certain. Instead, we establish a projected change in climate using the mean over the different GCMs. In this way, Proactive Adaptation is forward-looking but does not design perfectly. In some cases, the infrastructure is overdesigned and in others it is under-designed, always slightly underperforming a “perfect” design that would have been selected under perfect foresight.

More detail on these adaptation measures is provided in S.15. In brief, adaptation responses are as follows: to reductions in transformer lifespan, build additional capacity to reduce the load on each transformer; to wood pole lifespan reductions, reinforce with steel or concrete; to reductions in power line capacity, use a higher ampacity cable; to sea level rise and storm surge, build a protective sea wall. Note that for wildfire repair and vegetation management, we do not evaluate specific adaptation options. For vegetation management, which is itself a response to climate-driven changes, advances in outage prediction [46,47] may reduce these costs by directing utilities toward trees and lines most vulnerable. However, this effect is not captured in this analysis.

## 3. Results

### 3.1. Results by infrastructure-impact category

**Fig. 3.**
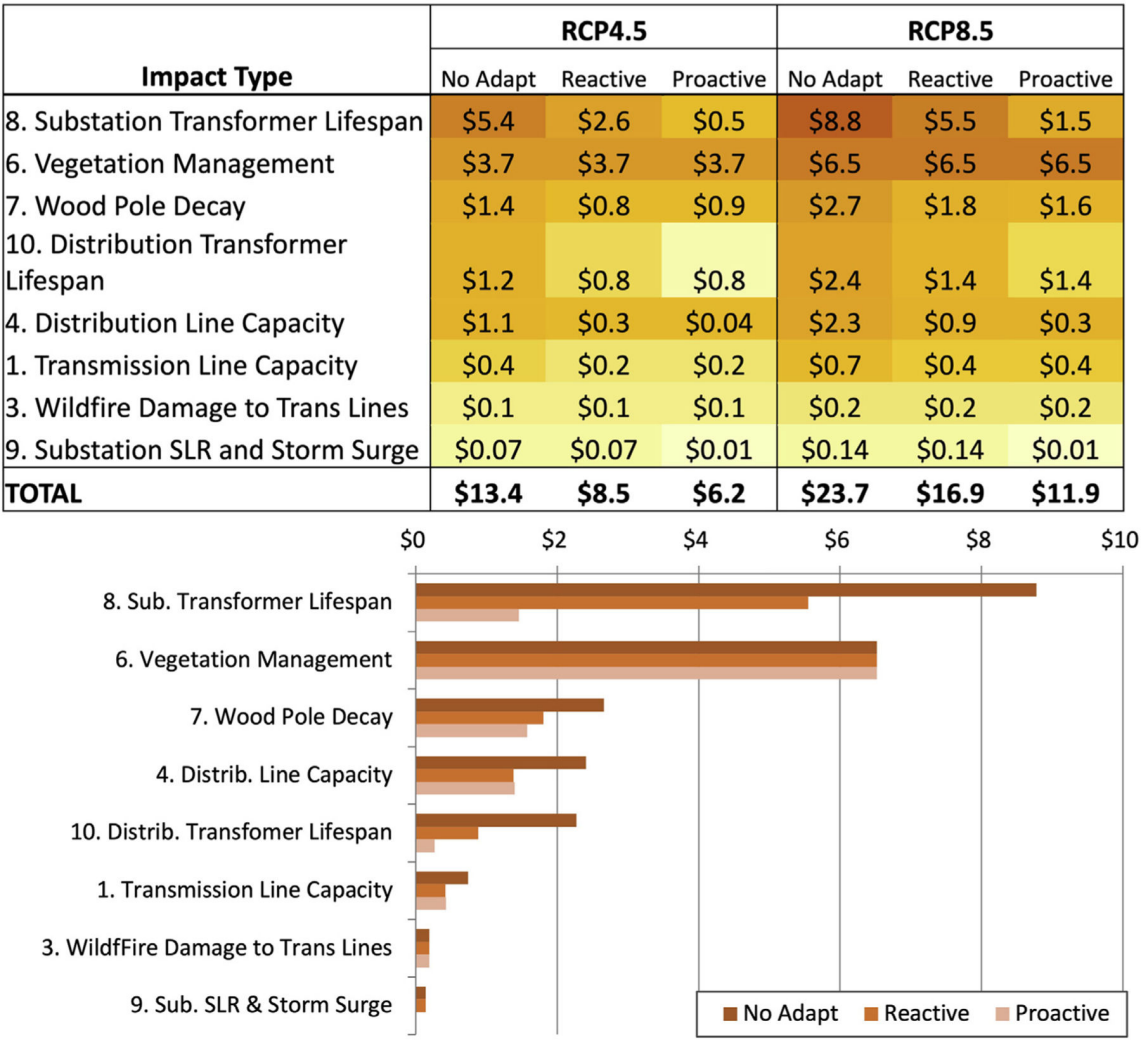
The upper panel shows the annual average climate change costs (billions $2017/year) projected during the 2080–2099 period under the two emissions scenarios, three adaptation strategies and nine impact categories, averaged across the five GCMs. These totals include adaptation costs for the Reactive and Proactive Adaptation strategies. The lower panel displays the RCP8.5 emission results graphically. Results are organized from highest to lowest cost impact category. The numbering of each impact category corresponds to the numbering introduced in Table 1. Only eight of ten impact categories are shown here as impact category #2: Lightning on transmission lines and #5: Lightning on distribution lines are not associated with infrastructure damages, just interruption costs, as discussed in Section 4.

This section presents insights into how the modelled climate change impacts differ across adaptation strategies and emissions scenarios, and vary across the stressor-response categories included in this analysis (previously identified in Table 1). Broadly, we find that climate change reduces grid infrastructure performance and/or reliability. As shown on Fig. 3, total annual economic climate change impacts (with costs of adaptation) for all stressor-response categories at the end of the century range from $6 to $24 billion, depending on emission scenario and adaptation strategy. These costs include the costs of adaptation and are the change in costs compared to a no climate change scenario—i.e., with infrastructure growth and baseline climate. For reference, without climate change, total annual expenditures for these infrastructure types would be about $95 billion/year in 2090, so an increase of $24 billion is about 25% higher. Annual impacts under the more extreme RCP8.5 scenario are roughly double those experienced under the lower emissions RCP4.5 scenario due to the much higher end-of-century temperature projections associated with RCP8.5. Moving from a strategy of No Adaptation to a Reactive or Proactive Adaptation strategy also roughly halves the expected costs of climate change experienced in 2090, with the proactive strategy providing larger reductions.

Looking more closely at the annual costs associated with each of the different stressor-response categories (Fig. 3), reductions in the lifespan of substation transformers and increases in vegetation management expenditures represent the most costly impact categories, accounting for roughly 65% of costs under the No Adaptation strategy, under both emissions scenarios. These two impact categories are revisited in more detail in Section 3.3. The costs associated with the remaining stressor-response categories are less significant at the national scale, but can have a more pronounced effect regionally, as is found to be the case for wildfire damage and impacts to substations due to sea level rise and storm surge.

### 3.2. Evolution of infrastructure impacts over time

**Fig. 4.**
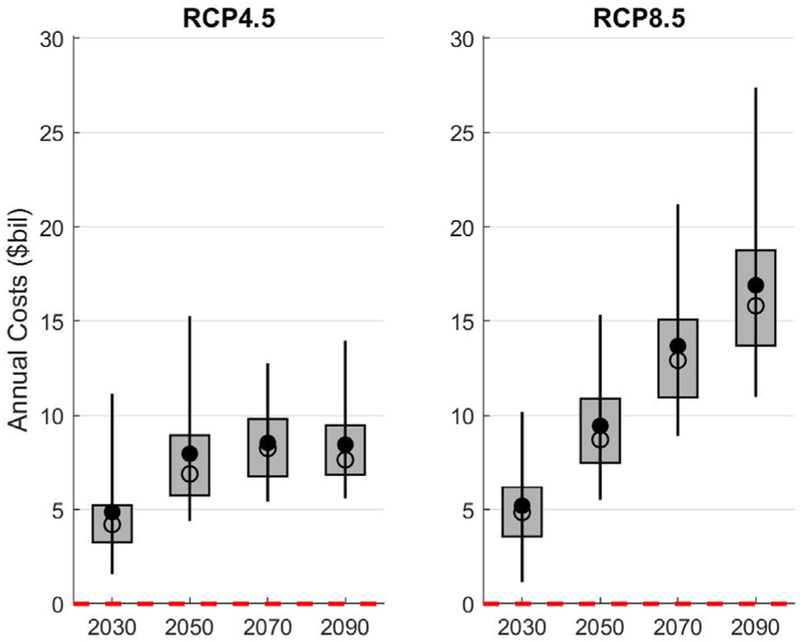
Change in annual costs (billions $2017/year) across the five GCMs for 2030, 2050, 2070, and 2090, under each of the climate emissions scenarios for the Reactive Adaptation strategy. Each boxplot contains 100 points made up of the five GCMs and 20 years within each era. The whiskers represent the 5th to 95th percentiles of these data, the boxes capture the 25th to 75th percentiles, and the filled and open circles are the mean and median across the data, respectively.

This section presents insights into how total CONUS-level impact costs evolve over time for the Reactive Adaptation strategy, as well as the uncertainty across the five GCMs used. Fig. 4 demonstrates that, as expected, the divergence between the two emissions scenarios and the five GCMs becomes increasingly more significant after 2050. The GCMs associated with the highest infrastructure impacts in 2090 indicate costs that are $11 billion/year higher than those GCMs associated with median impacts in 2090. Note that under the Proactive Adaptation strategy, this high-median difference decreases to only $6 billion/year, highlighting the importance of timely adaptation for reducing the magnitude as well as the variability of infrastructure impacts.

### 3.3. Spatial findings for the most costly infrastructure-impact categories

**Fig. 5.**
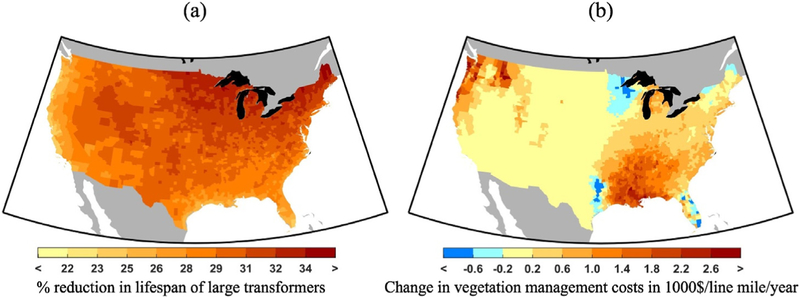
(a) Reduction in lifespan of large transformers (%) and (b) change in vegetation management costs ($ thousand/line mile/year) in 2090 as compared to the baseline for RCP 8.5, mean of all five GCMs.

Section 3.1 noted that reductions in substation transformer lifespan and increases in vegetation management costs represent the two most costly impact categories of the ten categories examined. This section revisits these two impacts, looking in more detail at their spatial distributions across the CONUS. Fig. 5a shows how the reduction in large substation transformer lifespan is highest in the Midwest and Northeast and lowest along the Gulf Coast and Western coast, as driven by higher and lower changes in temperature respectively. Fig. 5b displays how vegetation management costs increase overall in the Northwestern U.S. and in the East of the country, especially along the Gulf Coast, in response to extended growing seasons and/or precipitation levels. Conversely, costs decrease in parts of Florida, the Midwest and parts of Texas due to reductions in precipitation. Most of the West, excluding coastal and river waterways, where tree cover is low, shows little to no projected change in vegetation management costs.

### 3.4. Spatial findings for the ratio of annual impact costs to annual electricity sales

**Fig. 6.**
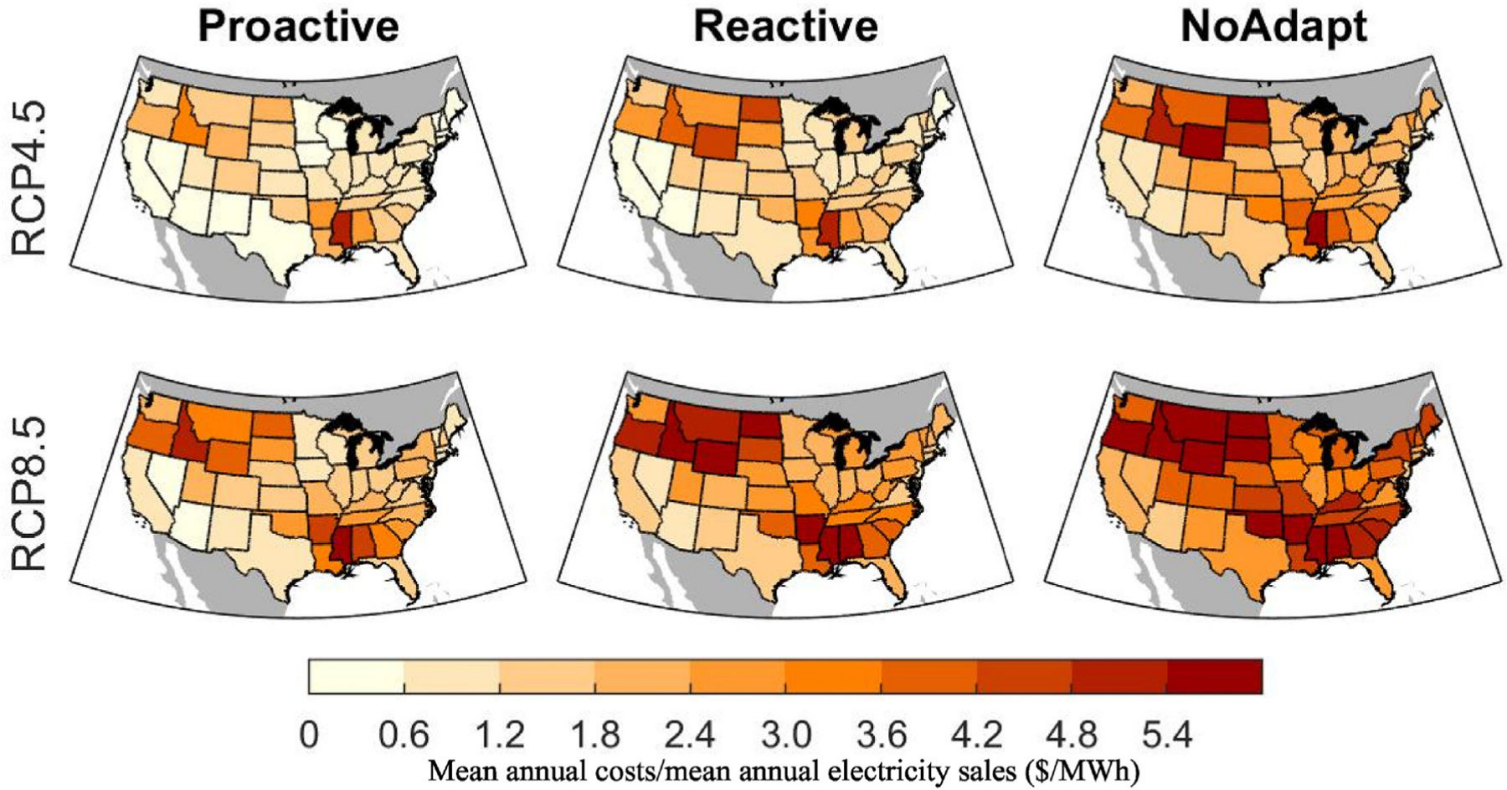
The ratio of mean annual costs to mean annual electricity sales (units are $/MWh) projected during the 2080–2099 period across the two emissions scenarios and three adaptation strategies, averaged across the five GCMs. Data aggregated from the county to state level.

This section explores how the ratio of mean annual impact costs to mean annual electricity sales varies across the CONUS. This ratio gives an indication of the degree to which electricity costs could rise in the future if these impact costs are passed on to electricity consumers. Fig. 6 shows total annual end-of-century costs divided by electricity sales, with impacts varying from less than $0.5/MWh to upwards of $6/MWh. Although the large costs associated with impacts to large substation transformer lifespan are fairly constant across the country, the spatial pattern of changes in vegetation management show up as high $/MWh costs in the Northwest and Southeast, where vegetation management costs increase substantially. The high impacts in the central northern region of the country are driven by a relatively high ratio of grid infrastructure to electricity sales in those regions. It is unclear how much of these costs might be absorbed by the utility versus passed along to ratepayers.

### 3.5. Net present value of total climate change impacts from 2018 to 2099

**Fig. 7.**
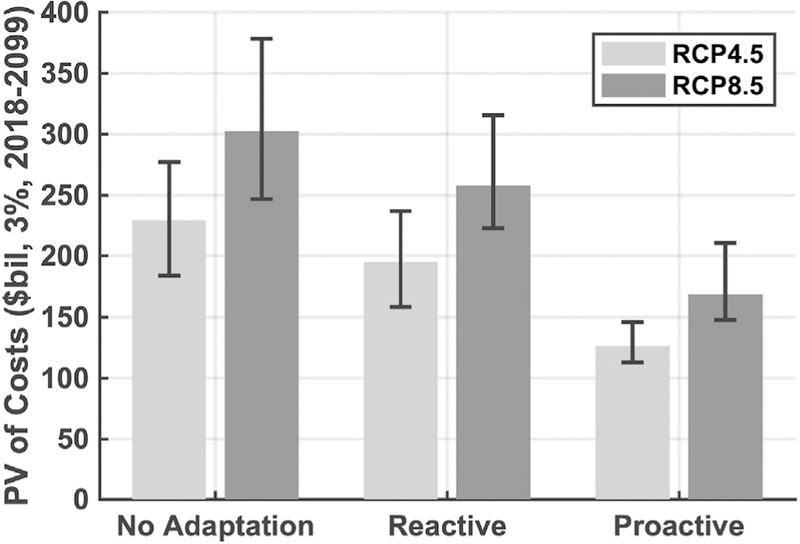
Net present value ($2017) of total CONUS-level costs, 2018e2099, discounted at 3%. Black line indicates the range of impacts across the five GCMs.

The net present value of total costs of climate change impacts across GCMs, emission scenarios, and adaptation strategies, ranges from $120 to $380 billion through 2099 (Fig. 7), when using a discount rate of 3%. The largest climate impacts occur after 2050, and these impacts are also the most heavily discounted. The effects of substantial discounting limit the difference between the RCP4.5 and RCP8.5 emissions scenarios to about 20%, as compared to 50% cost differences seen in the undiscounted, end-of-century annual costs presented in Fig. 3. Looking across the different adaptation strategies, adaptation efforts do still have a profound effect on net present costs. Within a given emissions scenario, moving from a No Adaptation to a Proactive Adaptation strategy reduces net present costs by as much as 50%.

## 4. Discussion

In this study, we explored the costs of climate change on the U.S. electricity transmission and distribution system. We did this by developing and running a screening model built around a series of stressor-response functions that relate the physical effects of climate change on the various components of the transmission and distribution network of the CONUS at the county-level. This study focused on identifying climate-attributable changes in the performance and longevity of physical infrastructure, such as power poles and transformers, and quantifying these impacts in economic terms. This focus on infrastructure is crucial to note, as it does not include consideration of the costs of power interruptions, which have been shown to be substantial [6,17]. This omission of any customer costs associated with power interruptions is a critical area of future research that is discussed in Section 4.3. Furthermore, this work focuses on a subset of ten stressor-response infrastructure interactions (Table 1) that are both particularly climate-sensitive and amenable to adequate representation within a screening level analysis. Thus, all of the results presented capture only a portion of the true range of climate change impacts to the grid. Even so, the total electricity transmission and distribution infrastructure costs across the CONUS estimated in this study rise considerably under climate change. Given that the American Society of Civil Engineers already rates the U.S. electrical grid a D+ [20], such climate impacts will only intensify threats to an already vulnerable infrastructure system.

### 4.1. Spatial dimensions of climate change impacts

Many climate impacts, such as river runoff or extreme weather events, are associated with a large degree of spatial heterogeneity, with impacts typically varying widely across a study area. Impacts to the grid system, however, are largely driven by projected increases in temperature, which occur across the entire CONUS. Consequently, our results demonstrate that nowhere in the CONUS is immune from impacts to grid infrastructure.

That said, increasing temperature is not the only climate stressor that affects the U.S. electricity transmission and distribution system. Other stressors like sea level rise, storm surge, wood pole lifespan, and altered vegetation growth are more spatially heterogeneous and do result in varied impacts across the CONUS. For instance, substantial costs for increased vegetation management are projected in the Southeast and Northwest. This spatial dimension raises questions of equity and inclusiveness given that many of the lower income states in the U.S. are expected to be most affected by impacts to their electric power infrastructure.

### 4.2. Adaptation dimensions of climate change impacts

Although this is not an adaptation-focused analysis to inform utility-level planning, the study nonetheless quantifies costs under three different adaptation strategies to explore the inherent uncertainty in how utilities may respond to climate change impacts. A comparison of total impact costs between the No Adaptation, Reactive and Proactive Adaptation strategies reveal that a large fraction of the impacts of climate change can be addressed through planned adaptation measures that are well-timed and correctly implemented. While the benefit of moving from a strategy of Reactive Adaptation to one of Proactive Adaptation strategy is relatively smaller—due in part to the uncertainty in projecting climate over the life of long-lived infrastructure—the evidence does suggest there are additional benefits to approaching adaptation in a more proactive way.

Such adaptation measures are particularly important when considering the long-lived nature of these infrastructure elements. Large temperature changes projected after 2050 mean infrastructure built today needs to be designed in such a way as to remain functional under a much different climatic future than we experience at present. While the No Adaptation strategy is somewhat unlikely, in that utilities and policy-makers are expected to adapt their business-*as*-usual state as they begin to experience climate change impacts, it does offer insights about what utilities could face if the usual practice of replacing like-with-like is continued.

### 4.3. Study limitations and areas for future research

Like all studies examining climate change impacts, the work presented here was limited by the resolution and confidence levels of simulated climate data obtained from existing GCMs. Additionally, given the diverse sources of uncertainty involved in this study, further analysis and validation of the results would be useful.

There are also limitations with respect to the stressor-response relationships included in this work. As this study was developed as a screening level analysis, there is much room for improving upon these stressor-response relationships. Furthermore, as described in Section 2, certain key stressor-response relationships, such as floods, high winds and ice storms, were not included in this study because even state-of-the-art models cannot currently represent these phenomena adequately at the scale necessary for this type of analysis. Thus, as improved projections of extreme weather events become available, additional stressor-response functions should be developed that allow them to be incorporated in studies such as these.

As noted earlier, this study does not focus on changes in the frequency, duration and scale of electric power interruptions under climate change but looks primarily at infrastructure damage impacts. Revisiting this question of power interruptions is an important area of inquiry, as current estimates for weather-related interruption costs total between $25 to $70 billion per year [2]; although the range of total interruption costs varies substantially from $28 to $209 billion per year [2,48,49]. These costs are likely to increase further under climate change. An initial CONUS-wide study on this topic has been conducted by Larsen et al. [17]; who developed an econometric model with inputs from the Interruption Cost Estimate (ICE) calculator. In the context of this current work, making several simplifying assumptions and drawing on interruption unit costs from the ICE calculator [45], the total costs of power interruptions for the stressors modelled here could be ~$4 to $7 billion per year by 2090 (see S.14). However, the ICE calculator does not consider the direct costs to customers and the indirect impacts to the economy from long-duration, widespread power interruptions. Further research in this area is critical given that this is likely a significant underestimate of the true costs of power interruptions under climate change.

## 5. Conclusions

This study presented the results of developing a screening-level model to estimate the infrastructure-related costs of climate change on the U.S. electricity transmission and distribution system. The screening model utilized climate and infrastructure inputs to quantify physical damages to the existing infrastructure inventory using climate-driven stressor-response functions for different climate models and emission scenarios. The economic impacts of these physical damages were subsequently computed, before assessing the performance of different adaptation strategies.

The analysis showed that broadly speaking, future climate change is expected to reduce grid infrastructure performance and, in some cases, reliability. Total electricity infrastructure costs across the CONUS are projected to rise considerably under climate change, with annual costs increasing by as much as a quarter. These results captured only a portion of the true impacts to the grid, as this analysis only included a fraction of the relevant stressor-response infrastructure interactions. These results demonstrate that climate change has the potential to dramatically intensify current weather-related impacts to the U.S. power infrastructure. Among all the different infrastructure impacts examined, reductions in the lifespan of substation transformers and increases in vegetation management expenditures were the most costly impact categories. This first impact category is largely driven by projected increases in air temperature, meaning that no location in the CONUS is immune. Conversely, changes in vegetation growth are more spatially heterogeneous, resulting in more varied impacts across the CONUS. The differing spatial nature of impacts offers important insights into the need to regionally tailor possible policy and adaptation responses.

The results and insights presented here demonstrate that climate impacts will likely be substantial and that several improvements and extensions to this analysis should be undertaken, extending this work to be more directly applicable to climate resilient utility-level planning for the future.

## Supplementary Material

Supplement 1

## Figures and Tables

**Table 1:** Stressor-response relationships considered.

Infrastructure	Air Temperature	Rain	Lightning	Veg. Management	Wildfires	SLR & Storm Surge	Floods	High Winds	Ice Storms
**Transmission Lines**	C (#1)	i	R* (#2)	i	R (#3)	i	i	i	i
**Distribution Lines**	C (#4)	i	R* (#5)	R (#6)	u	u	u	u	u
**Transmission Towers**	i	i	i	i	i	i	i	i	i
**Wood Poles**	L (#7)		u	R (#6)	u	u	u	u	u
**Substations / Large Transformers**	L, C (#8)	i	i	I	u	R (#9)	u	u	u
**Distribution Transformers**	L,C (#10)	i	u	R (#6)	u	u	u	u	u

**Key** Repair/Replacement/Interruption (R), Lifespan Reduction (L), Capacity Change (C), insignificant costs (i), significant uncertainty in costs (u).

Green indicates relationships is included in the analysis, white and grey indicate relationship is not included, and grey further indicates significant uncertainty in the underlying climate stressor.

Numbers in brackets correspond to the numbering of the summary list following the table.

* These stressor-responses not associated with infrastructure damages, just interruption costs, as discussed in Section 4.

**Table 2:** Detail and reference of stressor-response relationships.

#	Name	Description	Section	Supplement
**1**	Transmission Line Capacity	High temperatures on transmission lines cause a reduction in ampacity	2.2.1	S.6
**2**	Lightning on Transmission Lines	Line failures caused by direct lightning strikes are included in the form of interruption costs	2.2.1	S.7
**3**	Wildfire Damage to Transmission Lines	Heat from wildfires causes damage to transmission lines, which requires repair	2.2.1	S.8
**4**	Distribution Line Capacity	High temperatures on distribution lines cause a reduction in ampacity	2.2.1	S.6
**5**	Lightning on Distribution lines	Indirect lightning strikes cause failures on distribution lines, which are included in the form of interruption costs	2.2.1	S.7
**6**	Vegetation Management	Changes in climate result in altered vegetation growth, which requires changes in vegetation management	2.2.4	–
**7**	Wood Pole Decay	Changes in precipitation and temperature alter the rate of decay at the base of the wood poles	2.2.2	S.9
**8**	Substation (Large) Transformer Lifespan and Capacity	Changes in air temperature cause changes in lifespan or capacity of large power transformers	2.2.3	S.12
**9**	Substation Damage from Sea Level Rise and Storm Surge	Water damage or costs to relocate salvageable substations from either sea inundation or rising storm surge heights	2.2.3	S.13
**10**	Distribution Transformer Lifespan	Changes in air temperature alter the lifespan of distribution transformers	2.2.3	S.12

Note: First (#) column refers to the number in Table 1; the Section column refers to the section in this paper; and the Supplement column refers to the section in the Supplemental Material where more detail is provided.
