# Supplementary material for: Climate change impacts and costs to U.S. electricity transmission and distribution infrastructure
Source: Energy (Oxf). Author manuscript; Available in PMC 2020 Apr 15. (PMC7017388; doi:10.1016/j.energy.2020.116899)
Supplement: Supplement 1 [file NIHMS1551236-supplement-Supplement_1.docx]

**Climate Change Impacts and Associated Costs to U.S. Electricity Transmission and Distribution Infrastructure**

# SUPPLEMENTARY MATERIALS

Charles Fant^a*^, Brent Boehlert^a,b^, Kenneth Strzepek^b,a^, Peter Larsen^c^, Alisa White^a^, Sahil Gulati^a^, Yue Li^d^, Jeremy Martinich^e^

a. Industrial Economics, Inc., 2067 Massachusetts Ave, Cambridge, MA 02140, USA

b. Massachusetts Institute of Technology, 77 Massachusetts Ave, Cambridge, MA 02139, USA

c. Carroll College, 1601 N Benton Ave. Helena, MT 59625 , USA

d. Case Western Reserve University, Nord 500, Cleveland, Ohio 44106, USA

e. U.S. Environmental Protection Agency (EPA), Washington, D.C., USA

* Corresponding author, email: [cfant@indecon.com](mailto:cfant@indecon.com)

Contents

[SUPPLEMENTARY MATERIALS 1](#_Toc524702637)

[S.1 Climate Stressors - A Note on Inland Flooding 4](#_Toc524702638)

[S.2 Climate Stressors - A Note on High Winds 4](#_Toc524702639)

[S.3 Climate Stressors - A Note on Ice Storms 5](#_Toc524702640)

[S.4 Climate Stressors – Details on Scenarios 6](#_Toc524702641)

[S.5 Infrastructure Inventory 9](#_Toc524702644)

[S.6 Stressor-Response Relationships – Impact of Air Temperature on Power Lines 15](#_Toc524702646)

[S.7 Stressor-Response Relationships – Impact of Lightning on Power Lines 16](#_Toc524702647)

[S.8. Stressor-Response Relationships – Impact of Wildfires on Power Lines 19](#_Toc524702653)

[S.9 Stressor-Response Relationships – Timber Pole Degradation 21](#_Toc524702654)

[S.10 Stressor-Response Relationships – Impact of Temperature on Transformer Failure 22](#_Toc524702655)

[S.11 Stressor-Response Relationships – Impact of Direct Lightning Strike on Transformers 22](#_Toc524702656)

[S.12 Stressor-Response Relationships – Impact of Temperature on Transformer Lifespan 22](#_Toc524702657)

[S.13 Stressor-Response Relationships – Impact of Sea Level Rise and Storm Surge on Transformers 24](#_Toc524702658)

[S.14 Economic Impacts – Estimating Power Interruption Costs 24](#_Toc524702659)

[S.15 Adaptation Measures 27](#_Toc524702660)

[S.16 Maps of Infrastructure 29](#_Toc524702661)

[S.17 Maps of Climate-Driven Stressors 31](#_Toc524702662)

[S.18 Maps of Stressor-Response Results 36](#_Toc524702663)

[S.17 Map of Total Costs 49](#_Toc524702664)

## Description of all Data sources

Intermediate output, Final output, and code available here:

[**https://www.indecon.com/iec-climate-change-trans-dist-article/**](https://www.indecon.com/iec-climate-change-trans-dist-article/)

Table 1: Data sources and links of all data used but not generated by the authors

| **Name** | **Description** | **Source** |
| --- | --- | --- |
| Substations, Transmission Lines, and electric retail servies | Homeland Infrastructure Foundation Level Data; contains shapefile for substations, transmission lines, and electric retail service territories (ERSTs) across the U.S | https://hifld-geoplatform.opendata.arcgis.com/ |
| Vermont Distribution Infrastructure | The state of Vermont has a shapefile of all of the power poles overseen by Green Mountain power in the state | http://geodata.vermont.gov/ |
| FERC Form 1 | Federal Energy Regulatory Commission has annual reports on electric utilities across the United States; contains information on substation transformers | https://www.ferc.gov/docs-filing/forms/form-1/data.asp |
| Distribution line miles, distribution transformers | 2017 UDI Directory of Electric Power Producers and Distributors; used to calculate number of distribution line miles and distribution transformers per county | https://www.spglobal.com/platts/en/products-services/electric-power |
| on electricity sales and number of customers by state, utility, and sector | The Energy Information Administration has yearly data on electricity sales and number of customers by state, utility, and broken down by sector (industrial, residential, commercial) | https://www.eia.gov/electricity/data/eia861/ |
| Electricity demand projections | Global Climate Assessment Model (GCAM) demand projections by State, used to project the infrastructure | McFarland, J., Y. Zhou, L. Clarke, P. Sullivan, J. Colman, W. Jaglom, ... and J. Creason. 2015. Impacts of rising air temperatures and emissions mitigation on electricity demand and supply in the United States: a multi-model comparison. Climatic Change. DOI: 10.1007/s10584-015-1380-8. |
| Human population projections | Population data from the Integrated Climate and Land-Use Scenarios | https://www.epa.gov/iclus |
| Bias corrected precipitation and temperature changes | Localized Constructed Analogs contain temperature and precipitation data for a range of climate scenarios, baseline and projection years. | http://loca.ucsd.edu/ |
| Sea level rise and tide gauge levels | Sea level rise projections and tide gauge levels used to develop storm surge heights and probabilities | National Oceanographic and Atmospheric Administration. (2017). Global and regional sea level rise scenarios for the United States. NOAA Center for Operational Oceanographic Products and Services, Technical Report NOS CO-OPS 083. |
| Lightning Strike Projections | Change in lightning strikes across CONUS | Romps, D., Seeley, J., Vollaro, D. and Molinari, J. (2014). Projected Increase in Lightning Strikes in the United States Due to Global Warming. Science, 346(6211), 851-854. |
| Changes in above-ground tree carbon | Changes in above-ground tree carbon used to as a proxy to project changes in vegetation management costs | EPA. 2017. Multi-Model Framework for Quantitative Sectoral Impacts Analysis: A Technical Report for  the Fourth National Climate Assessment. U.S. Environmental Protection Agency, EPA 430-R-17-001. |
| Wildfires data | This data is not yet publicly available as it is being used for a yet unpublished manuscript. The data will also become available as the other manuscript is published. | public posting in progress |

## S.1 Climate Stressors - A Note on Inland Flooding

Flood events can destroy expensive infrastructure like substations and transformers. However, determining flooding inundation levels requires modeling flood accumulation at the spatial scale of the infrastructure itself. Also, flood plains are typically avoided when determining construction sites for expensive infrastructure like substations.

However, using a GIS analysis, we find that about 500 substations are within the FEMA 100-year floodplain, which represents about 0.5% of the total substations in CONUS. Wobus et al. (2017) finds that 100-year flood damages are likely to increase over the next century. However, as Sathaye et al. (2013) describe, locations of substations may not be accurate to the scale required to evaluate flood risks. The structures (i.e., towers and poles) used to hold overhead power lines are not typically damaged by flooding events, as wind is far more hazardous. However, underground cables can be vulnerable to floods.

## S.2 Climate Stressors - A Note on High Winds

High winds cause a significant amount of damage to distribution infrastructure. However, there is low confidence in the projection of near-surface wind speed changes from GCM output. The GCMs provide wind speed output at 10m elevation, an estimation derived from the wind speed values of the atmospheric layer closest to the surface. Vertical layers in a GCM are typically defined by constant pressure, meaning that the layer heights change in space and time. These pressure layers are also unevenly distributed so that a finer resolution is achieved near the surface. In a typical GCM, the atmosphere is modeled with about 10 – 20 layers reaching to about 30km above the Earth’s surface. GCMs also represent the climate at a coarse horizontal resolution of about 250 to 600 km (IPCC 2012). The problem with dividing the atmosphere into large cubes is that many processes occur at a much smaller scale. These large cubes are not ideal for modeling or resolving changes in small-scale wind, which is highly dependent on the effects of elevation, surface roughness, and convection. Topography also plays an essential role in wind speeds. Elevation in a GCM is flat within these large grids. Extreme wind speeds are especially uncertain due to the chaotic and turbulent state of the atmosphere when high winds occur. Hurricanes and convective storms, which are often the cause of distribution infrastructure failure, are not represented in GCMs and need to be approximated using exogenous modeling techniques, making use of GCM output (e.g., Emanuel et al. 2013).

For these reasons, impacts related to wind speeds are excluded from this analysis.

Others have estimated how mean wind speeds are likely to change in the U.S., usually related to power production from wind turbines. For example, Kulkarni and Huang (2014) find most of the U.S. is likely to experience increases in mean near-surface wind speeds of about 5-10 percent by the end of the century, using the Coupled Model Intercomparison Project – 5 (CMIP-5) GCMs. Chen et al. (2013) apply a complex downscaling technique to provide estimates of changes in wind speed gusts for 104 stations across Canada. They find hourly wind speed gusts are likely to increase 20-30% for most of these stations, using GCM output from CMIP-3.

## S.3 Climate Stressors - A Note on Ice Storms

Ice storms are a major concern for utilities. Between the years 1949 and 2003, 202 catastrophic ice storm events (defined as events causing more than $1 million USD in damages) occurred in the CONUS (Changnon 2007). The vast majority of ice storms occur in the eastern U.S. (88%), with most damaging events occurring within a band from the New England to Texas, excluding Florida. These events usually occur when surface temperatures are between 20 and 30° F and 90% of these events are less than 3 hours long (Coder 2015).

Freezing rain can occur via two distinct atmospheric processes: the melting process and the warm rain process. The melting process is more straightforward and occurs when an above-freezing, warm layer in the lower atmosphere is above a shallow below-freezing layer at the surface. The warm layer fully melts the precipitation, which supercools once it enters the shallow freezing layer at the surface. The warm rain process is more complex, and is also referred to “collision and coalescence.” All atmospheric layers are below freezing and supercooled water exists in the cloud, which do not freeze but remain supercooled when they fall to the surface. This process requires an atmosphere with significantly low dirt, dust, or other aerosols, and for these microscopic supercooled cloud droplets to collide, forming supercooled rain heavy enough to fall to the surface. The warm rain process usually results in less severe ice storms than the melting process, with regards to electricity infrastructure damage (Kovacik 2014). There is much uncertainty as to which of these processes is more common. Estimates of the share of melting process events range from 70% (Huffman and Norman 1988) to 25% (Reuber et al. 1999).

Few studies have attempted to estimate changes in ice storms caused by climate change. Cheng et al. (2006; 2010) use synoptic weather typing (principal components analysis, a clustering procedure, discriminant function analysis)—a statistical downscaling approach—to “estimate possible” changes in the occurrence of freezing rain events from climate change in Canada. In order to estimate these changes, a large number of variables are required—namely, hourly temperature, dew point, northward and eastward wind velocities at the surface and six upper atmospheric levels, mean sea-level air pressure, and total cloud cover. These studies find that for these locations in Eastern Canada, ice storms are likely to increase in the future, especially during colder months. Klima and Morgan (2015) use a “thought experiment” approach by shifting temperature profiles evenly to higher temperatures using atmospheric soundings from 38 weather stations in the U.S. and Canada east of the Rockies. The study finds that, as the temperature profile increases, there is a poleward shift in ice storm events as well as a shift towards winter. While many southern areas show fewer ice storms, ice storms in the North, as far south as Tennessee, show more frequent events.

While Regional Climate Models (RCMs) have been shown to simulate past ice storm events over Canada reasonably well (Bressen et al. 2017), there is less assurance that GCMs can replicate vertical temperature profiles, much less changes in vertical temperature profiles (Klima and Morgan 2015). The IPCC (2013) reports low confidence in the rate of change in the vertical structure of the atmosphere. In addition, the temperature profile can only account for the “melting process” ice storm events, which likely account for about half of all events, albeit the ones most likely the cause high damages.

For these reasons, we do not include the impacts of ice storms on electrical transmission and distribution infrastructure under climate change in this study.

## S.4 Climate Stressors – Details on Scenarios

Climate stressor information is input into our model in the form of one historical climate dataset, five future projections, and two greenhouse gas (GHG) emissions scenarios. These scenarios are consistent with those used in the second modeling phase of the Climate Change Impacts and Risk Analysis (CIRA2.0) project. The future climate projections are a subset of those generated for the Intergovernmental Panel on Climate Change’s Fifth Assessment Report (AR5). For climate forcing, two Representative Concentration Pathways (RCPs) are used: RCP8.5 and RCP4.5. RCP8.5 represents a future with substantial warming caused by higher GHG emissions, resulting in a total change in radiative forcing of 8.5 W/m^2^ by 2100 (compared to 1750). RCP4.5 represents a future with significant global reductions in GHG emissions, achieving a total radiative forcing of 4.5 W/m^2^ by 2100. Of the many GCMs generated for the AR5 as part of the Coupled Model Intercomparison Project Phase 5 (CMIP-5; Taylor et al. 2012), this study uses the following five: CanESM2, CCSM4, GISS-E2-R, HadGEM2-ES, and MIROC5.

These projections were downscaled using a statistically-based process that employs a multi-scale spatial matching scheme to select analog days from observations across CONUS (Pierce et al. 2014). This dataset, LOCA (Localized Constructed Analogs; U.S. Bureau of Reclamation et al, 2016) has a spatial resolution of 1/16 degree for daily maximum temperature, daily minimum temperature, and daily precipitation. Additional climate variables such as solar radiation, wind speed, and humidity, which required to estimate the stressors in this study, are developed using a binning approach, sourcing the historical values from the Princeton Land Surface Hydrology Group (Sheffield et al., 2006). For mapping purposes, each climate projection through 2099 can be split into four 20-year “eras”—2030 (2020-2039), 2050 (2040-2059), 2070 (2060-2079), and 2090 (2080-2099). Impacts in these future eras are compared to a “Control” scenario, which uses baseline climate over the years 1986-2005, with the added effect of population growth on changes in electricity demand.

**S.4.1 Climate Scenario Selection**

As in most climate impacts work, the selection of a subset of GCMs is necessary due to computational, time, and resource constraints. As such, five GCMs were chosen (summarized in

Table 1) with the intent of ensuring that the subset captures a large range of the variability in climate outcomes observed across the entire CMIP-5 ensemble.

Table 1: Summary of the five GCMs used in the analysis

| Center (Modeling Group) | Model Acronym | References |
| --- | --- | --- |
|  |  |  |
| National Center for Atmospheric Research | CCSM4 | Gent et al. 2011;  Neale et al. 2013 |
| NASA Goddard Institute for Space Studies | GISS-E2-R | Schmidt et al. 2006 |
| Canadian Centre for Climate Modeling and Analysis | CanESM2 | Von Salzen et al.  2013 |
| Met Office Hadley Centre | HadGEM2-ES | Collins et al., 2011;  Davies et al. 2005 |
| Atmosphere and Ocean Research Institute, National Institute for Environmental Studies, and Japan Agency for Marine-Earth Science and Technology | MIROC5 | Watanabe et al.  2010 |

**S.4.2 Variability in Climate Outcomes**

While many different metrics could be used in this type of comparison, a logical approach is to compare the projections from CMIP5 GCMs for annual and seasonal temperature and precipitation. While these averaged metrics may not be perfect substitutes for comparing extreme weather effects, the relationship should be sufficiently strong for selecting climate models from the broader ensemble.

The following scatter plots^^[[1]](#footnote-1)^^, Figures 1 and 2, show the variability across the CMIP5 ensemble for projected changes (2071-2100 compared to 1976-2005 baseline) in annual and summertime temperature and precipitation.^^[[2]](#footnote-2)^^


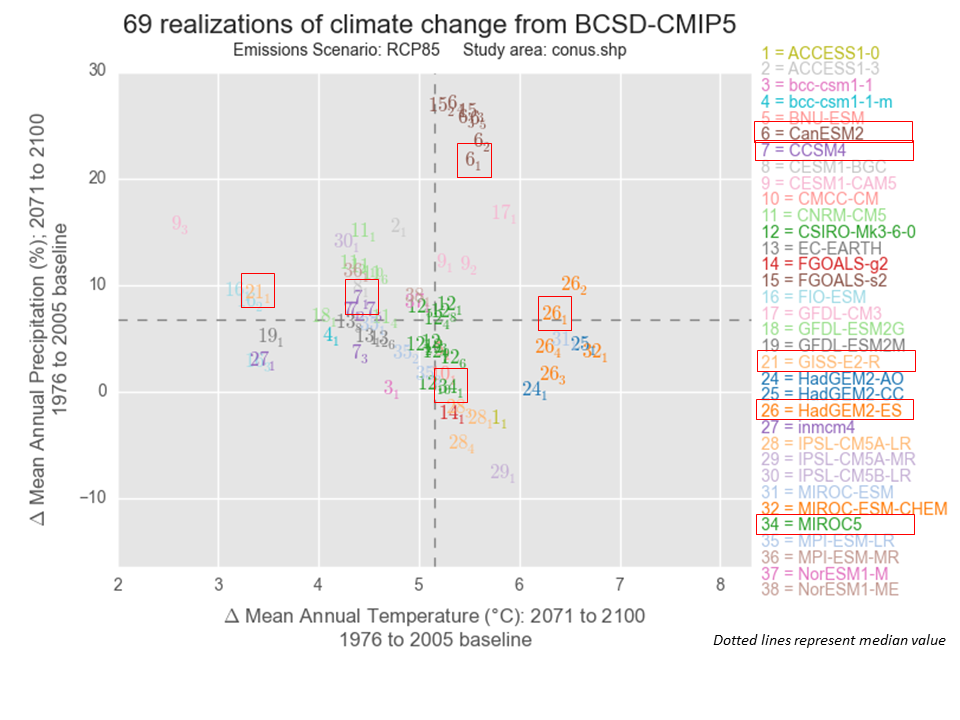


**Figure 1**: Variability of projected annual temperature and precipitation change across the CMIP5 ensemble for the CONUS


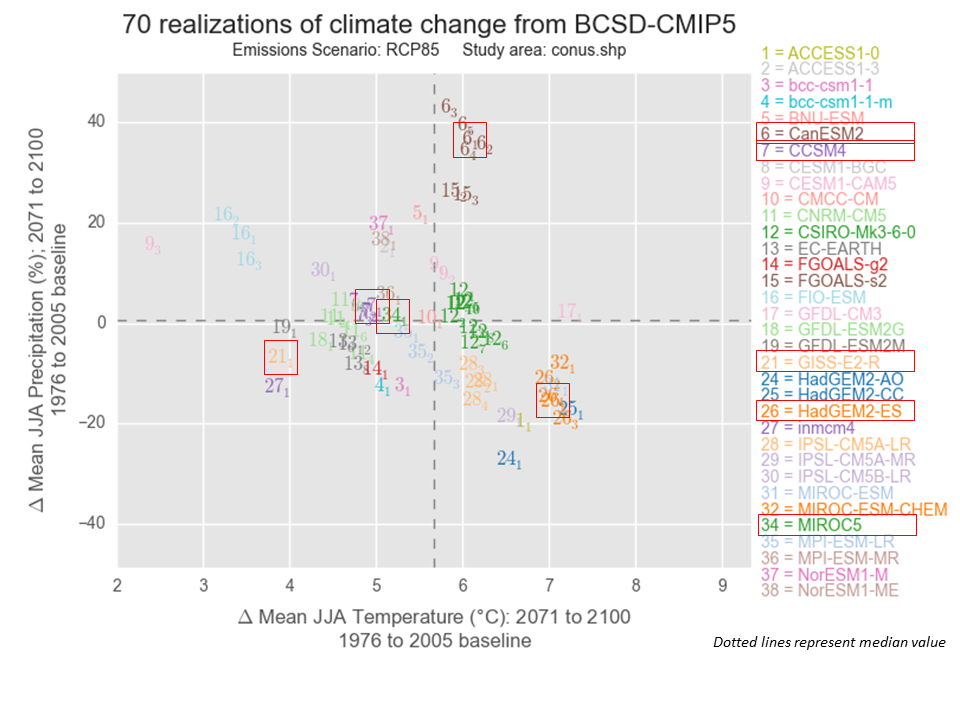


Figure 2: Variability of projected summertime temperature and precipitation change across the CMIP5 ensemble for the CONUS

As shown in Figures 1 and 2, the five selected GCMs (CanESM2, CCSM4, GISS-E2-R, HadGEM2-ES, and MIROC5) cover a large range of the variability across the entire ensemble in terms of annual and summertime temperature and precipitation. This selection also balances the range alongside considerations of model independence, broader usage by the scientific community, and skill at reproducing observed climate. Sanderson et al. (2015a, 2015b) provide analysis of both model skill at the global scale and independence of underlying code. These criteria were considered in the selection process.

## S.5 Infrastructure Inventory

While utilities keep record of their inventory, detailed information on electricity transmission and distribution infrastructure in the U.S. is not publically available. As a result, we construct a CONUS-wide infrastructure inventory for this study based on available spatial datasets as well as information on population, electricity demand and other variables / assumptions, combined with detailed information on specific utilities (that are used to calibrate relationships and assumptions applied elsewhere).

Table 2 provides a list of the attributes of the five types of infrastructure in the infrastructure inventory and the source of data or estimation procedure.

To compile the baseline infrastructure inventory for 2015, we utilized a variety of data sources from government databases, companies in the electric infrastructure market, and other reports on the electricity grid. Electricity demand data and population were also used to compile the infrastructure inventory.

Table 2 summarizes the five types of electricity infrastructure in our inventory, the important characteristics of each type of infrastructure, and the source of information for each characteristic. Information on transmission line miles, transmission line voltage, substations, and substation transformer voltage was obtained from the Homeland Infrastructure Foundation-Level Data (HIFLD) portal (HIFLD, 2017). Federal Energy Regulatory Commission (FERC) Form No.1, an annual report of major electric utility data, provided us data on the average number of large transformers per substation (FERC, 2018). To obtain data on distribution line miles and distribution transformers, we purchased the 2017 UDI Directory of Electric Power Producers and Distributors from S&P Global Platts (S&P Global 2018). Please contact S&P Global directly for more information regarding this database. The number of line miles and transformers at the utility level from the S&P data was weighted to the county level using and Electric Retail Service Territories shapefile from the HIFLD portal. For distribution power poles, we calculated the distance between power poles in rural versus urban areas, the height distribution of poles, and breakdown of pole material types using a spatial dataset of power poles owned by Green Mountain Power in the State of Vermont (Vermont, 2016). Data on distance between power poles was cross-referenced with data from Texas to ensure applicability outside the state of Vermont (Quanta-Technology, 2009). We calculated the voltage class of distribution lines based on height distribution of the power poles (SA.GOV.AU, 2017). For the diameter of distribution poles, we related pole class and height to the minimum diameter of the pole (McFarland Cascade, 2018).

Table 2: Infrastructure Inventory

| **Type of Infrastructure** | **Characteristic** | **Source** |
| --- | --- | --- |
| Transmission lines | Line Miles | HIFLD |
|  | Voltage Class | HIFLD |
| Distribution lines | Line Miles | S&P Global |
|  | Voltage Class | Relate to power pole height |
| Distribution poles | # Poles/Length of Cable | Extrapolated from Vermont Data |
|  | Height | Extrapolated from Vermont Data |
|  | Material | Extrapolated from Vermont Data |
|  | Diameter | Relate to power pole height |
| Substations | Count | HIFLD |
|  | Transformers | FERC Form 1 |
|  | Voltage | HIFLD |
| Distribution Transformers | Count | S&P Global |
|  |  |  |
| **Key:** |  |  |
| Direct data source |  |  |
| Approximate |  |  |

**S.5.1 Replacement Costs**

In order to determine average or typical replacement costs to use in our screening-level model, for the five types of infrastructure in our inventory, we draw upon a variety of sources. For the distribution system, we took the average cost per mile of constructing new overhead distribution lines between material and labor costs. We then divided these costs between the three types of infrastructure, assuming that labor costs are fully divided evenly between distribution pole and distribution line construction. This analysis was conducted separately for urban and rural areas as the costs are significantly different. The average cost per mile of constructing new overhead distribution line is $477,924 per mile in urban areas and $310,344 per mile in rural areas, in $2018 USD (Hall 2013). For overhead distribution lines, the material cost is 43.4% of the total while the labor cost is 56.6% of the total (Hall 2013).

Table 3: Replacement Costs for Each Infrastructure Type

| **Infrastructure Element** | **Unit Cost (Material and Labor)** | **Units** |
| --- | --- | --- |
| Wood Power Pole | $3,348 | per pole |
| Distribution Transformers | $2,651 | per transformer |
| Distribution Lines | $287,607 | per line mile |
| Transmission Lines 69 kV | $452,200 | per line mile |
| Transmission Lines 138 kV | $632,400 | per line mile |
| Transmission Lines 230 kV | $1,248,050 | per line mile |
| Transmission Lines 345 kV | $1,747,050 | per line mile |
| Transmission Lines 500 kV | $2,175,867 | per line mile |
| Substation Transformers | $5,312,766 | per transformer |

From here, we disaggregated the cost based on several other sources detailing the material costs of distribution infrastructure. First, however, we assume that the labor cost per mile of distribution line is evenly divided between the poles, transformers, and the line itself. Using the average distance between power poles in urban and rural areas, the cost per pole in urban and rural areas, and the total material cost for poles per mile, we were able to calculate the weighted average cost per pole at the national level. We obtained the weighted average material cost for each power pole using the Vermont state breakdown of percentage of poles for each pole class and height combination in urban versus rural areas (Vermont 2016) and industry data on the average cost per power pole for a given class-height combination (American Timber and Steel, 2018). For distribution transformers, we utilized Department of Energy data on the number of medium voltage distribution transformers of different type and the total value of these transformers to generate weighted average cost per transformer based on which transformers are most common (See Table 2, DOE 2014). For both urban and rural areas, this cost is $2,651 per transformer in 2018 USD. For distribution lines, we calculated the remaining portion of the overall material cost per line mile to construct new overhead distribution systems not already attributed to distribution power poles or distribution transformers. This total came to $287,607 USD per distribution line mile.

For transmission lines, we followed the guidance of a technical report from the WECC NERC region that provides methodology to calculate the cost per mile of transmission line (Pletka et al. 2014). Section 2.6 of this techincal report provides the following methodology for calculating total line cost (See Figure 3 below). Given that we are calculating replacement costs, we assume that the right of way (ROW) acres per mile is equal to zero as the ROW cost will be paid when the transmission lines are first constructed. For the 2014 base transmission cost, we utilize the numbers provided for 230, 345 and 500 kV double and single circuit lines for the base cost. For the 69 and 138 kV lines, which are also included in our infrastructure inventory, we draw baseline costs from a different technical document from the National Council on Electric Policy (See Table 3, Brown and Sedano 2004). For the conductor multiplier, we assume ACSR conductors because they are common for transmission infrastructure. For structure, we assume the tubular steel structure and for line length, we assume the default of greater than 10 miles of transmission line (Pletka et al. 2014). For the terrain multiplier, we assume a value of 1.0 for scrub/flat terrain and farmland. For each voltage category, we average across single and double circuit lines to obtain the cost per line mile. These costs are summarized in Table 3 of this Supplementary Material.

For large substation transformers, we draw on a DOE report that includes information regarding the cost of large transformers (See Table 2, DOE 2012). We follow this table and assume that the total cost of the transformer is on average 27.5% higher including labor and installation cost. Since we do not have detailed information on the breakdown of transformer types across the U.S., we take an average cost per transformer across all types of transformers provided in Table 2 of DOE (2012). The average cost per substation transformer is $5,312,766 in 2018 USD.

Figure 3: Total Transmission Line Cost Calculation (Pletka et al. 2014)


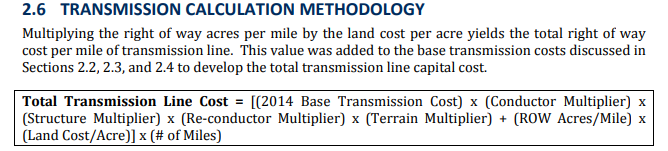


## S.6 Stressor-Response Relationships – Impact of Air Temperature on Power Lines

Rising ambient air temperature increases the resistance of a conductor and thereby decreases the carrying capacity of the cable. In combination with increasing demands from additional air conditioning usage, these decreases in capacity may create a bottleneck in the grid if extremely hot days become more common in the future. However, operators typically curtail the current in an at-risk conductor such that thermal limits are satisfied. Sathaye et al. (2011) evaluates this risk for California. Another study, Bartos et al. (2016), estimates these impacts for about 80% of the U.S. transmission lines, where an energy-balance approach is used. This approach calculates these capacity reductions by building thermal models of representative conductors and finds that summertime transmission capacity may be reduced by 2-6% by 2050. Rising temperatures can also increase line losses in the transmission system; however, Sathaye et al. (2011) find that these increased resistive losses caused by increased temperature are not expected to be significant in the next century.

We estimate decreases in line capacity using the method used in Bartos et al. (2016), which is based on the following equation.

$$I=\sqrt{\frac{\pi*h*D\left( T_{cond}-T_{amb} \right)+\pi*\varepsilon*\sigma*D*\left( {T_{cond}}^{4}-{T_{amb}}^{4} \right)-\delta*D*a_{s}}{R(T_{cond})}}$$

where I is the current, *h* is the average heat transfer coefficient, D is the cable diameter, T_cond_ is the conductor temperature, T_amb_ is the ambient air temperature, $\varepsilon$ is the emissivity of the conductor surface, $\sigma$ is the Stefan–Boltzmann constant, $\delta$ is the incident solar radiation, $a_{s}$ is the absorptivity of conductor surface, $R(T_{cond})$ is the resistance of the conductor at a given conductor temperature. Many assumptions need to be made to calculate the change in conductor capacity, all of which follow Bartos et al. (2016).

## S.7 Stressor-Response Relationships – Impact of Lightning on Power Lines

To understand the impact of lightning on electricity infrastructure, there are two key considerations: insulation level and the mechanism of lightning impact. These are described below.

## S.7.1 Insulation level of electric infrastructure

The minimum level of insulation is known as the Basic Insulation Level (BIL), which must be the minimum level of insulation of all the system’s components. Insulation levels are recommended for different voltage classes. The BIL of distribution infrastructure is deliberately kept less than that of other types of infrastructure (transmission, sub-station, electrical equipment) by a sufficient margin, so that in the cause of a failure, the utility (which is more equipped and capable of handling such infrastructure as compared to the customer) is able to fix it (Csanyi 2012). Additionally, distribution infrastructure contains the least costly components, and therefore failure of distribution infrastructure is less costly to fix as compared to transmission infrastructure. For more details, see Background on Insulation Coordination (Meliopoulus, 2007). We use the BIL by voltage provided by Csanyi (2012).

## S.7.2 Mechanism of lightning impact

The density of infrastructure in the vicinity (depending on their height and the height of other infrastructure in their vicinity, among other characteristics) governs the likelihood of different types of infrastructure being impacted by lightning (Meliopoulos 2007).

If lightning directly strikes the infrastructure, the probability of overvoltage occurrence in the insulation is high (practically = 1). This is not usually the case for distribution infrastructure, which is surrounded by other taller objects such as trees or buildings that are more likely to be directly hit by lightning. Transmission infrastructure, however, is vulnerable to direct lightning strikes. All direct events produce a flashover (Lopes et al. 2013).

In the case of transmission lines, it is possible that the overvoltages resulting from a lightning stroke to the shield wire will be of such magnitude as to cause a flashover from the shield wire to the phase conductor. This situation is called a backflashover. The risk of failure is calculated based on a combination of (i) likelihood of direct impact (ii) likelihood of failure caused by backflashover

In the case of distribution network, it is possible that the overvoltages resulting from a indirect lightning impact (i) distribution transformers (ii) distribution lines. The induced lightning overvoltages are of concern for distribution lines 35 kV or below. Higher kilovolt-level lines (i.e. 69 kV and above) have sufficient insulation so that induced lightning voltages do not present the risk of flashover (Meliopoulus, 2007).

## S.7.3 Caveats for all Assumptions

Very broadly, lightning impacts to electrical infrastructure are dependent on:

1. Location of strike
2. Nature (i.e. one or series of strokes) and intensity of stroke (intenstity can range from 1 kA to 200 kA, with varying time-to-peak of overvoltage, based on voltage-time waveform)
3. Type of infrastructure, including type of lightning shielding used (arresters, grounding, pole impedance)
4. Soil resistivity
5. See others caveats in Meliopoulus (2007)

All sources used to develop this methodology are based on simulation of lightning strike on a particular infrastructure. The extrapolation of results from these calculations is therefore highly dependent on the use of reasonable assumptions.

## S.7.4 Direct strikes on transmission lines

Using the data on lightning strikes in each grid, it is possible to calculate the probability of the lightning to hit the transmission line by calculating the vulnerable area based on striking distance, and therefore the probability of the lightning strike. This is simply calculated by taking the length of transmission lines in a county, and dividing by area of the county. The number of direct strikes (N) is calculated using the formula provided in Erickson (1987)

$$N=N_{g}\left( b+28H^{0.6} \right)*{10}^{-1}$$

Where N_g_ is the number of strikes to the ground, and H is the height.

Once the number of direct strikes are calculated, the impact on transmission lines caused by direct lightning strikes is calculated using a combination of shielding failure rate (failure of shielding line, which protects the phase conductor from lightning strikes) and backflashover rate (flashover of phase conductor from overvoltage in the shield wire). The measure used to represent transmission line failure is shielding failure flashover rate or SSFOR, (Mikropoulus, 2010):

$$SFFOR=0.2N_{g}\int_{I_{C}}^{I_{MSF}} W\left( I \right)f\left( I \right)dI$$

$$I_{C}=2(CFO)/Z_{s}$$

I_MSF_ and W(I) depends on tower parameters (height of shielding wire, height of phase conductor, horizontal distance between shielding wire and phase conductor). f(I) depends on values of lightning current (median, standard deviation). Zs is Impedance, where we use 800 ohms, for a typical transmission design line (Meliopoulus, 2007) and for CFO, we use 1200 kV (NERC 2012).

## S.7.5 Indirect Strikes on Distribution lines

The distribution system is most impacted by indirect strikes. All direct events cause a flashover, but since the probability of occurrence of a direct event is minimal (in rare cases in rural areas where there are few trees), it is ignored in this calculation. There are multiple caveats while assessing the impacts of lightning strikes on distribution lines, and assumptions have to be made.


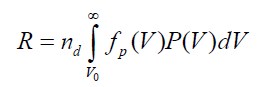


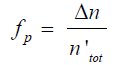


Where delta n is the number of indirect events generating induced voltages of amplitudes from V to V+delV, and n’tot is the number of dangerous events (i.e. overvoltages larger than the chosen minimum value).

We assume 1: 46 kV line (middle between the distribution size lines), which has a BIL = 200 kV (based on Csayni, 2012). Based on Piantini (2008), the risk of lightning for a BIL of 200 (25-33 KV line range), is 0.03 (i.e. the risk of failure). This reflects that annual number of events that will cause overvoltages larger than the insulation. We have to adjust this by the value of N_g_, i.e. the total number of lightning strikes in each county. Next, we will adjust this value with the density of distribution lines in each county. For a density equivalent to 2 km/6*4 i.e. 0.08 km/km2, we will assume Ng * 0.03 = risk of failure. We linearly increase this with the density of distribution lines. A standard striking distance for this analysis is 20m. Using the density of the distribution system in each county, it is found that the likelihood of a direct lightning strike in a grid that contains 100 km of distribution line is (100*1000*20 / 50000 * 50000) = 0.0008 (calculated using 20m distance around the line, in a 50 x 50 km grid), although this is an overestimate since many distribution lines are close to each other, on either side of the road, for example).

Distribution transformers and lines are usually closely connected, and a lightning impact on one often causes a similar impact in the other. Similar to transformers, distribution lines may be vulnerable to overvoltages resulting from lightning strokes to nearby trees, ground, or other objects. These voltage surges are known as induced lightning voltages and are injected into the power system through coupling. These surges are of concern for distribution lines of 33 kV or below, which have a low critical flashover voltage (CFO). (Izadi, 2017; Meliopoulus 2007).

The steps to calculate the probability of impact of lighting on distribution lines is as follows:

1. We consider distribution lines of 33 kV or below by using a proportion of the total distribution line miles in each county/grid. Therefore, the density of distribution lines and the number of strikes in the area determine the likelihood of failure strikes.
2. Using a distance of 20 m from the distribution line, we calculate the probability of lightning strike (indirect, through other objects) to the distribution line. {Need Reference for 20m}
3. Using Csayni (2012), calculate the % of the lightning strikes after Step(ii) result in failure

## S.8. Stressor-Response Relationships – Impact of Wildfires on Power Lines

Wildfires can also have impacts on power lines. Larger transmission lines are not usually destroyed by wildfire, but smaller distribution lines can be because these are held in place by timber poles. However, these lines are often near buildings or residential areas and are likely to be protected by fire fighting. For this reason, we do not model wildfire destruction. Other impacts caused by wildfires may be more costly than direct destruction (Aspen Environmental Group 2008; CAISO 2009). The transmission capacity of a line can be affected by heat, smoke, and particulate matter from a fire even though the lines are not in the burnt zone. Insulators that attach the lines to the towers can accumulate soot, creating a conductive path and causing leakage currents that may force the line to be shut down. Ionized air in smoke can act as a conductor, causing arcing; either between lines, or between lines and the ground, that results in a power interruption. Finally, even if the lines are protected from fire, the effects of firefighting can also negatively affect transmission operation either by aircraft dumping loads of fire retardant that can foul the lines, or through preventive shutdowns for safety measures (Sathaye et al. 2011).

Transmission infrastructure is impacted due to heat caused by wildfires, leakage currents due to ionized air in smoke, and deposition of soot on insulators. Redundancy in the transmission network will minimize outage-related impacts of wildfires and the efficiency losses due to ionized air in smoke will not be significant, due to the occasional occurrence of wildfires. This paper therefore only considers the destruction of transmission lines attributable to wildfires, and uses the average repair cost of transmission lines to quantify the impact of wildfires on transmission infrastructure.

While clearance requirements for transmission lines mitigate their exposure to wildfires, maintenance in forested areas is challenging. Heat from nearby wildfires, despite clearance requirements, can impact transmission lines and poles that are exposed to wildfires. Wildfires that burn a significant amount of biomass can elongate the transmission lines, creating sag, and in extreme cases, failure due to excessive load on transmission towers. Although transmission lines that are lower than 30 meters are considered to be vulnerable to wildfire flames, this analysis only considers transmission lines that are less than 69 kV in capacity, which have been found to be impacted by wildfires in San Diego County (Johnson, 2014). The following steps provide details of the probabilistic method used to estimate the impacts of wildfires on transmission lines:

Step 1 – Calculate Vulnerable Area in Each Gridcell. Using the projected area burned in each gridcell, calculate the probability of wildfire in the gridcell by dividing the area of wildfire by the total area of gridcell.

Step 2 – Calculate Relative Weight of Vulnerable Transmission Lines Presence in Each Gridcell. A weight of vulnerable transmission line (69 kV or less) presence is developed for each gridcell that constitutes a county using the data on length and capacity of transmission lines in each gridcell. The relative weight of transmission line presence (wi) in each gridcell in the county ranges from 0 to 1, with 0 representing no vulnerable transmission lines and 1 representing all transmission lines in the county being contained in the gridcell.

Step 3 – Calculate Probability of Wildfire in the Transmission Network in each County. The probability of wildfire at any point along a transmission line is calculated as: P = 1 - ∑(1 – piwi), where pi is the probability of fire in the gridcell, and wi is the relative length of the line in the cell. Adding the various combinations provides the total probability of wildfire impacting a vulnerable transmission line in the county. Since the probability of wildfire and their relative weight is calculated individually, the formula reduces to ∑ piwi.

Step 4. Estimate length of damage and repair costs of transmission lines due to wildfires. The probability of exposure of the vulnerable transmission line network in the county to wildfires is used to estimate the total replacement cost. The propagation characteristics of wildfires are highly dependent on the landscape and nature of forests in the region, and therefore the length of vulnerable transmission lines affected by wildfires is difficult to estimate at a national scale, especially since the wildfire data is available only as an area in each gridcell. The methodology assumes that if a wildfire occurs, at least 1 km section of transmission line will be destroyed and therefore will need to be replaced. The total replacement cost of transmission lines due to wildfires is estimated using a per-mile replacement cost of $1.92 million (or $1.19 million per km) for 69 kV (or lower) transmission lines.

## S.9 Stressor-Response Relationships – Timber Pole Degradation

With proper maintenance, the lifespan of a timber pole is generally considered to be about 30 to 40 years (Beyond Pesticides, 2005; AISI, 2005; Western Wood Preservers Institute, 1996). However, location and climate can also impact timber pole degradation. Degradation rates are usually higher in warmer, humid regions of the CONUS (Morrel 2016). However, regions with higher degradation rates usually treat the pole to account for the varying degradation rates within these zones, following the American Wood Protection Association (AWPA) standards.

Wang and Wang (2012) evaluate the impact of changes in climate on timber pole degradation for three cities in Australia. The study finds that by 2080, the median decay rate would increase for two of the cities by about 10% and decrease for the third city by about 12%. The general form of the relationship between climate and hardwood decay rate (caused by fungal attack) is as follows (Wang et al. 2008).

$$r=k_{w}{f\left( R \right)}^{0.3}{g(T)}^{0.2}$$

where r is decay rate, kw is a wood-specific parameter, f(R) is a function that includes rainfall, R, and g(T) is a function that includes temperature, T.

Decay reduces the diameter of the pole, which then reduces the pole strength. The strength of the pole is estimated as (Wang et al. 2008) below, where D is the original diameter and d is the diameter reduction.

$$S=\frac{\pi}{32}*{(D-d)}^{3}$$

Assuming that timber poles are properly maintained and the original treatment followed AWPA standards to ensure the expected life of the pole, we approximate changes in the pole strength over time with and without climate change, and use these changes in strength to change the expected life of a pole. We assume a wood pole will need to be replaced once it reaches 60% of its original strength. This gives an average pole lifespan of about 40 years nationally. The estimated service life of poles in the U.S. is about 32 years (Morrel 2005) but many other factors like major climatic events (hurricanes and ice storms) or replacement from widening roads play a role in reducing pole lifespan.

## S.10 Stressor-Response Relationships – Impact of Temperature on Transformer Failure

As the transformers age, the probability of failure due to overheating increases. He et al. (2009) develops a relationship between transformer failure rate and transformer age as below.

$$P_{a}=1-exp\left[ \left( \frac{I_{e}}{C_{exp}\left( \frac{\beta}{\theta_{0}} \right)} \right)\beta-\left( \frac{I_{e}+\Delta t_{e}}{C_{exp}\left( \frac{\beta}{\theta_{0}} \right)} \right)\beta\right]$$

where P_a_ is the probability of failure; I_e_ is the sum of the lost insulation life; t_e_ is the equivalent operation time; C and β are the life parameter and shape parameter, respectively.

Failure of a substation transformer would likely result in loss of expensive equipment and possibly widespread outages. However, failure due to heat waves is somewhat rare and excluded and heat-wave extreme events are not well-characterized in GCMs (Gao et al. 2017). For these reasons, heatwave failure is excluded from this analysis.

## S.11 Stressor-Response Relationships – Impact of Direct Lightning Strike on Transformers

Transformers are occasionally damaged by lightning strikes (Wang et al. 2002). However, damage to transformers is heavily dependent on the distance from the strike zone to the transformer along the power line. There is little information on distribution transformer locations. In addition, many substations are well-protected from lightning damage. For these reasons, direct lightning strikes on transformers are not considered in this analysis.

## S.12 Stressor-Response Relationships – Impact of Temperature on Transformer Lifespan

Transformer lifespan is also dependent on ambient air temperature. Large Power Transformers (LPTs) are typically cooled with oil-based convective heat sinks that circulate oil around the windings, which expel heat via fans or other mechanisms. These cooling systems sometimes develop “hot spots,” which can damage the insulating paper that protects windings from short circuits. Short circuits can lead to failures, which cause blackouts and require repair or replacement. Replacing LPTs can be a complex process taking years (Gao et al. 2017). However, hot spot failures rarely occur because LPTs are one of the single most expensive pieces of infrastructure on the electric grid (US DOE, 2012) and are designed to withstand extreme heat.

In the long-term, though, warmer operating temperatures reduce the expected life of an LPT, which ages the insulating paper between the windings. This relationship can be expressed as (Lundgaard et al. 2004):

$$Expected Life=\left[ \frac{\frac{1}{{DP}_{end}}-\frac{1}{{DP}_{Start}}}{A*24*365} \right]*exp\left( \frac{13350}{T} \right)$$

where DP_Start_ and DP_End_ are the degree of polymerization of the cellulose at the beginning (≈ 1000) and end of the transformer life (≈ 200), respectively; A is the pre-exponential factor, determined to be 2E+8, and T is the temperature in Kelvin. Assuming a design life of 40 years (Metwally, 2011; US DOE, 2012), this corresponds to a temperature of about 82°C and is close to the IEEE standard (IEEE, 2012).

A simple thermal model can be used to relate ambient air temperature to operating temperature to consider long-term impacts temperature (Gao et al. 2017).

$$\theta_{top}=\theta_{0}+\theta_{amb}$$

$$\theta_{top}={(\theta}_{u}-\theta_{i})(1-exp(1/{T_{0}}))+\theta_{i}+\theta_{amb}$$

where θ_top_ is the top oil temperature in °C; θ_0_ is the initial top oil temperature in °C; θ_u_ is the ultimate top rise temperature for a load L in °C; θ_i_ is the initial top rise temperature in °C for time t = 0; and T0 is the time constant at rated power in hours.

Gao et al. (2017) mentions that, all else equal, this indicates that for every 1°C rise in ambient temperature, the expected lifespan would decrease by about 10%, or 4 years.

Smaller distribution transformers, typically either pole-mounted or pad-mounted, serve as the last voltage step-down devices in the distribution network before supplying electricity to customers. These are much less expensive individually but there are far more of them compared to larger transformers and therefore makeup a large capital and expense for utilities. Assessing the lifespan of these distribution transformers follows a similar structure and mechanism as large transformers but the equations differ. For these, we use the procedure developed by Stahlhut et al. (2008) at steady state, which is based on the ANSI/IEEE Standard C.57.91. The loss of life of distribution transformers depends on the load profile and temperature, one of the components of which is ambient temperature.

The loss of life (LOL) is estimated as

$$LOL=100t ({10}^{-\left[ A+ \frac{B}{T} \right]})$$

Where t is the time-step at constant load which is calculated at the hourly level; A (-11.269 hours) and B (6328.8 hours/Kelvin) are parameters specific to the 65 degree rise transformer, which we use as a representative distribution transformer.

The temperature, T, is estimated as

$$T=273+ \theta_{a}+ \theta_{o}+ \theta_{g}$$

Where θ_a_ is the ambient temperature, θ_o_ is the is the top-oil rise over ambient temperature, and θ_g_ is the hottest spot conductor rise over top-oil temperature.

## S.13 Stressor-Response Relationships – Impact of Sea Level Rise and Storm Surge on Transformers

Although sea-level-rise and storm surge are likely to have impacts on poles, lines, and distribution transformers, our analysis is limited to the impacts to substations because we can identify and estimate substation elevations and locations along the coast. These impacts are estimated using inundation projections from the National Coastal Property Model (NCPM; Neumann et al. 2015). The model was modified to include substations using the flood damage curves developed for the Federal Emergency Management Agency’s Hazus model to estimate storm surge damage. We assume that when a substation is inundated by sea level rise, utilities will relocate the substation. This relocation cost is estimated using the salvage value of a substation in mid-life cycle, which is about 25% of the original construction costs (Hoff and Shugar n. d.)). All new substations and those relocated are assumed be built in an area that will not be subject to damages from either storm surge or sea level rise.

## S.14 Economic Impacts – Estimating Power Interruption Costs

Back-of-the-envelope estimates of the costs of power interruptions were completed using the model described in Sullivan et al. (2009; 2015). This model, the Interruption Cost Estimate (ICE) calculator, is based on customer willingness to pay surveys conducted by utilities and follows the structure below.

$$ICE=f\left( MixCust, Income, Consumption,CAIDI, other \right)$$

This equation approximates individual interruption costs per customer as a function of the regional average mix of residential, small, and medium/large commercial and industrial customers (MixCust); median household income by region (Income); annual electricity consumption (Consumption); and the average duration of an individual interruption, or Customer Average Interruption Duration Index (CAIDI). Other inputs to the model include backup equipment and type (Gen and/or Power Conditioning), customer mix by industry—namely, construction or manufacturing, time of day (morning, afternoon, night), and time of year (summer / non-summer). Inputs in the ‘other’ category are derived from the online version of the ICE model (icecalculator.com) at the state-level.

We estimate outage costs using two different methods: relative and absolute. “Relative” costs are derived using historical data and applying the change in the occurrence of outages using our estimates of changes in stressor-response relationships. Many of the stressor-response relationships are not well represented by historical data, e.g., substantial changes in the long-term mean ambient temperature, impacting transformer lifespan, has not happened yet and therefore we assume this impact is not well-characterized with historical information. Other stressor-response relationships are not well-characterized by historical data because the historical data available does not identify outages caused by that particular stressor-response and therefore we are limited via a data gap. In these cases, we calculate an “absolute” cost, where we use the typical cost of a single outage resulting from the stressor-response relationship and simply multiply these by the change in that stressor-response. The following table displays which stressor-response relationships are estimated using a relative method, which are estimated using an absolute method, and the ones excluded from the outage estimation.

Table 4: Interruption Costing Methods

| **Stressor** | **Costing Method** |
| --- | --- |
| Vegetation Management | Relative |
| Lightning (Distribution) | Relative |
| Storm Surge (Substations) | Absolute |
| Wood Pole Decay (Lifespan) | Absolute |
| Distribution Transformer (Lifespan) | Absolute |
| Distribution Line (Capacity) | Absolute |
| Wildfires on Transmission Lines | Relative |
| Lightning (Transmission) | Relative |
| Substation (Capacity) | Excluded |
| Substation Transformer (Lifespan) | Excluded |
| Transmission Line (Capacity) | Excluded |
| Sea Level Rise (Substations) | Excluded |

We exclude substation capacity and lifespan because substations are fairly well monitored compared to, for example, distribution transformers. We assume it is unlikely that a substation would be the cause of an outage because of changes in capacity or lifespan. Similarly, transmission lines are typically designed with redundancy and ambient temperatures are taken into account by system operators (Bartos et al. 2016). Finally, since sea-level-rise is a slow process and obvious when it becomes a concern, we assume substations are relocated or salvaged before sea-level rise causes an outage.

The historical data used for deriving the relative costs of outages from Mukherjee et al. (2018) is derived from publically available datasets including data from U.S. Department of Energy (DOE), U.S. Energy Information Administration (EIA), National Oceanic and Atmospheric Administration (NOAA), among others. This database includes the cause, number of customers (by type) impacted, duration, and State. However, these data only represent major outages. Other databases that include a more comprehensive account of outages such as the EIA Reliability Statistics, do not report many of necessary characteristics, specifically, the cause of the outage. Based on the EIA Reliability statistics for the overlapping years (2014-2016) we estimate that the major outage database from Mukherjee et al. (2018) accounts for roughly 10% of the total customer-minutes of outages in CONUS. For this estimation we exclude outages that did not provide a cause in the major outage database. We use this database to estimate the outage costs of three stressor-response relationships: vegetation management, wildfires, and lightning. We attribute these three using the assumptions provided in the following table, where the outage costs are estimated using the ICE model. The assumed historical attribution table was derived via expert judgement and consultation.

Table 5: Historical Costs and assumed attributions for each climate stressor

| **Cause** | **Cost ($Bill/year)** | **Cost (%)** | **Events (%)** | **Assumed Historical Attribution** | | |
| --- | --- | --- | --- | --- | --- | --- |
|  |  |  |  | **Trees** | **Lightning** | **Wildfires** |
| Hurricanes | $1.83 | 22.80% | 9.70% | 80% |  |  |
| Unspecified | $3.55 | 18.80% | 24.50% |  |  |  |
| Winter Storm | $1.46 | 18.00% | 13.20% | 80% |  |  |
| Thunderstorm | $2.95 | 15.80% | 23.30% | 70% | 10% |  |
| Storm | $0.52 | 7.30% | 5.40% | 50% |  |  |
| Heavy Wind | $0.89 | 7.00% | 8.00% | 80% |  |  |
| Wildfire | $0.19 | 2.40% | 3.40% |  |  | 100% |
| Snow/Ice | $0.05 | 2.10% | 1.80% | 80% |  |  |
| Wind/Rain | $0.14 | 1.60% | 1.60% | 50% |  |  |
| Heatwave | $0.00 | 1.10% | 1.30% |  |  |  |
| Flooding | $0.02 | 0.20% | 0.50% |  |  |  |
| Lightning | $0.02 | 0.10% | 0.40% |  | 100% |  |
| **Total CONUS** | **$21.29** | 100% | 100% | **$5.77** | **$0.31** | **$0.19** |

Absolute outage cost approximations are used for storm surge (on substation transformers), wood pole decay, distribution transformer lifespan, and distribution line capacity. These are estimated simply, based on expert consultation. For storm surge, we assume that if the surge reaches a depth of 4 ft, which is used in the Hazus Flooding Model, the substation is no longer functional and therefore an interruption occurs. For wood pole decay, we assume that if wood pole lifespan reduces to at least 5 years earlier than expected, an outage occurs. Similarly, for distribution transformer lifespan, we assume that if the transformer lifespan reduces to less than 5 years, an outage occurs. For distribution line capacity, we assume an outage occurs if the ampacity reduces by 10% in a given year, as compared to the historical worst case (but only once for a given year and location).

## S.15 Adaptation Measures

The following table outlines the adaptation measures used to assess the range of utility responses to climate change impacts. These adaptation options are not meant to be the most cost effective option in all circumstances – i.e., no cost-benefit analysis was applied since adaptation was not the main goal of this study. Instead, these are meant to be reasonable adaptation options a utility may choose in response to certain stressors in order to address additional costs caused by changes in climate for the goal of providing reasonable cost ranges to the variability in utility response.

Table 6: Adaptation applied for each stressor-response function

| **Stressor-Response** | **Adaptation Used** |
| --- | --- |
| Transmission line capacity | Ampacity Upgrade (ACSR->ACSS) |
| Distribution line capacity | Ampacity upgrade (ACSR->ACSS) |
| Wood Pole Decay | Steel reinforcement |
| Substation transformer lifespan | Build additional transformers |
| Distribution transformer lifespan | Build additional transformers |
| Substation SLR | Armoring (proactive only) |
| Substation Storm Surge | Armoring (proactive only) |
| Vegetation Management | No options |
| Wildfire repair | No options |

For transmission and distribution line capacity, we assess the utility response by allowing utilities to install Aluminum Conductor Steel Supported (ACSS) cable instead of the conventional Aluminum Conductor Steel Reinforced (ACSR) cable. ACSS costs are higher but exhibit very low sag at high temperatures, allowing for more current in the cable during hot summer days. Costs and added ampacity are based on EPRI (2008), with a price multiplier of 1.2 and an ampacity multiplier of 1.9.

For wood pole decay, we allow utilities to apply structural rehabilitation via steel reinforcement to the base of the pole. Other options are, of course, available such as replacing the wood pole all-together with either steel or concrete poles, more frequent chemical treatment of poles, or more frequent inspection, among others. For the cost of the reinforcement, we use a flat rate of $500 per pole based on REF, which we assume extends the pole life to 50 years, which is on the high end of the baseline lifespans across CONUS, the vast majority range from about 30-50 years.

Adaptation for transformer lifespan (both types) is dealt with in our model by building additional transformers until the capacity of the fleet of transformers is raised so as to reduce the load on any individual transformer. For substation transformers, we apply the method described in Sathaye et al. (2013) to estimate the additional capacity required to bring the hot-spot temperatures of the transformer to baseline (historical) conditions. For distribution transformers, we calculate the additional capacity required by reducing the load incrementally until the loss of life of the transformer is reduced to baseline conditions using the methods outlined in Stahlhut et al. (2008), which accounts for the effect of load.

In response to sea-level rise and storm surge, utilities protect the substation with a sea wall in our model. This approach follows the National Coastal Property Model (NCPM) framework and costs, which was used in this part of the analysis. Briefly explained, whenever a substation is vulnerable to sea-level rise, the NCPM compares the value of the substation with the cost of a sea wall (which varies by location). If the value of the substation is greater than the cost of the sea wall, the substation is protected. If the cost of the sea wall is greater than the value of the substation, the substation is salvaged. In response to storm surge, the decision is made the same way, except that the damage to the substation (as a fraction of total value) is used instead of the total value to compare with the sea wall cost.

## S.16 Maps of Infrastructure


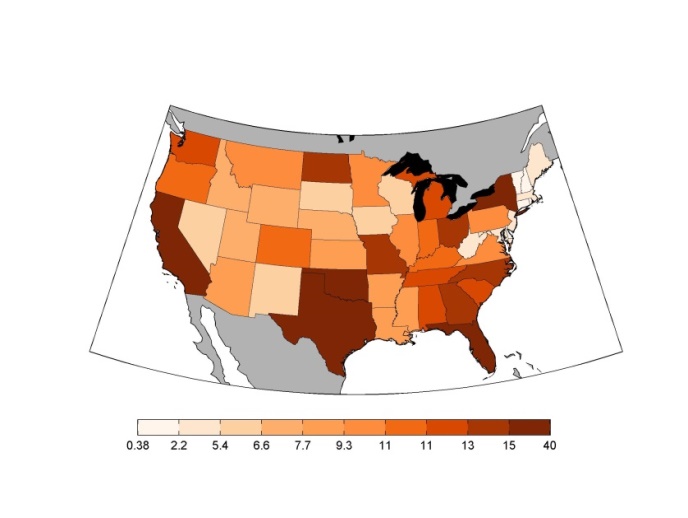

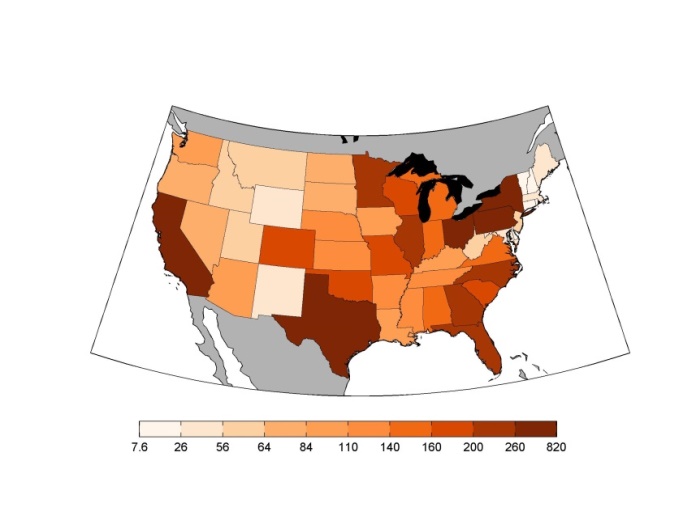

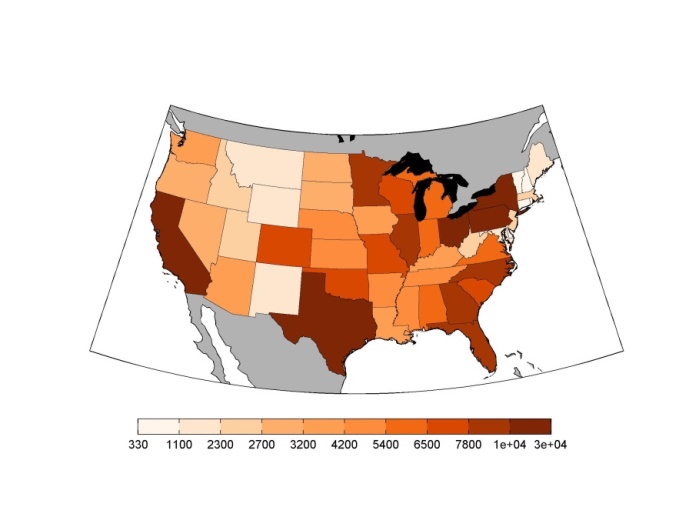

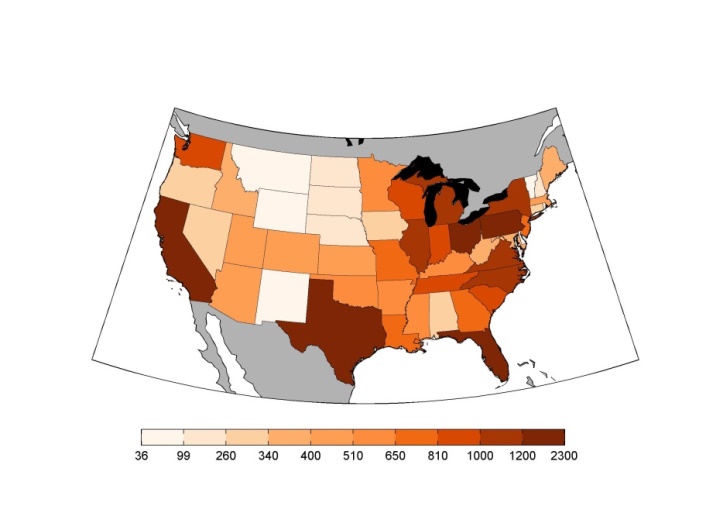


Figure 4: Infrastructure in CONUS for baseline infrastructure, year 2015, at the state level (based on county-scale modeling). On the upper left, transmission line miles (thousands); upper right, distribution line miles (thousands); lower left, distribution poles (thousands); lower right, distribution transformers (thousands).


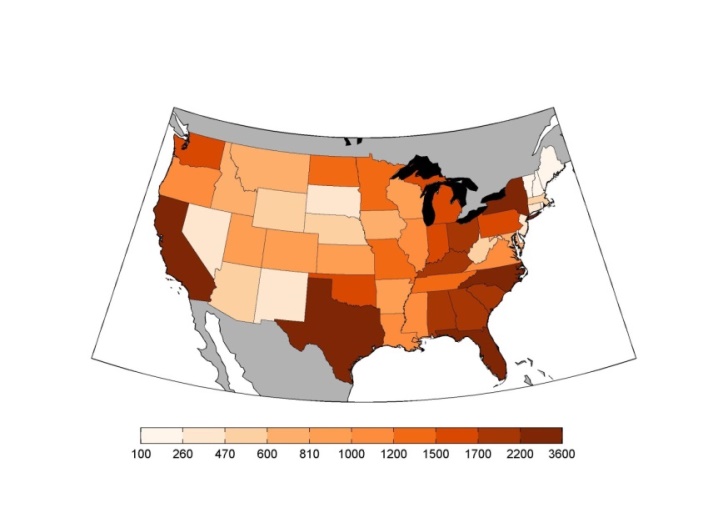


Figure 5: Total number of electric substations in CONUS for baseline infrastructure year 2015 at the state level (based on county-scale modeling).


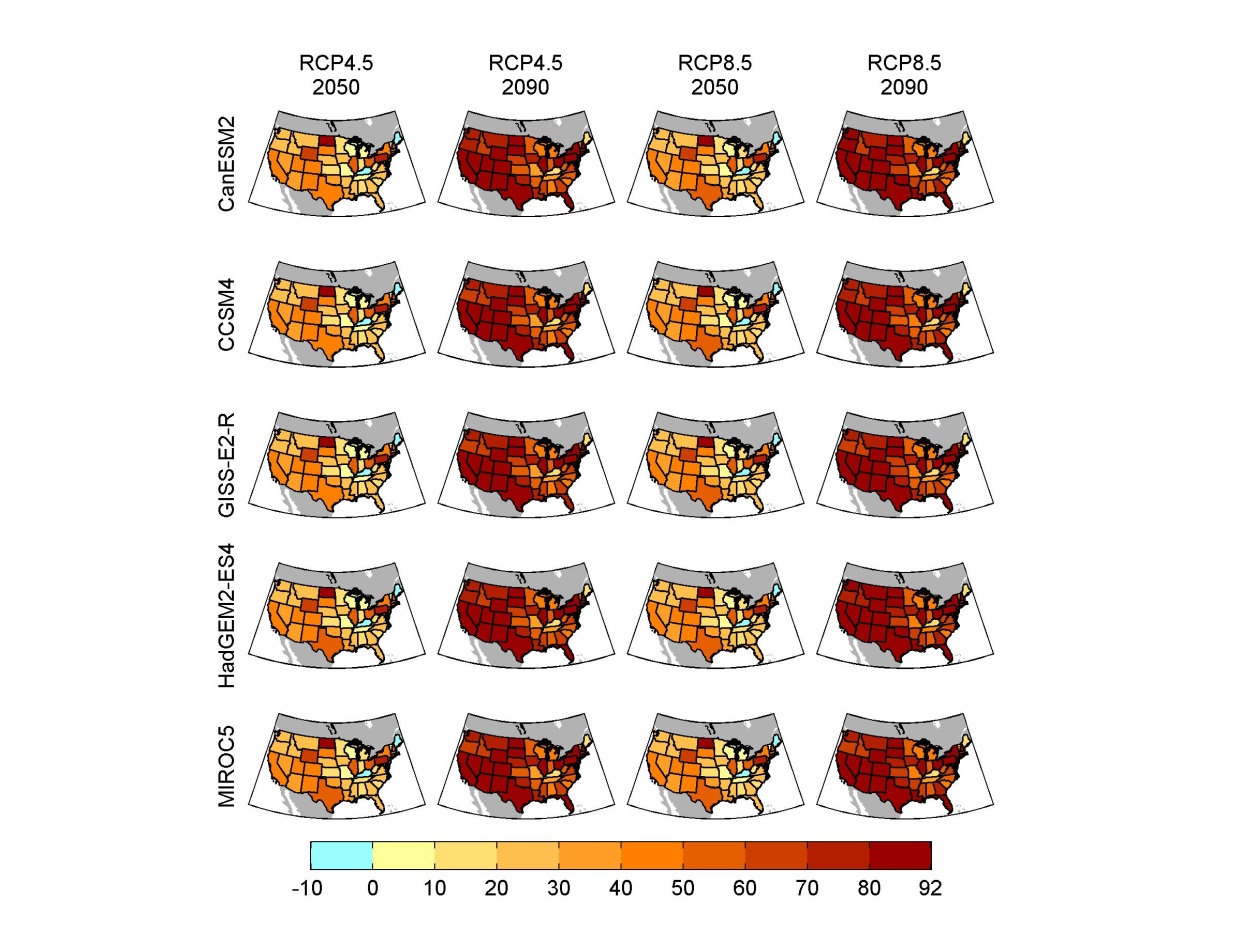


Figure 6: Infrastructure Projections (% change; based on projections in demand, which differ in response to temperature changes) from 2015 to the 2050 era (2040-2059 average) and the 2090 era (2080-2099 average) for each GCM and RCP combination at the state level.

## S.17 Maps of Climate-Driven Stressors


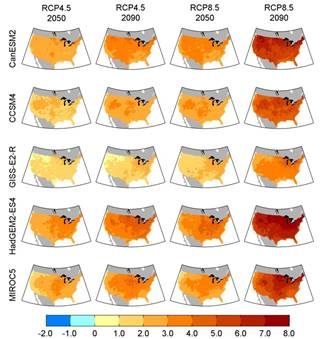


Figure 7: Percent change in annual temperature from baseline (1986-2005) to the 2050 era (2040-2059 average) and the 2090 era (2080-2099 average) for each GCM and RCP combination at the county level.


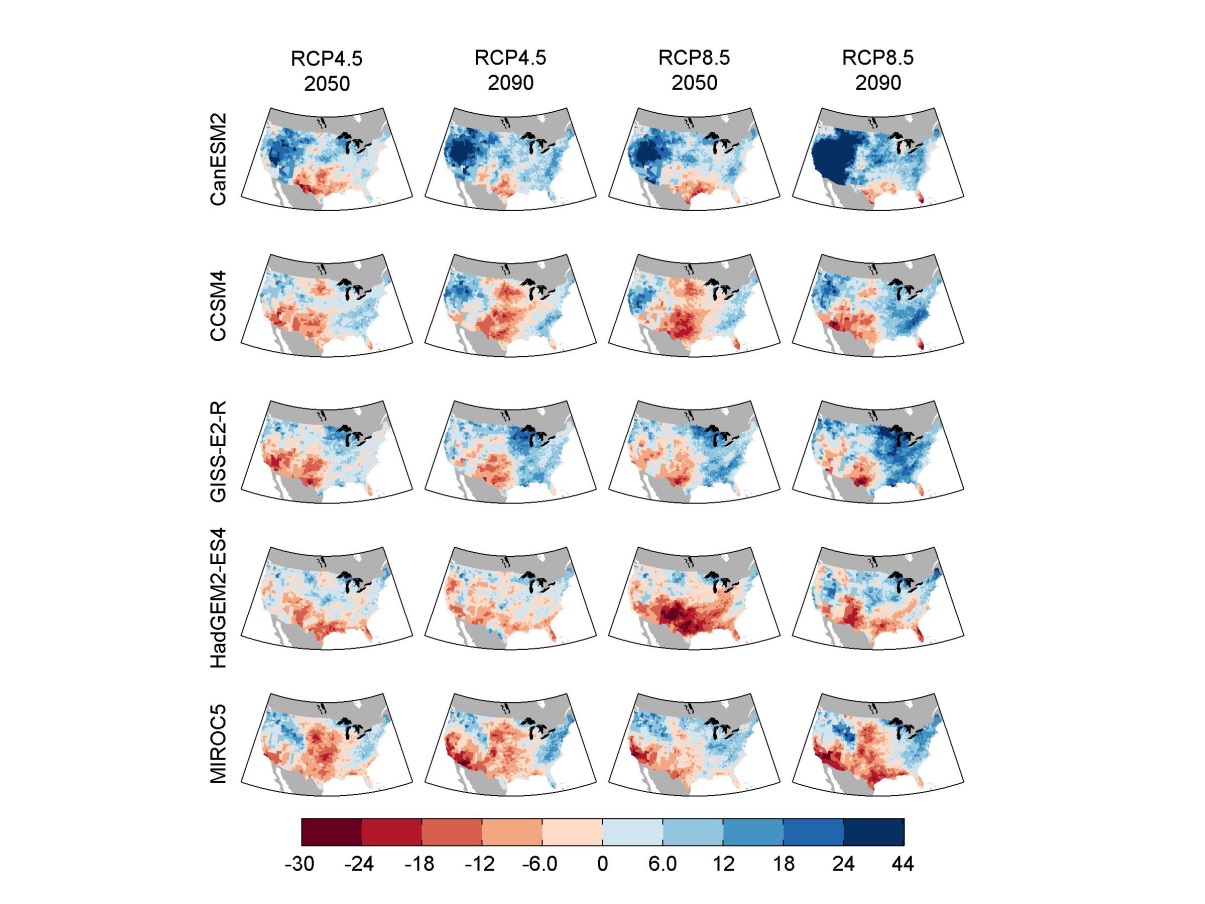


Figure 8: Percent change in annual precipitation from baseline (1986-2005) to the 2050 era (2040-2059 average) and the 2090 era (2080-2099 average) for each GCM and RCP combination at the county level.


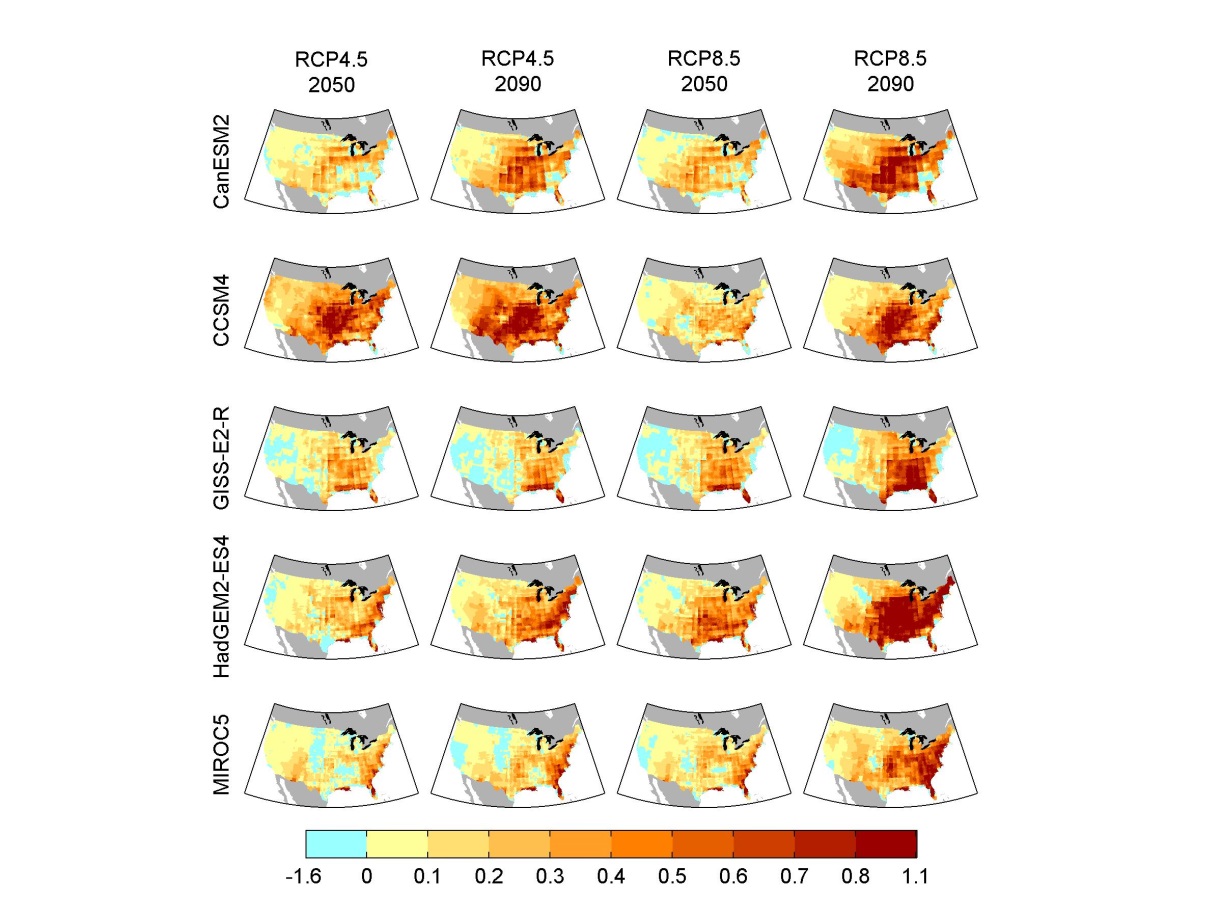


Figure 9: Absolute change in total number of lightning strikes per 100 meters^2^ from the baseline climate era (1986-2005) to the 2050 era (2040-2059 average) and the 2090 era (2080-2099 average) for each GCM and RCP combination at the county level.


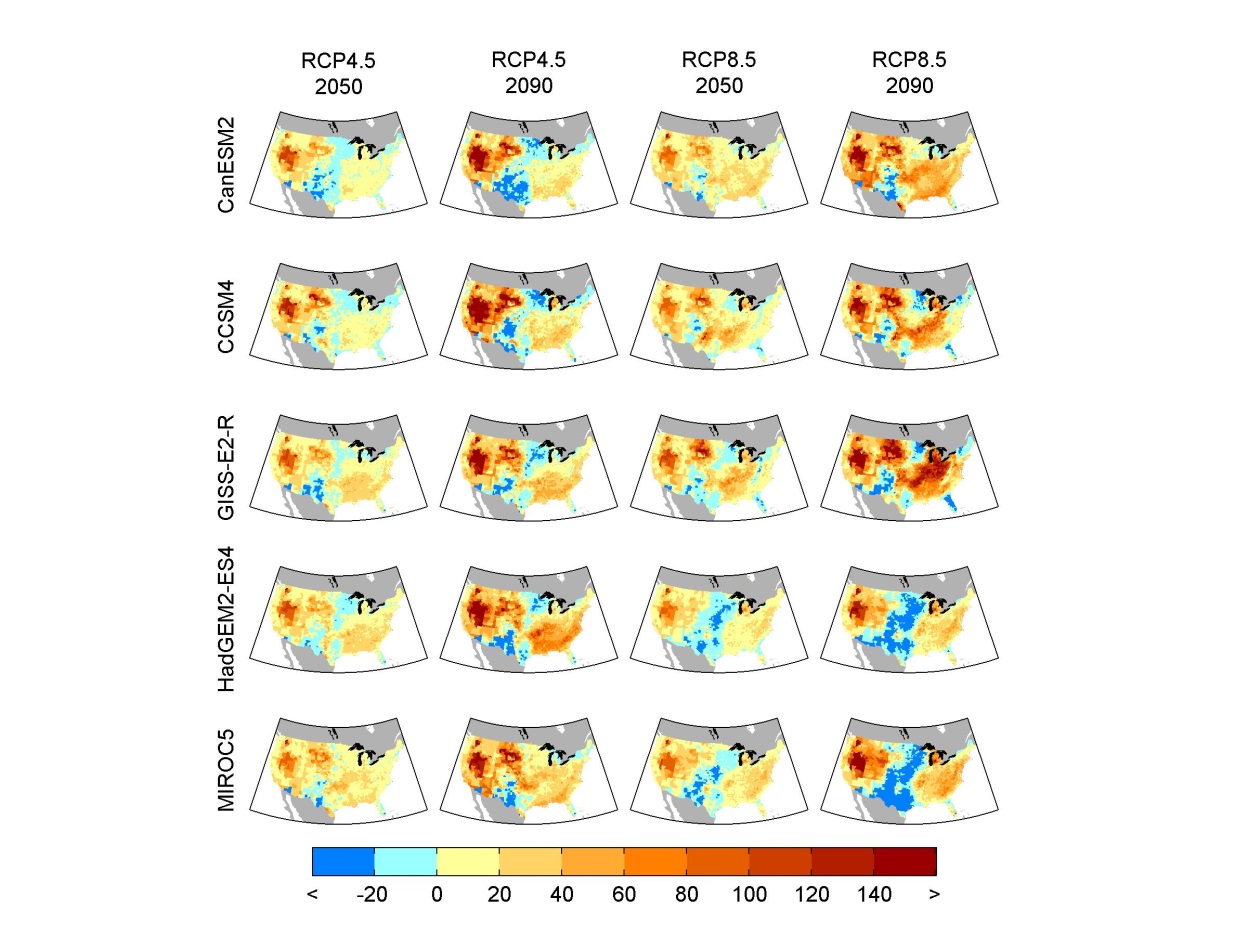


Figure 10: Percent change in live, above-ground tree carbon from the baseline climate era (1986-2005) to the 2050 era (2040-2059 average) and the 2090 era (2080-2099 average) for each GCM and RCP combination at the county level.


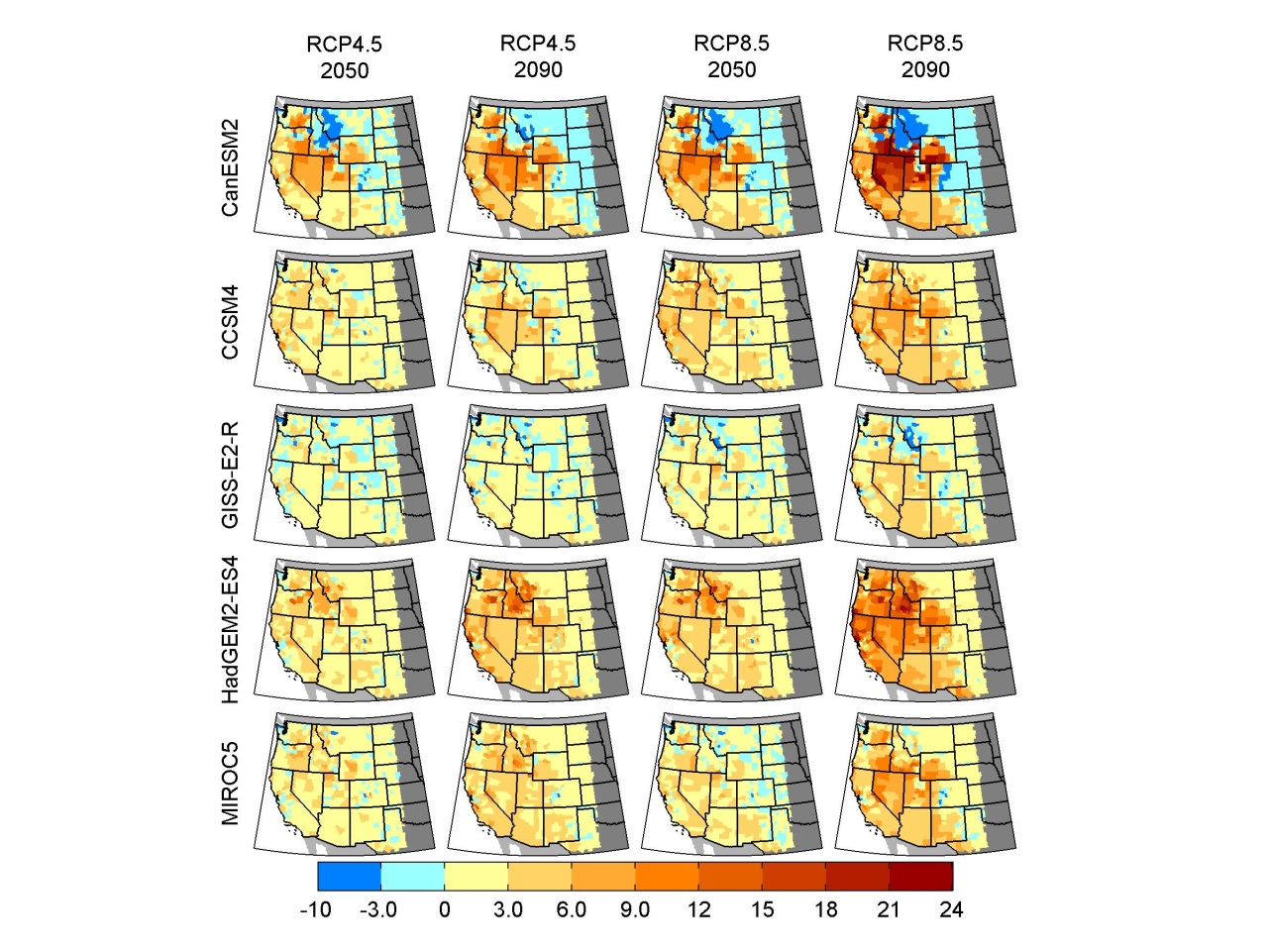


Figure 11: Absolute change in hundreds of hectares burned from wildfires from the baseline climate era (1986-2005) to the 2050 era (2040-2059 average) and the 2090 era (2080-2098* average) for each GCM and RCP combination at the county level.

## S.18 Maps of Stressor-Response Results


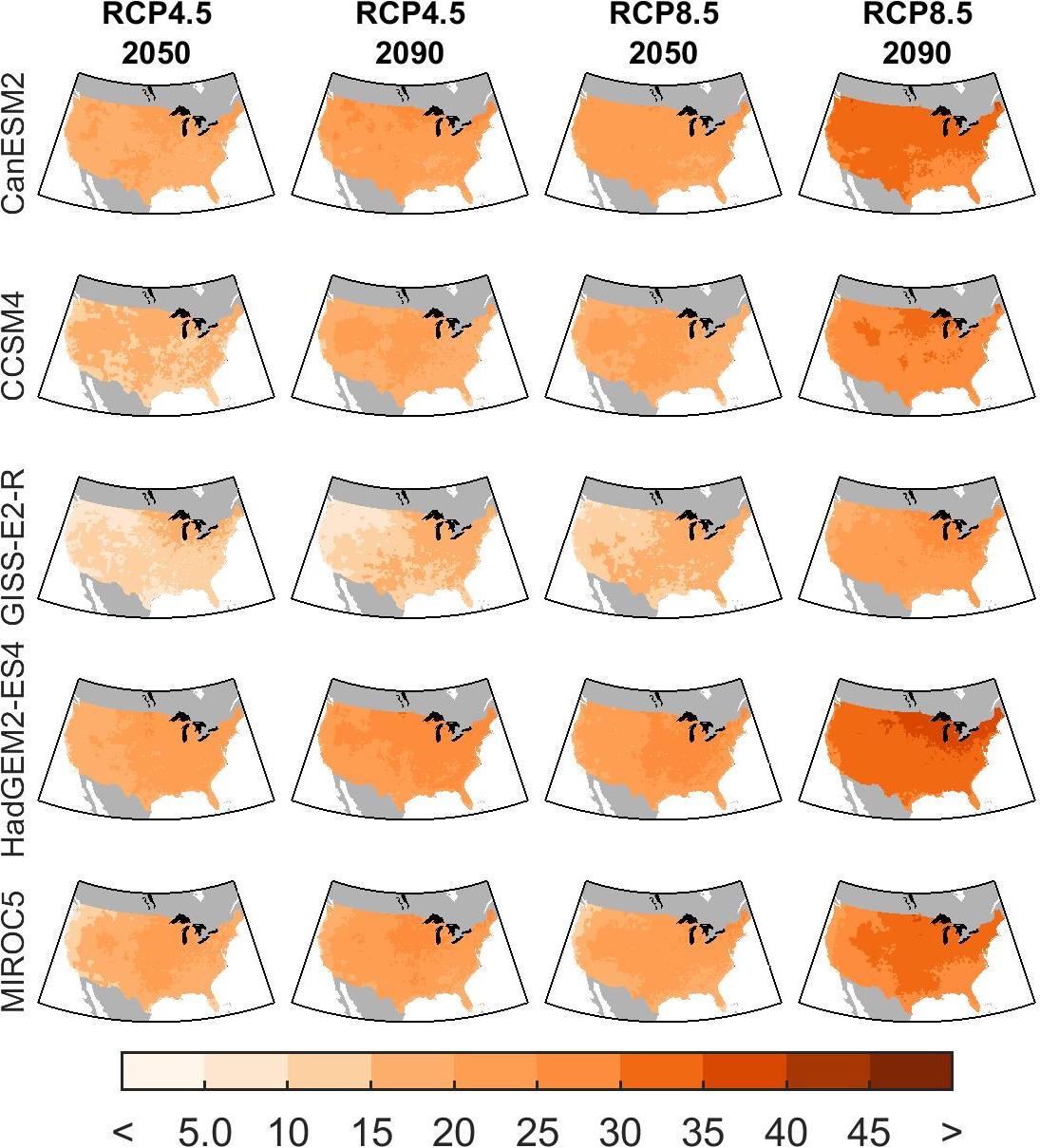


Figure 12: Percent reduction in substation transformer lifespan from the baseline climate era (1986-2005) to the 2050 era (2040-2059 average) and the 2090 era (2080-2099 average) for each GCM and RCP combination under the No Adaptation scenario. Reduction shown at the county level.


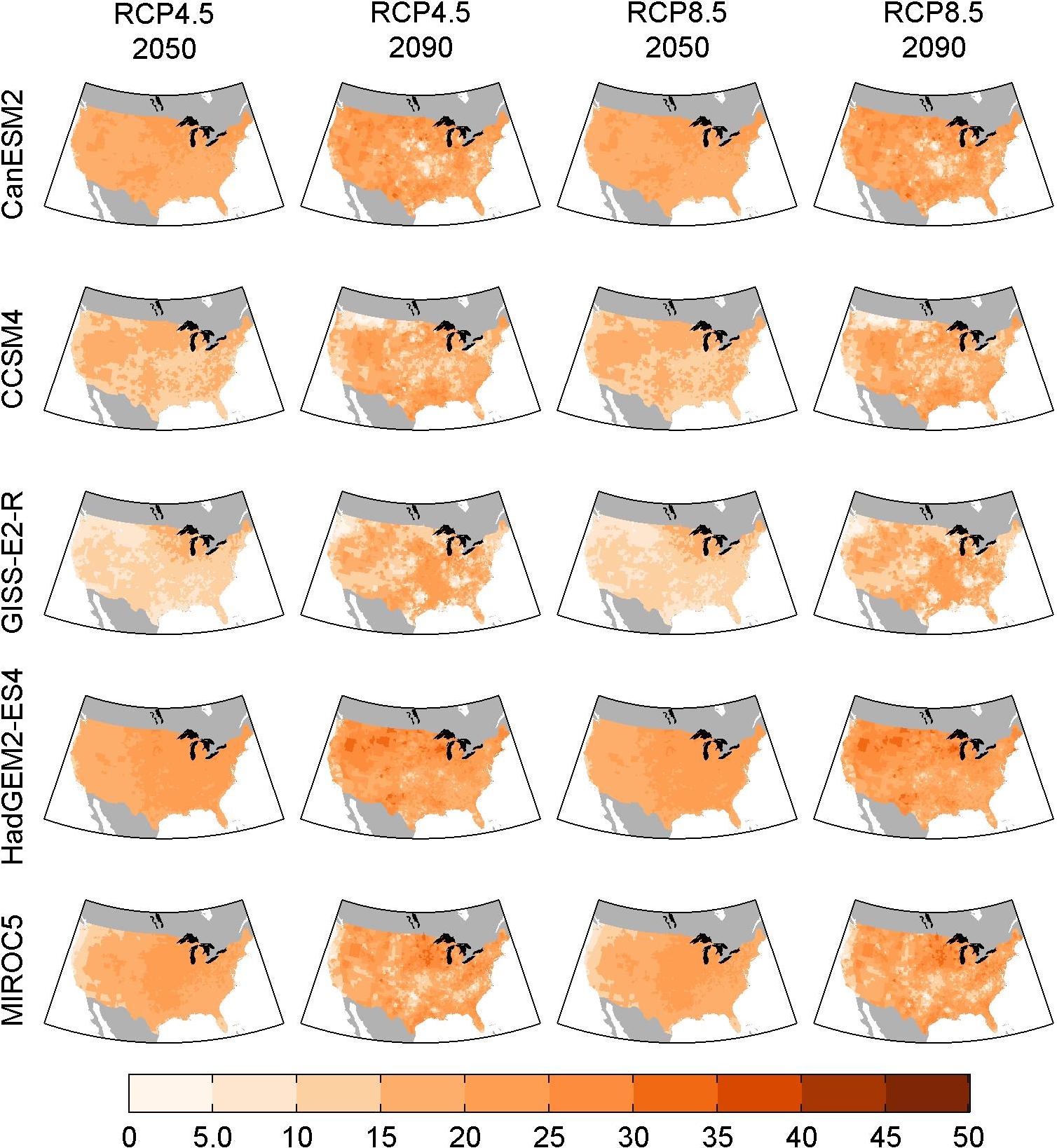


Figure 13: Percent reduction in substation transformer lifespan from the baseline climate era (1986-2005) to the 2050 era (2040-2059 average) and the 2090 era (2080-2099 average) for each GCM and RCP combination under the Reactive Adaptation scenario. Reduction shown at the county level.


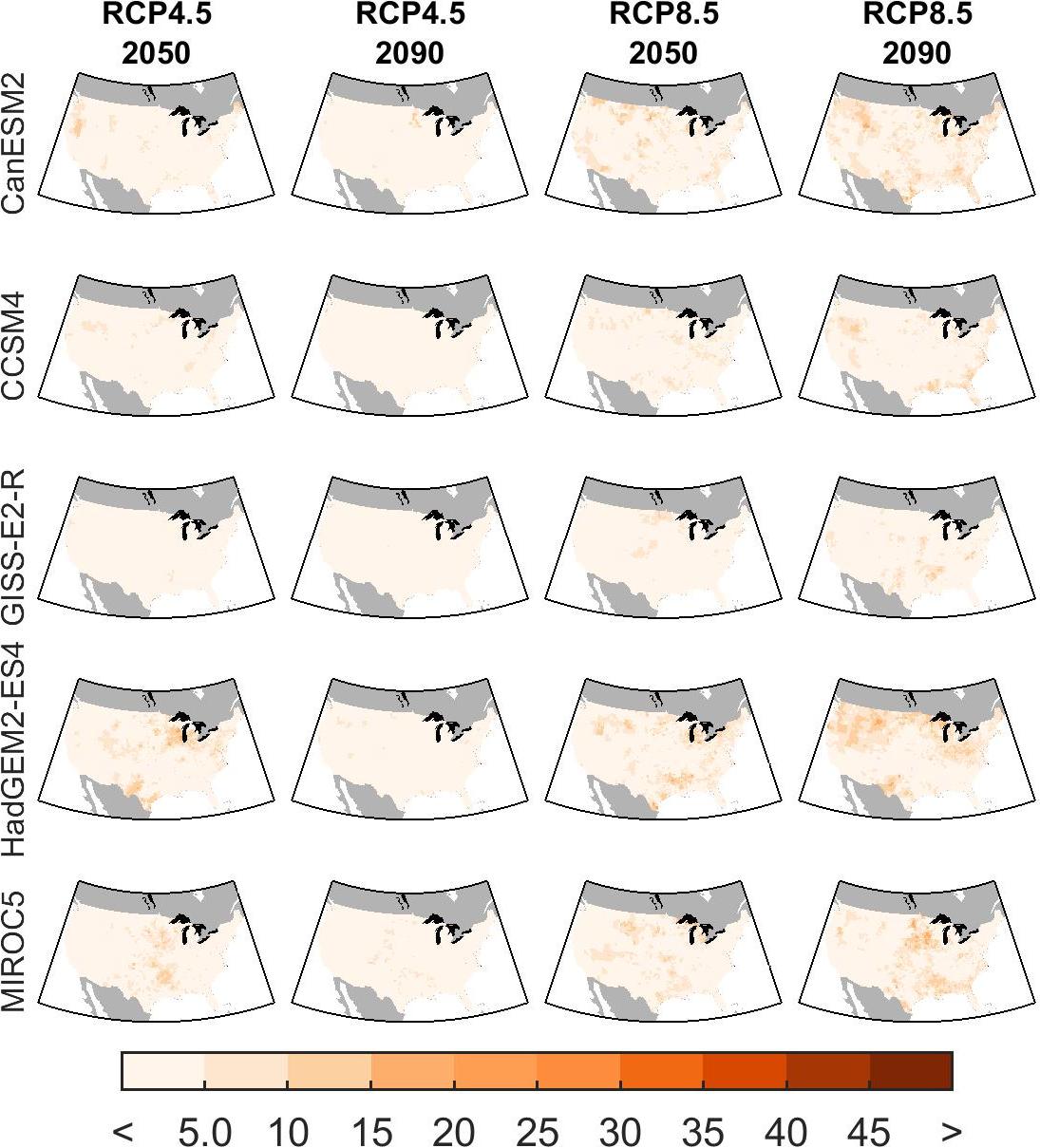


Figure 14: Percent reduction in substation transformer lifespan from the baseline climate era (1986-2005) to the 2050 era (2040-2059 average) and the 2090 era (2080-2099 average) for each GCM and RCP combination under the Proactive adaptation scenario. Reduction shown at the county level.


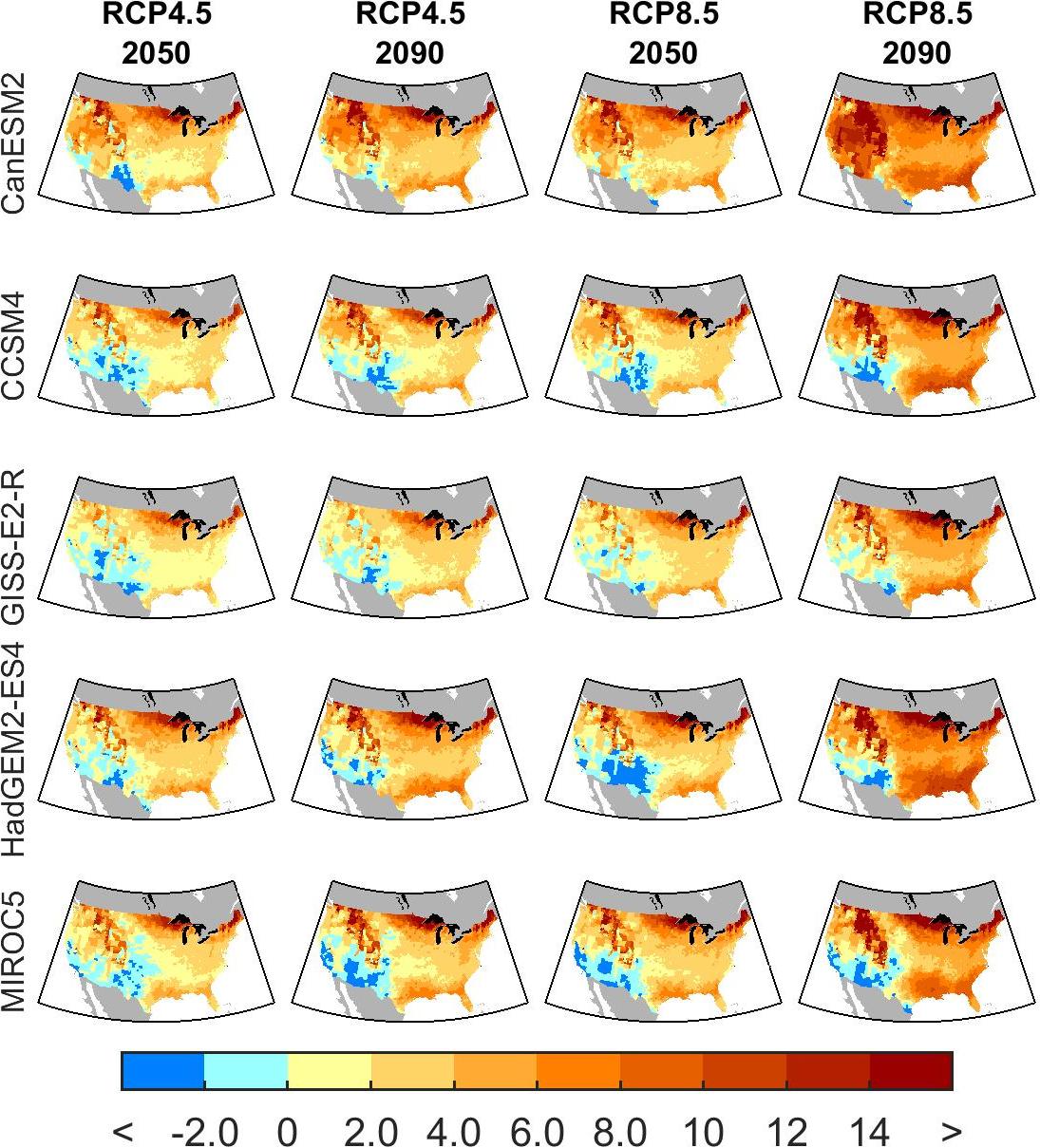


Figure 15: Percent reduction in wood pole lifespan from the baseline climate era (1986-2005) to the 2050 era (2040-2059 average) and the 2090 era (2080-2099 average) for each GCM and RCP combination under the No Adaptation scenario. Reduction shown at the county level.


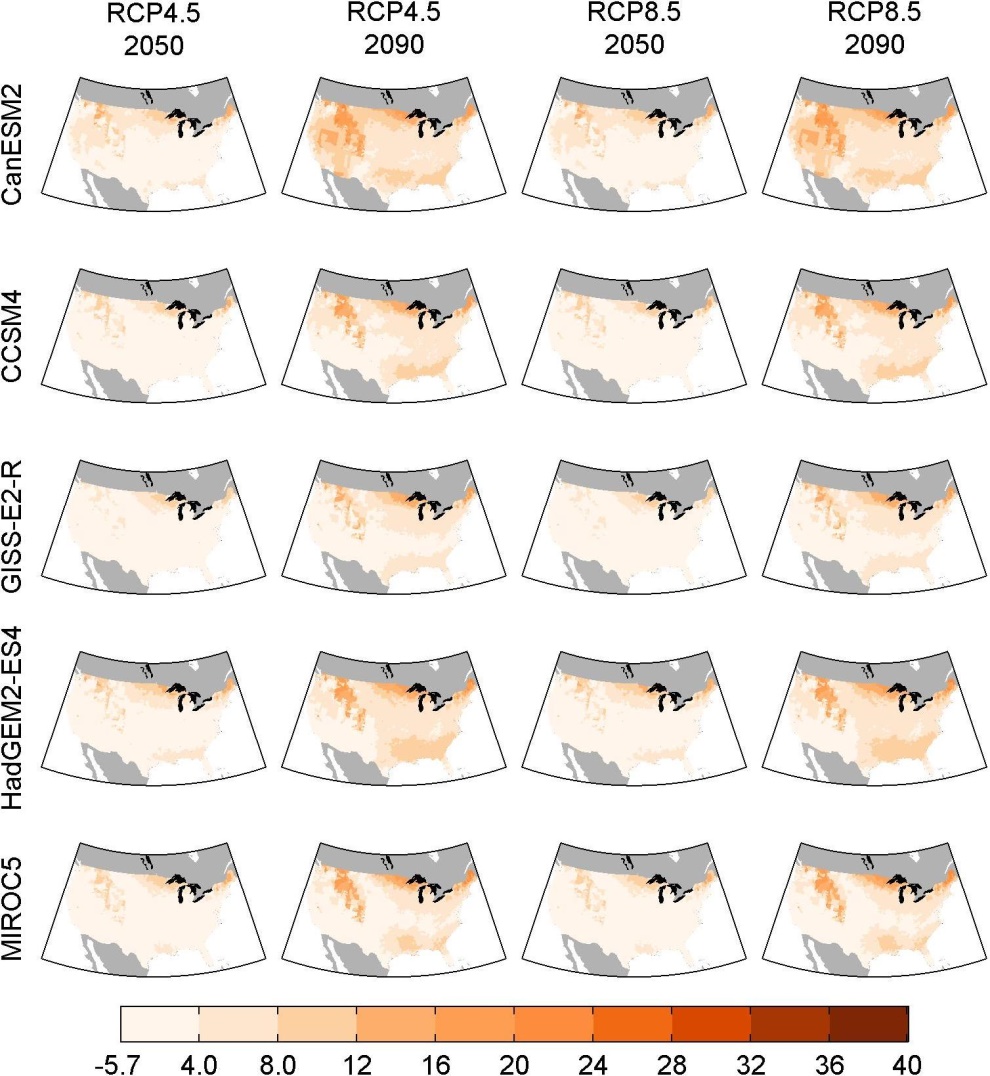


Figure 16: Percent reduction in wood pole lifespan from the baseline climate era (1986-2005) to the 2050 era (2040-2059 average) and the 2090 era (2080-2099 average) for each GCM and RCP combination under the Reactive adaptation scenario. Reduction shown at the county level.


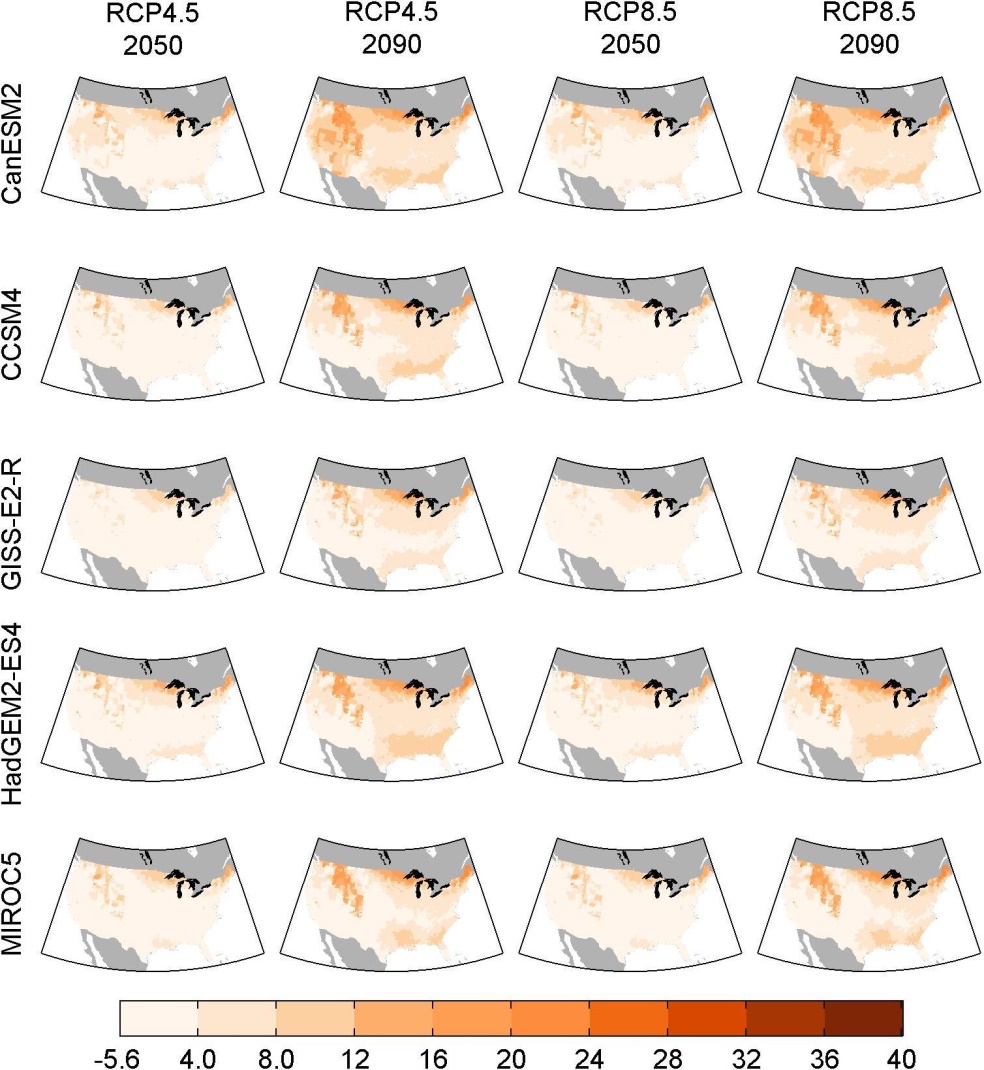


Figure 17: Percent reduction in wood pole lifespan from the baseline climate era (1986-2005) to the 2050 era (2040-2059 average) and the 2090 era (2080-2099 average) for each GCM and RCP combination under the Proactive adaptation scenario. Reduction shown at the county level.


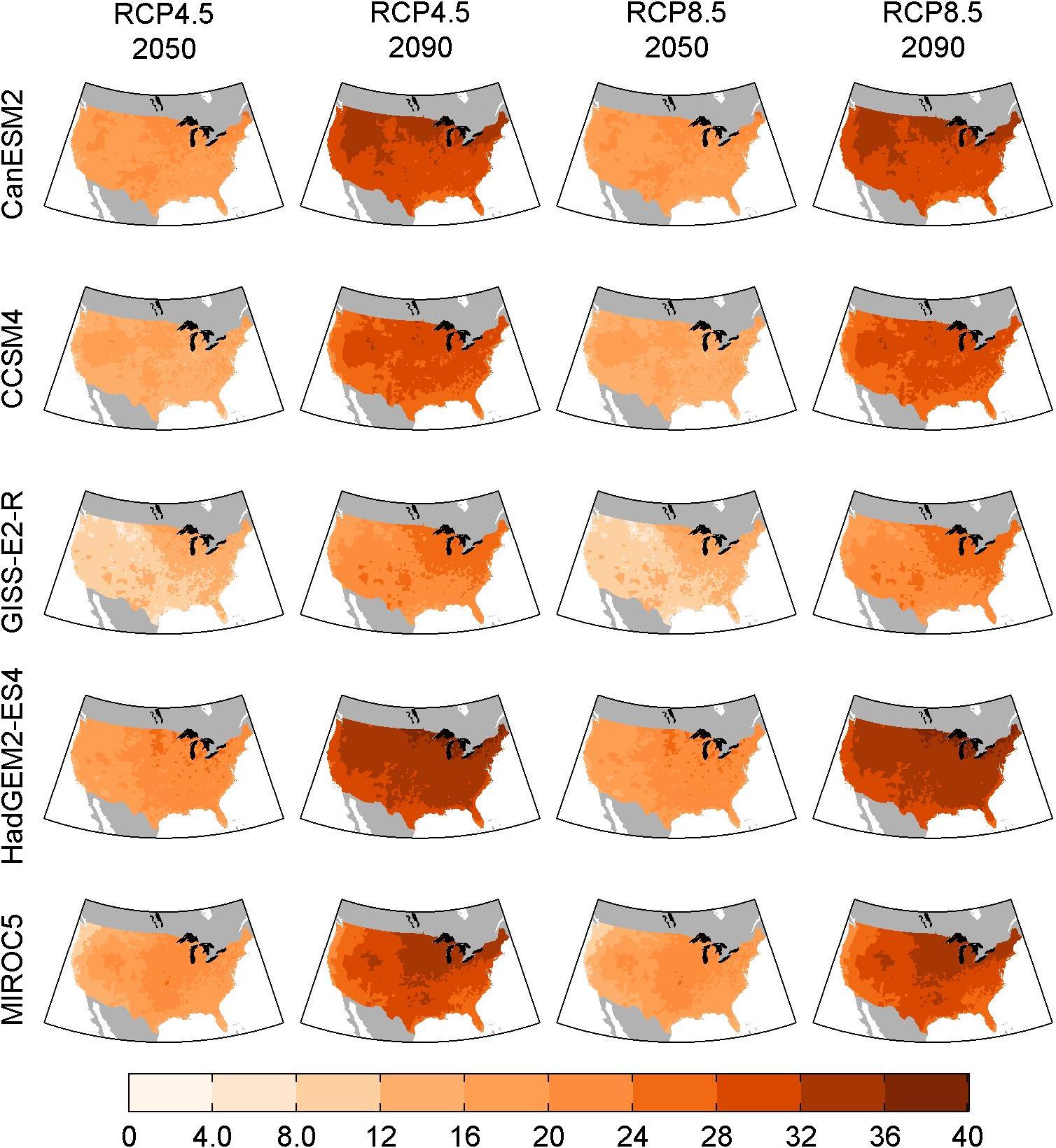


Figure 18: Percent reduction in distribution transformer lifespan from the baseline climate era (1986-2005) to the 2050 era (2040-2059 average) and the 2090 era (2080-2099 average) for each GCM and RCP combination under the No Adaptation scenario. Reduction shown at the county level.


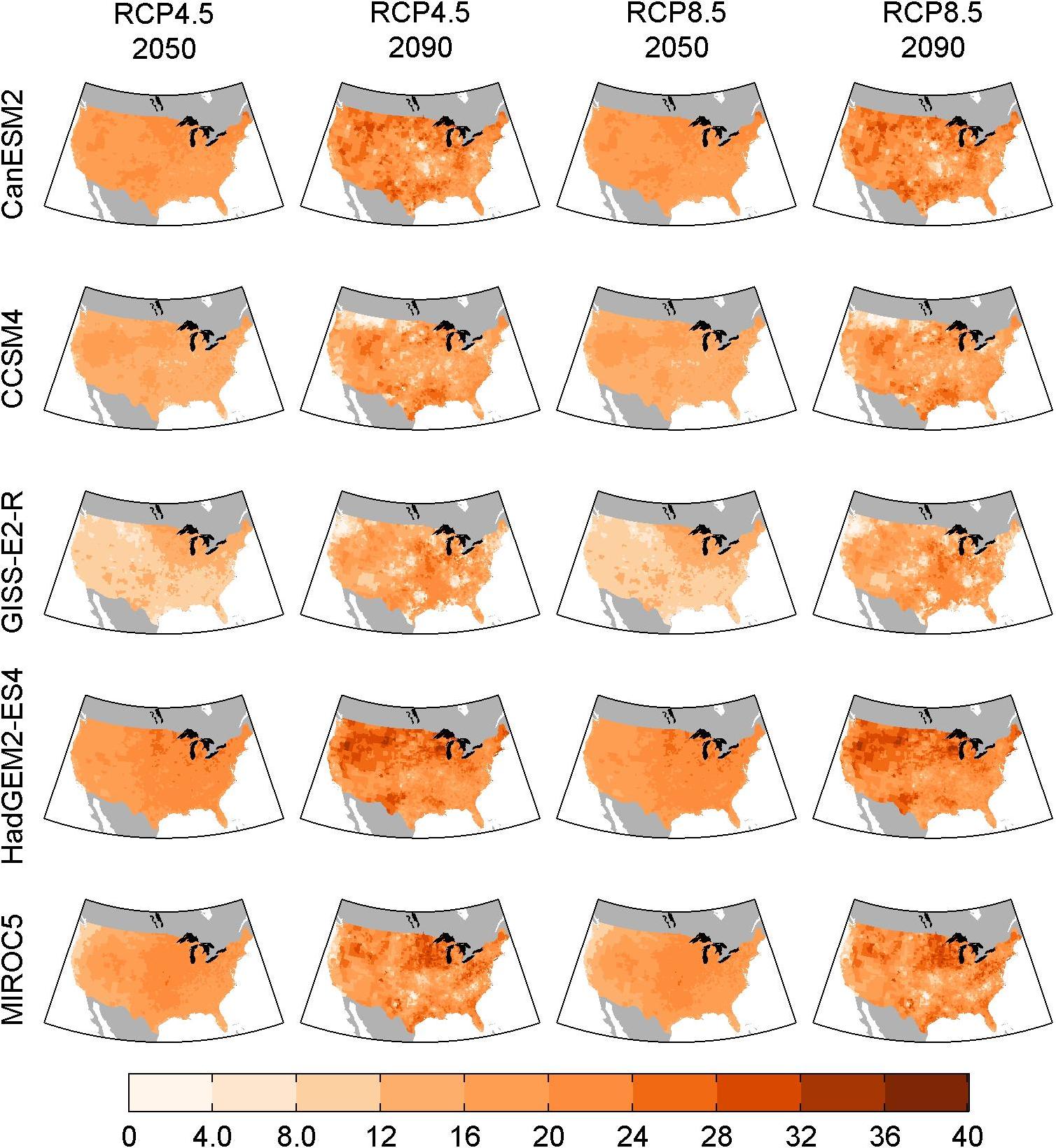


Figure 19: Percent reduction in distribution transformer lifespan from the baseline climate era (1986-2005) to the 2050 era (2040-2059 average) and the 2090 era (2080-2099 average) for each GCM and RCP combination under the Reactive adaptation scenario. Reduction shown at the county level.


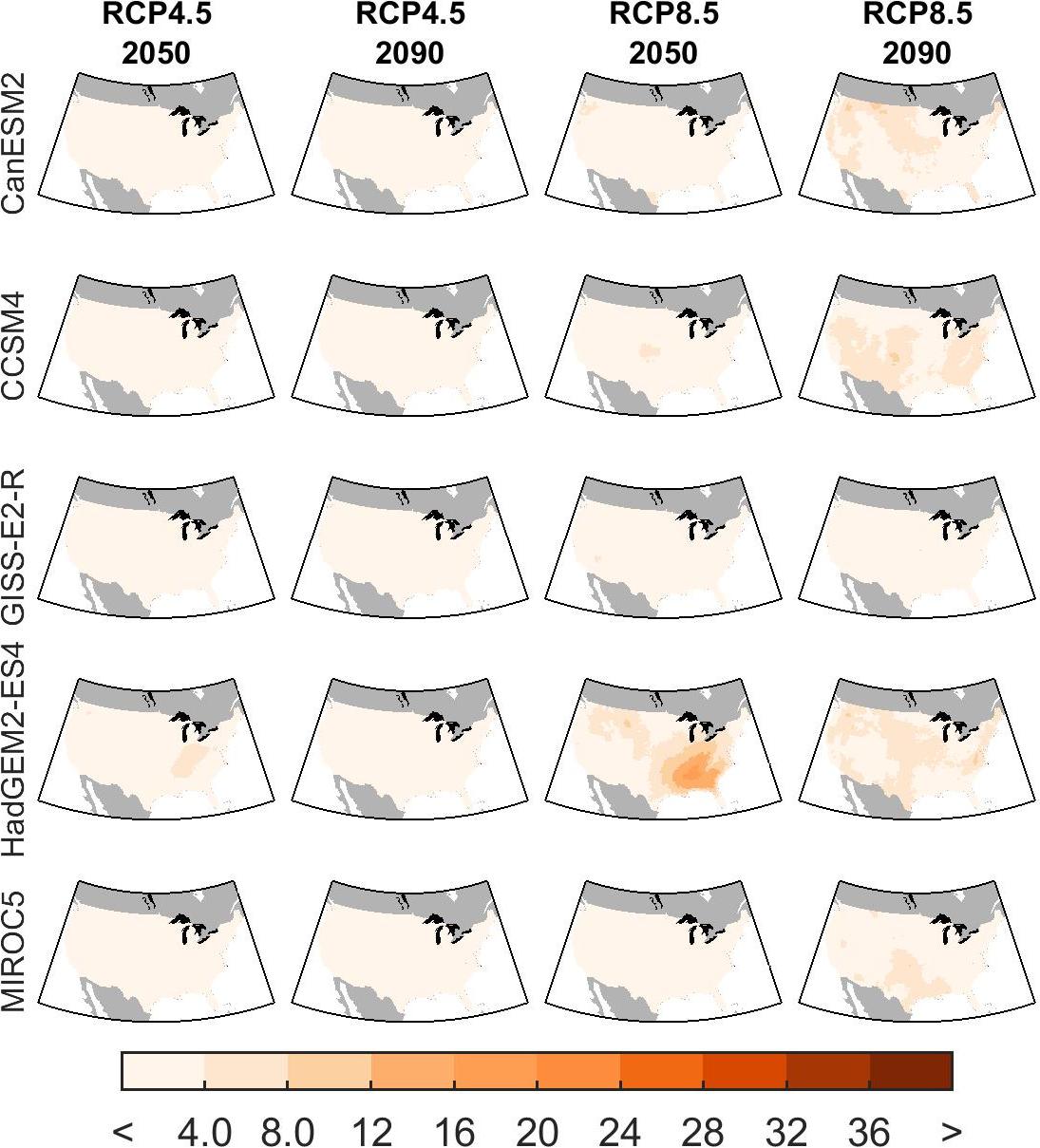


Figure 20: Percent reduction in distribution transformer lifespan from the baseline climate era (1986-2005) to the 2050 era (2040-2059 average) and the 2090 era (2080-2099 average) for each GCM and RCP combination under the proactive adaptation scenario. Reduction shown at the county level.


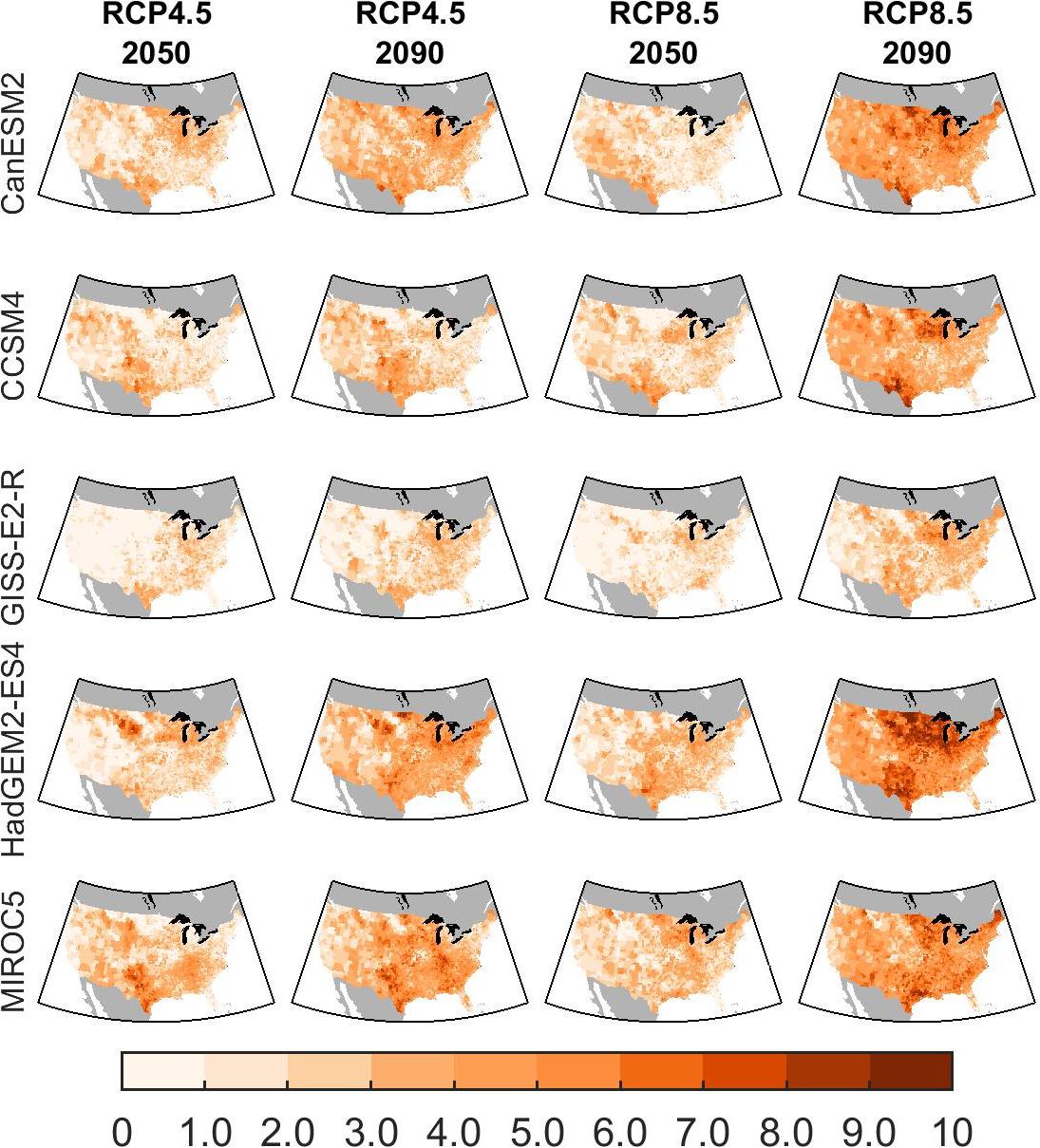


Figure 21: Percent increase in transmission line capacity (in response to reduced line ampacity from rising ambient temperatures) from the baseline climate era (1986-2005) to the 2050 era (2040-2059 average) and the 2090 era (2080-2099 average) for each GCM and RCP combination under the No Adaptation scenario. Reduction shown at the county level.


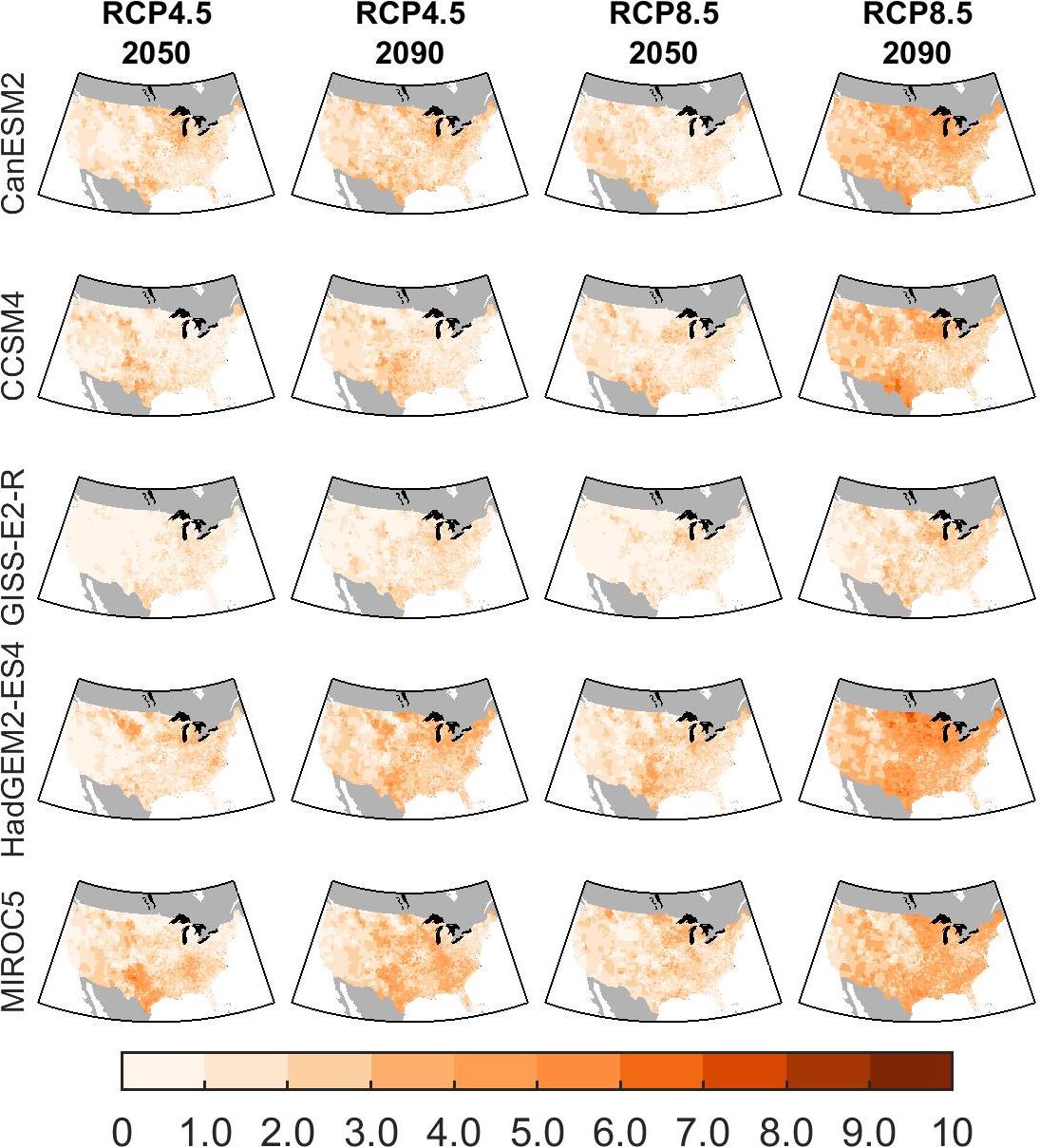


Figure 22: Percent increase transmission line capacity from the baseline climate era (1986-2005) to the 2050 era (2040-2059 average) and the 2090 era (2080-2099 average) for each GCM and RCP combination under the Reactive adaptation scenario. Reduction shown at the county level.


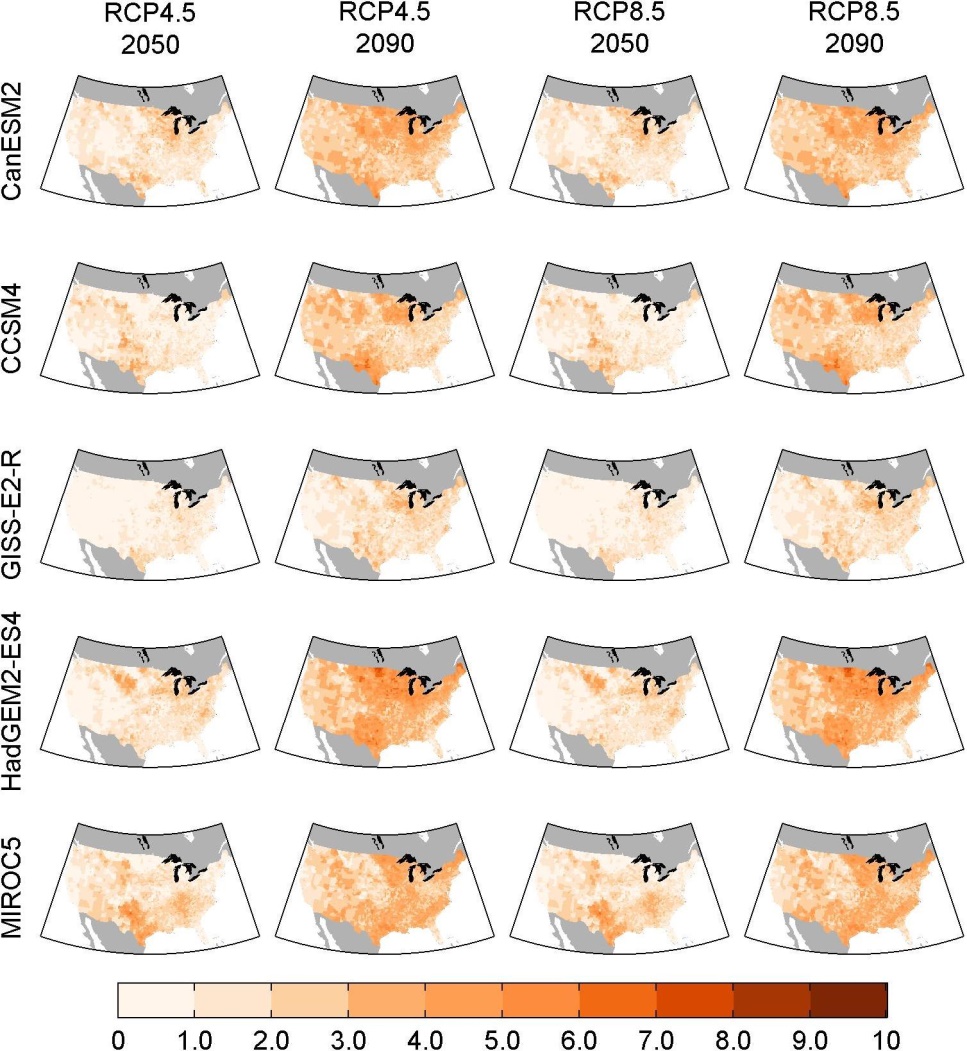


Figure 23: Percent increase transmission line capacity from the baseline climate era (1986-2005) to the 2050 era (2040-2059 average) and the 2090 era (2080-2099 average) for each GCM and RCP combination under the proactive adaptation scenario. Reduction shown at the county level.


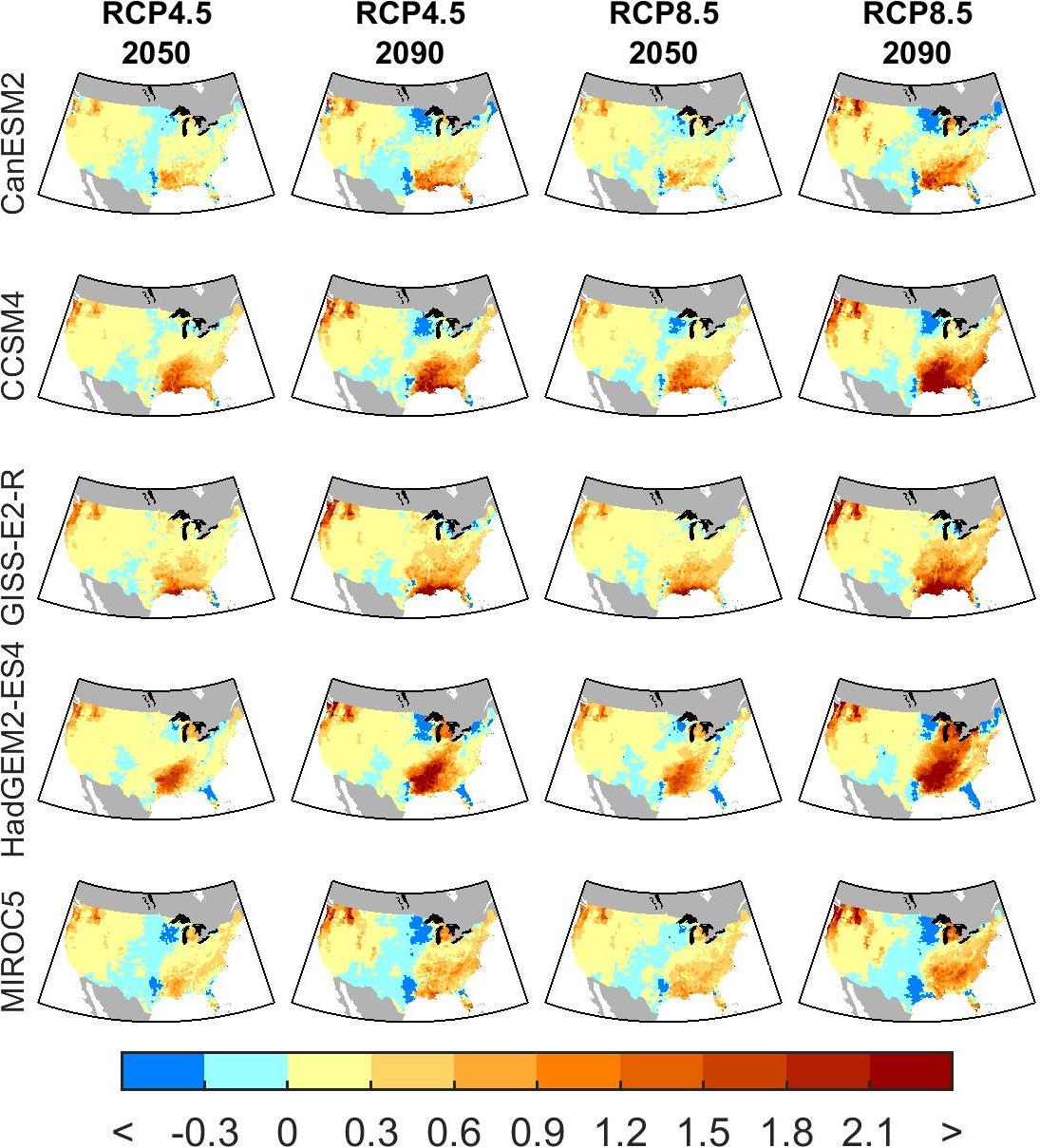


Figure 24: Change in tree trimming cost in thousand $USD per distribution line mile from baseline climate era (1986-2005) to the 2050 era (2040-2059 average) and the 2090 era (2080-2099 average) for each GCM and RCP combination under the no adaptation, reactive, and proactive scenarios. Reduction shown at the county level.

## S.17 Map of Total Costs


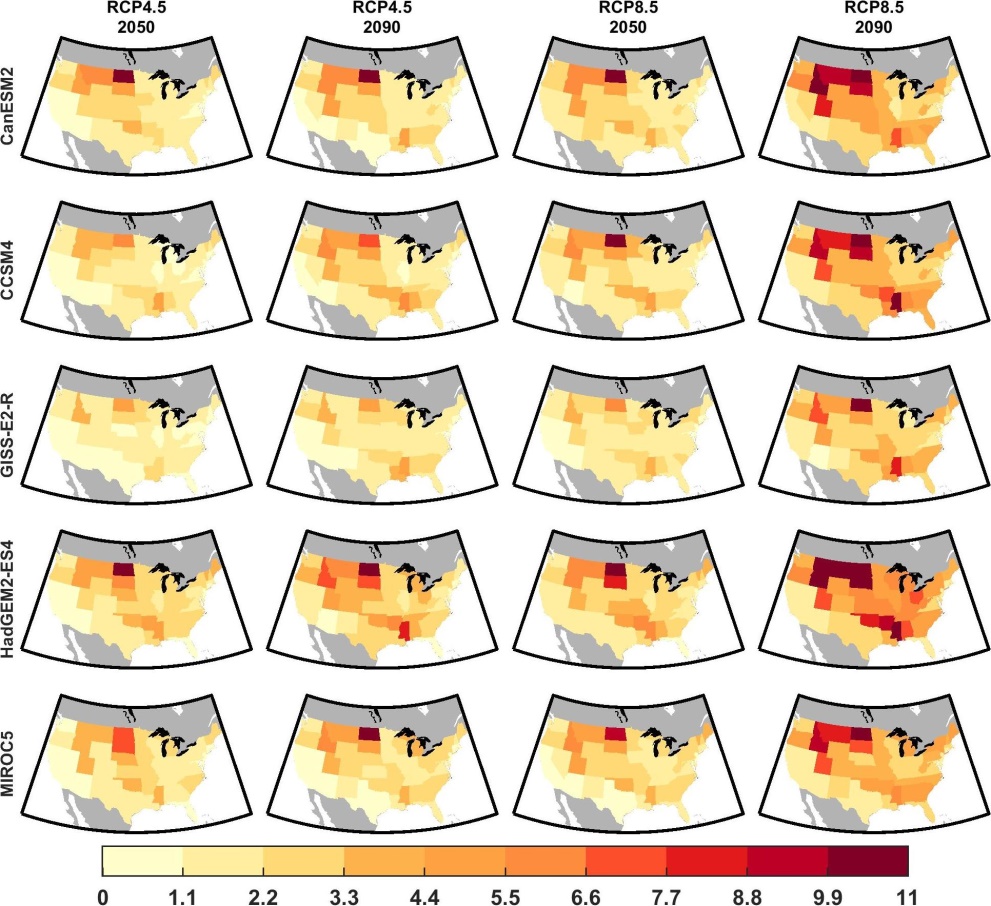


Figure 25: Change in total costs from baseline (climate from 1986-2005 and projected infrastructure) to the 2050 era (2040-2059 average) and the 2090 era (2080-2099 average) for each GCM and RCP combination under the No Adaptation scenario, shown at the county level.


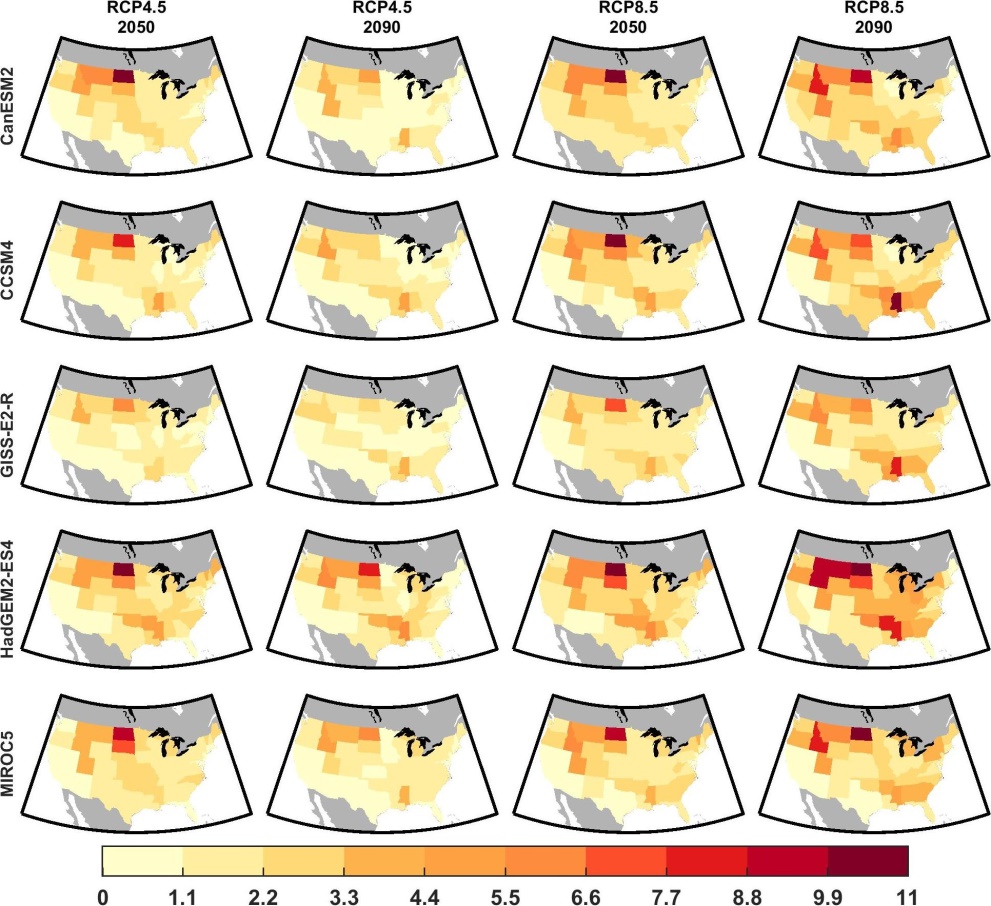


Figure 26: Change in total costs from baseline (climate from 1986-2005 and projected infrastructure) to the 2050 era (2040-2059 average) and the 2090 era (2080-2099 average) for each GCM and RCP combination under the Reactive scenario, shown at the county level.


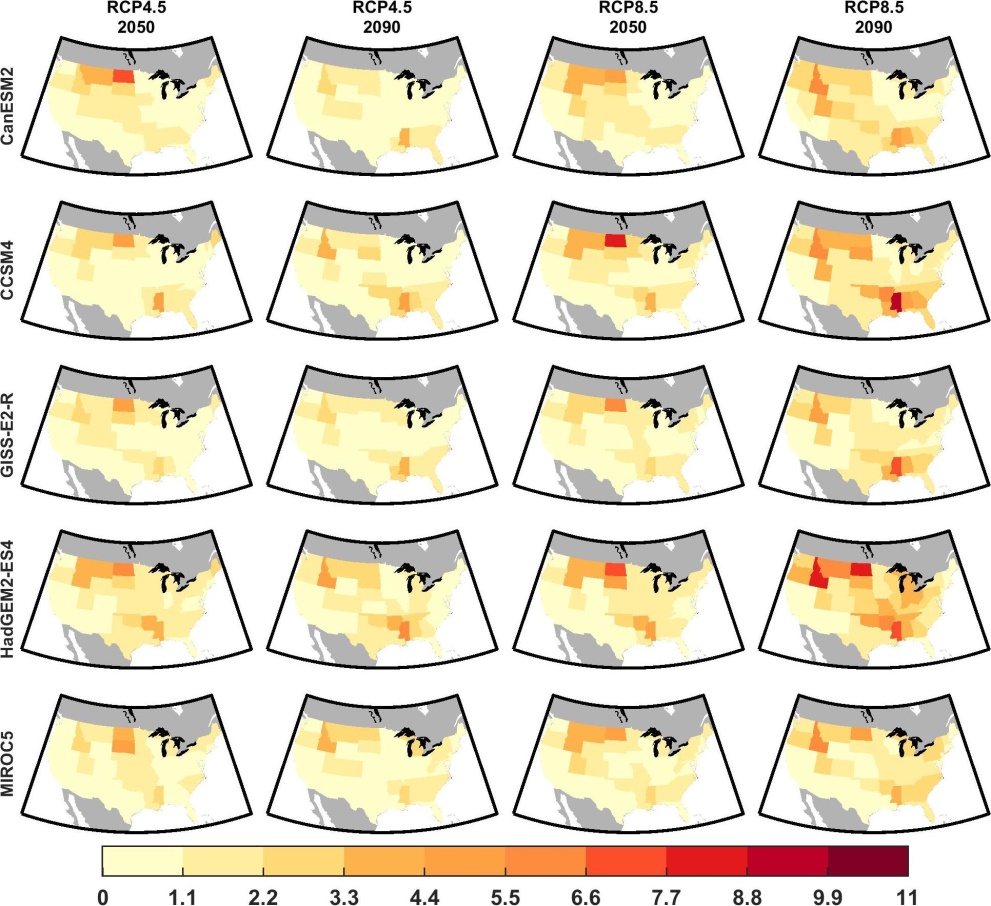


Figure 27: Change in total costs from baseline (climate from 1986-2005 and projected infrastructure) to the 2050 era (2040-2059 average) and the 2090 era (2080-2099 average) for each GCM and RCP combination under the Proactive scenario, shown at the county level.

**References**

Alvehag K, Soder L. A reliability model for distribution systems incorporating seasonal variations in severe weather. IEEE Trans Power Delivery 2011; 26(2):910–9.

Ausgrid. 2015. NS167 Positioning Of Poles and Lighting Columns. Ausgrid.

Backer, L.C.; Manassaram-Baptiste, D.; LePrell, R.; Bolton, B. (2015) Cyanobacteria and algae Blooms: review of health and environmental data from the harmful algal bloom-related illness surveillance system (HABISS) 2007–2011. Toxins, 7(4):1048-1064.

Bartos M, Chester M, Johnson N, Gorman B, Eisenberg D, Linkov I, Bases M. (2016) Impacts of rising air temperature of electric transmission ampacity and peak electricity load in the United States. Environ. Res. Lett. 11 (2016) 114008. doi:10.1088/1748-9326/11/11/114008.

Billinton, R., R. N. Allan, Reliability Evaluation of Power Systems, 2nd Edition, Plenum Press, New York, 1996.

Bjarnadottir, S., Li, Y. and Stewart, M.G. (2012) Hurricane Risk Assessment of Power Distribution Poles Considering Impacts of A Changing Climate, Journal of Infrastructure Systems.

Bloniarz, D.V., 1992. Street Trees, Overhead Utility Distribution, and Physical Infrastructure: Design Implications, Maintenance Costs and Proposed Alternatives. Northeast Center for Urban & Community Forestry, USDA Forest Service.

Broström, E. and Söder, L., 2007. Ice storm impact on power system reliability. In 12 th International Workshop on Atmospheric Icing on Structures (IWAIS 2007), Yokohama, Japan.

Brown, R.E. (2008) Electric Power Distribution Reliability. CRC Press: Boca Raton, FL.

Campbell, R. J. (2012) “Weather‐Related Power Outages and Electric System Resiliency.” Congressional Research Service Report for Congress. 7‐5700 R42696. www.crs.gov.

Changnon, Stanley A. (2007) Catastrophic winter storms: An escalating problem. Climatic Change (2007) 84: 131. https://doi.org/10.1007/s10584-007-9289-5

Cheng, Chad Shouquan, Guilong Li & Heather Auld (2011) Possible Impacts of Climate Change on Freezing Rain Using Downscaled Future Climate Scenarios: Updated for Eastern Canada, Atmosphere-Ocean, 49:1, 8-21, DOI: 10.1080/07055900.2011.555728

Coder, Kim D. (2015) Historic Ice Storm Patterns. Warnell School of Forestry & Natural Resources, University of Georgia, WSFNR15-1

Collins WJ, Bellouin N, Doutriaux-Boucher M, Gedney N, Halloran P, Hinton T, Hughes J, Jones CD, Joshi M, Liddicoat S, Martin G. 2011. Development and evaluation of an Earthsystem model–HadGEM2. Geoscience Model Develeopment, 4:1051-1075.

Cunningham, E.L., 1987. Firm price tree trimming at Jersey Central Power and Light. Journal of arboriculture (USA).

Davidson, R. A., Liu, H. I., Sarpong, K., Sparks, P., and Rosowsky, D. V. (2003) Electric power distribution system performance in Carolina hurricanes, Natural Hazards Review, 4, 36-45.

Davies T, Cullen MJ, Malcolm AJ, Mawson MH, Staniforth A, White AA, Wood N. 2005. A new dynamical core for the Met Office's global and regional modelling of the atmosphere. Quarterly Journal of the Royal Meteorological Society, 131:1759-1782.

Department of Energy, 2015. Quadrennial Energy Review First Installment: Transforming U.S. Energy Infrastructures In A Time Of Rapid Change.

DOE (2006) Benefits of Using Mobile Transformers and Mobile Substations for Rapidly Restoring Electrical Service, a report to the United States Congress pursuant to Section 1816 of the Energy Policy Act of 2005, U.S. Department of Energy. <http://energy.gov/sites/prod/files/oeprod/DocumentsandMedia/MTS_Report_to_Congress_FINAL_73106.pdf>

Drapek, R.J., J.B. Kim, and R.P Neilson, (2015) Continent-wide Simulations of a Dynamic Global Vegetation Model over the United States and Canada under Nine AR4 Future Scenarios. Global Vegetation Dynamics: Concepts and Applications in the MC1 Model, 73-90.

É. Bresson, R. Laprise, D. Paquin, J.M. Thériault & R. de Elía (2017) Evaluating the Ability of CRCM5 to Simulate Mixed Precipitation, Atmosphere-Ocean, 55:2, 79-93, DOI: 10.1080/07055900.2017.1310084

Emanuel, K. A. (2013). Downscaling CMIP5 climate models shows increased tropical cyclone activity over the 21st century. Proceedings of the National Academy of Sciences, 110, 12219-12224.

Endeavour Energy. 2014. Electricity supply to rural areas.

Energex. 2013. Supply & Planning Manual. Energex.

EPA. (2017). Integrated Climate and Land-Use Scenarios. [online] Available at: https://www.epa.gov/iclus [Accessed 13 May 2018].

EPA. 2017. Multi-Model Framework for Quantitative Sectoral Impacts Analysis: A Technical Report for the Fourth National Climate Assessment. U.S. Environmental Protection Agency, EPA 430-R-17-001.

Eto, J. E., K. H. LaCommare, P. Larsen, A. Todd, and E. Fisher. (2012) An Examination of Temporal Trends in Electricity Reliability Based on Reports from U.S. Electric Utilities. Berkeley CA: Lawrence Berkeley National Laboratory Report LBNL‐5268E. January. http://certs.lbl.gov/certs‐rtinapubs.html

Federal Energy Regulatory Commission (2015). Form No. 714—Annual Electric Balancing Authority Area and Planning Area Report, 1993-2013 (Data set). Retrieved May 2015 from (http://ferc.gov/docs-filing/forms/form-714/data.asp)

FERC (2018). Form 1 - Electric Utility Annual Report. [online] Available at: https://www.ferc.gov/docs-filing/forms/form-1/viewer-instruct.asp [Accessed 24 Mar. 2018].

Gent PR, Danabasoglu G, Donner LJ, Holland MM, Hunke E, Jayne S, Lawrence D, Neale RB, Rasch PJ, Vertenstein M, Worley PH. 2011. The community climate system model version 4. Journal of Climate, 24:4973-4991.

Guikema, S.D., Quiring, S.M. and Han, S.R., 2010. Prestorm estimation of hurricane damage to electric power distribution systems. Risk Analysis: An International Journal, 30(12), pp.1744-1752.

Hoff, T. and D. S. Shugar (no date). The Value Of Grid-Support Photovoltaics to Substation Transformers.

Homeland Infrastructure Foundation-Level Data. (2017). HIFLD Open Data. [online] Available at: https://hifld-geoplatform.opendata.arcgis.com/ [Accessed 26 Mar. 2018].

Huffman, G. J., and G. A. Norman Jr., 1988: The supercooled warm rain process and the specification of freezing precipitation. Mon. Wea. Rev., 116, 2172–2182.

IPCC D2013] In: Stocker TF et al Deds] Climate change 2013: The physical science basis. Contribution of Working Group I to the Fifth Assessment Report of the Intergovernmental Panel on Climate change. Cambridge University Press, Cambridge, 1535 pp

Johnson, Jesse M. (2014) Quantifying the Economic Risk of Wildfires and Power Lines in San Diego County. Master’s Project. Duke University.

Jungo P, Goyette S, Beniston M. 2002. Daily wind gust speed probabilities over Switzerland according to three types of synoptic circulation. International Journal of Climatology 22: 485–499.

Kezunovic, M., T. Dokic, (2017) “Predictive Asset Management Under Weather Impacts Using Big Data, Spatiotemporal Data Analytics and Risk Based Decision-Making,” 10th Bulk Power Systems Dynamics and Control Symposium – IREP’2017, Espinho, Portugal, August 2017.

Kezunovic, M., Z. Obradovic, T. Dokic, S. Roychoudhury, (2018) “Systematic Framework for Integration of Weather Data into Prediction Models for the Electric Grid Outage and Asset Management Applications,” The Hawaii International Conference on System Sciences – HICSS, Waikoloa Village, Hawaii, January 2018.

Klima, K. & Morgan, M.G. (2015). Ice Storm Frequencies in a Warmer Climate. Climatic Change (2015) 133: 209. https://doi.org/10.1007/s10584-015-1460-9

Kovacik C A (2014) Preliminary climatology of U.S. ice storm frequency and a comparison between Northeast U.S. ice storm frequency and Teleconnections. M.S. Thesis, University of Oklahoma

Kulkarni, Sujay and Huei-Ping Huang (2014). Changes in Surface Wind Speed over North America from CMIP5 Model Projections and Implications for Wind Energy. Advances in Meteorology Volume 2014, Article ID 292768, http://dx.doi.org/10.1155/2014/292768

LaCommare, Kristina Hamachi, and Joseph H. Eto (2005). Cost of Power Interruptions to Electricity Consumers in the United States. Energy, Vol. 31. Elsevier: April 7, 2005.

Larsen, P. (2016). A Method to Estimate the Costs and Benefits of Undergrounding Electricity Transmission and Distribution Lines. Energy Economics, 60, 47-61.

Larsen, P., O. S. Goldsmith, O. Smith, M. Wilson, K. Strzepek, P. Chinowsky, and B. Saylor. (2008). Estimating the Future Costs of Alaska Public Infrastructure at Risk to Climate Change. Global Environmental Change, Elsevier Press: East Anglia.

Larsen, P.H., Boehlert, B., Eto, J., Hamachi-LaCommare, K., Martinich, J. and Rennels, L., (2018). Projecting future costs to US electric utility customers from power interruptions. Energy, 147, pp.1256-1277.

Larsen, P.H., LaCommare, K.H., Eto, J.H. and Sweeney, J.L., (2015). Assessing changes in the reliability of the US electric power system.

Larsen, Peter H., Brent Boehlert, Joseph H. Eto, Kristina Hamachi-LaCommare, Jeremy Martinich, Lisa Rennels. (2017) Projecting Future Costs to U.S. Electric Utility Customers from Power Interruptions. Lawrence Berkeley National Laboratory. LBNL-1007027

Li Y, Ellingwood BR. Hurricane damage to residential construction in the US: Importance of uncertainty modeling in risk assessment. Engineering Structures 2006; 28:1009-1017.

Li, X., and G. Zielke. 2003. “A Study on Transformer Loading in Manitoba-Peak Load Ambient Temperature.” IEEE Transactions on Power Delivery 18(4): 1249–1256.

Li, X., R. Mazur, D. Allen, and D. Swatek. 2005. Specifying Transformer Winter and Summer Peak-Load Limits. IEEE Transactions on Power Delivery 20(1): 185–190.

Lundgaard, L.E., W. Hansen, D. Linhjell & T.J. Painter (2004). Aging of oil-impregnated paper in power transformers. IEEE Transactions on Power Delivery, 19(1): 230–239. doi:10.1109/TPWRD.2003.820175.

Mauldin, P. (2014). Storm Hardening the Grid. [online] Transmission & Distribution World. Available at: https://www.tdworld.com/distribution/storm-hardening-grid [Accessed 3 Aug. 2018].

McFarland Cascade. (2018). Standard Dimensions for Douglas Fir and Southern Yellow Pine Poles. [online] Available at: http://www.ldm.com/docs/dimensiontables_df_sp.pdf [Accessed 18 May 2018].

McFarland, J., Y. Zhou, L. Clarke, P. Sullivan, J. Colman, W. Jaglom, M. Colley, P. Patel, J. Eom, S. Kim, G. Kyle, P. Schultz, B Venkatesh, J. Haydel, C. Mack, and J. Creason, 2015: Impacts of rising air temperatures and emissions mitigation on electricity demand and supply in the United States: a multi-model comparison. Climatic Change, doi: 10.1007/s10584-015-1380-8.

Meliopoulos, A. P. (2006) Lightning and Overvoltage Protection. Standard Handbook for Electrical Engineers, Section 27. McGraw-Hill.

Most, W.B. and Weissman, S., 2012. Trees and Power Lines: Minimizing Conflicts between Electric Power Infrastructure and the Urban Forest. Berkeley Law Center for Law, Energy & the Environment.

NACAA (2015) ‘Chapter 10: Reduce Losses in the Transmission and Distribution Systems’ in Implementing EPA's Clean Power Plan: A Menu of Options, National Association of Clean Air Agencies. National Association of Clean Air Agencies. http://www.4cleanair.org/NACAA_Menu_of_Options

Neale RB, Richter J, Park S, Lauritzen PH, Vavrus SJ, Rasch P, Zhang M. 2013. The mean climate of the community Atmosphere Model (CAM4) in forced SST and fully coupled experiments. Journal of Climate, 26:5150-5168.

Ni M, McCalley J D, Vittal V, Tayyib T. Online risk-based security assessment. IEEE Trans Power Syst 2003;18(1):258–65.

Palutikof, J P, B B Brabson, D H Lister and S T Adcock (1999) A review of methods to calculate extreme wind speeds. Meteorol. Appl. 6, 119–132.

Panteli, M. and Mancarella, P., 2015. Modeling and evaluating the resilience of critical electrical power infrastructure to extreme weather events. IEEE Systems Journal.

Parrish, D.E., 1991. Lightning-caused distribution transformer outages on a Florida distribution system. IEEE Transactions on Power Delivery, 6(2), pp.880-887.

Piantini, Alexandre (2008) Lightning Protection of Overhead Power Distriubtion Lines. 29^th^ International Conference on Lightning Protection, held 23-26 June 2008 in Uppsala Sweden

Pierce, D.W.; Cayan, D.R.; Thrasher, B.L. Statistical downscaling using localized constructed analogs (LOCA). J Hydrometeorology, 2014, 15(6):2558-2585.

Primen (2005). The Cost of Power Disturbances to Industrial and Digital Economy Companies. TR- 1006274 (Available through EPRI).

Quanta-Technology. (2009). Cost-benefit analysis of the deployment of utility infrastructure upgrades and storm hardening programs. Raleigh: Quanta Technology.

Rauber, R. M., and Coauthors, 1999: The Relative Importance of Warm Rain and Melting Processes in Freezing Precipitation Events. Bull. Amer. Meteor. Soc., 39, 1185-1195.

Romps, D., Seeley, J., Vollaro, D., & Molinari, J. (2014). Projected Increase in Lightning Strikes in the United States Due to Global Warming. Science, 346(6211), 851-854.

S&P Global. (2018). S&P Global Platts Electric Power Services. [online] Available at: https://www.spglobal.com/platts/en/products-services/electric-power [Accessed 15 May 2018].

SA.GOV.AU. (2017). Identifying powerlines. [online] Available at: https://www.sa.gov.au/topics/energy-and-environment/electrical-gas-and-plumbing-safety-and-technical-regulation/powerline-safety/identifying-powerlines [Accessed 15 Mar. 2018].

Sanderson B, Knutti R, Caldwell P (2015) A representative democracy to reduce interdependency in a multimodel ensemble. Journal of Climate. doi: 10.1175/JCLI-D-14-00362.1

Sanderson B, Knutti R, Caldwell P (2015) Addressing interdependency in a multi-model ensemble by interpolation of model properties. Journal of Climate. doi: 10.1175/JCLI-D-14-00361.1

Sathaye, Jayant, Larry Dale, Gary Fitts, Peter Larsen, Kevin Koy, Sarah Lewis, and Andre Lucena.. (2011). Estimating Risk to California Energy Infrastructure from Projected Climate Change. California Energy Commission. Publication number: CEC-500-2011-XXX.

Schmidt GA, Ruedy R, Hansen JE, Aleinov I, Bell N, Bauer M, Bauer S, Cairns B, Canuto V, Cheng Y, Del Genio A. 2006. Present-day atmospheric simulations using GISS ModelE: Comparison to in situ, satellite, and reanalysis data. Journal of Climate, 19:153-192.

Seeley, J., & Romps, D. (2017). Regionally-resolved Projections of United States Lightning Strikes. Manuscript in preparation.

Shafieezadeh, A., Onyewuchi, U.P., Begovic, M.M. and DesRoches, R., 2014. Age-dependent fragility models of utility wood poles in power distribution networks against extreme wind hazards. IEEE Transactions on Power Delivery, 29(1), pp.131-139.

Sheffield, J., G. Goteti, and E. F. Wood (2006), Development of a 50-year high-resolution global dataset of meteorological forcings for land surface modeling, J. Climate, 19, 3088–3111.

Sheffield, J., Goteti, G., & Wood, E. (2006). Development of a 50-yr High-resolution Global Dataset of Meteorological Forcings for Land Surface Modeling. Journal of Climate, 19(13), 3088-3111.

Shen, B., Koval, D. and Shen, S., 1999, May. Modelling extreme-weather-related transmission line outages. In Electrical and Computer Engineering, 1999 IEEE Canadian Conference on (Vol. 3, pp. 1271-1276). IEEE.

Sullivan, M., Mercurio, M., & Schellenberg, J. (2009). Estimated Value of Service Reliability for Electric Utility Customers in the United States. Berkeley: Lawrence Berkeley National Laboratory.

Sullivan, M., Schellenberg, J., & Blundell, M. (2015). Updated Value of Service Reliability Estimates for Electric Utility Customers in the United States. Berkeley: Lawrence Berkeley National Laboratory.

Swift et al. 2001. Adaptive Transformer Thermal Overload Protection. IEEE Transactions on Power Delivery 16(4): 516–521.

Taylor KE, Stouffer RJ, Meehl GA (2012) An overview of CMIP5 and the experiment design. Bulletin of the American Meteorological Society. doi:10.1175/BAMS-D-11-00094.1

Taylor, K.; Stouffer, R.; Meehl G. An overview of CMIP5 and the experiment design. Bulletin of the American Meteorological Society, 2012, 93:485-498, DOI: 10.1175/BAMS-D-11-00094.1.

U.S. Bureau of Reclamation et al. Downscaled CMIP3 and CMIP5 climate projections –addendum release of downscaled CMIP5 climate projections (LOCA) and comparison with preceding information. September 2016, data available here: http://gdo-dcp.ucllnl.org/downscaled_cmip_projections/

U.S.-Canada Power System Outage Task Force. (2004). Final Report on the August 14, 2003 Blackout in the United States and Canada: Causes and Recommendations. [online] U.S.-Canada Power System Outage Task Force, p. 59. Available at: https://www3.epa.gov/region1/npdes/merrimackstation/pdfs/ar/AR-1165.pdf [Accessed 3 Aug 2018].

Vermont Open Geodata Portal. (2016). Vermont Open Geodata Portal. [online] Available at: http://geodata.vermont.gov/ [Accessed 26 Apr. 2018].

von Salzen K, Scinocca JF, McFarlane NA, Li J, Cole JN, Plummer D, Verseghy D, Reader MC, Ma X, Lazare M, Solheim L. 2013. The Canadian fourth generation atmospheric global climate model (CanAM4). Part I: representation of physical processes. Atmosphere-Ocean, 51:104-125.

Wang, M., A.J. Vandermaar, K.D. Srivastava (2002) Review of Condition Assessment of Power Transformers in Service. Electrical Insulation 0883-7554/02.

Ward, D.M., 2013. The effect of weather on grid systems and the reliability of electricity supply. Climatic change, 121(1), pp.103-113.

Watanabe M, Suzuki T, O'ishi R, Komuro Y, Watanabe S, Emori S, Takemura T, Chikira M, Ogura T, Sekiguchi M, Takata K. 2010. Improved climate simulation by MIROC5: mean states, variability, and climate sensitivity. Journal of Climate 23:6312-6335.

Weggel JR. 1999. Maximum daily wind gusts related to mean daily wind speed. Journal of Structural Engineering 125: 465–468.

Wobus, C., Gutmann, E., Jones, R., Rissing, M., Mizukami, N., Lorie, M., Mahoney, H., Wood, A. W., Mills, D., and Martinich, J.: Climate change impacts on flood risk and asset damages within mapped 100-year floodplains of the contiguous United States, Nat. Hazards Earth Syst. Sci., 17, 2199-2211, https://doi.org/10.5194/nhess-17-2199-2017, 2017.

Wolfe, R. and Kluge, R. O. (2005) Designated Fiber Stress of Wood Poles, Forest Products Laboratory. U.S. Department of Agriculture.

Wu, T., Ruan, J., Hu, Y., Liu, B. and Chen, C., 2011, December. Study on forest fire induced breakdown of 500 kV transmission line in terms of characteristics and mechanism. In Zhongguo Dianji Gongcheng Xuebao(Proceedings of the Chinese Society of Electrical Engineering) (Vol. 31, No. 34, pp. 163-170). Chinese Society for Electrical Engineering.

Yue, X., Mickley, L.J., Logan, J.A. and Kaplan, J.O., 2013. Ensemble projections of wildfire activity and carbonaceous aerosol concentrations over the western United States in the mid-21st century. Atmospheric Environment, 77, pp.767-780.

1. These scatter plots were developed using the LASSO tool, a product of EPA’s Office of Research and Development – National Center for Environmental Assessment. [↑](#footnote-ref-1)
2. A number of the GCMs in the plots contain multiple initializations that are designed with numbers in subscript. The dashed lines represent the median value for each axis. [↑](#footnote-ref-2)
